# Bacon: a comprehensive computational benchmarking framework for evaluating targeted chromatin conformation capture-specific methodologies

**DOI:** 10.1186/s13059-021-02597-4

**Published:** 2022-01-21

**Authors:** Li Tang, Matthew C. Hill, Patrick T. Ellinor, Min Li

**Affiliations:** 1 Hunan Provincial Key Lab on Bioinformatics, School of Computer Science and Engineering, Central South University, Changsha, 410083 China; 2 Cardiovascular Research Center, Massachusetts General Hospital, Boston, MA 02129 USA; 3 Cardiovascular Disease Initiative, The Broad Institute of MIT and Harvard, Cambridge, MA 02142 USA

**Keywords:** Chromatin topology, HiChIP, ChIA-PET, Looping, Benchmark

## Abstract

Chromatin conformation capture (3C)-based technologies have enabled the accurate detection of topological genomic interactions, and the adoption of ChIP techniques to 3C-based protocols makes it possible to identify long-range interactions. To analyze these large and complex datasets, computational methods are undergoing rapid and expansive evolution. Thus, a thorough evaluation of these analytical pipelines is necessary to identify which commonly used algorithms and processing pipelines need to be improved. Here we present a comprehensive benchmark framework, Bacon, to evaluate the performance of several computational methods. Finally, we provide practical recommendations for users working with HiChIP and/or ChIA-PET analyses.

**Supplementary Information:**

The online version contains supplementary material available at 10.1186/s13059-021-02597-4.

## Background

There is sufficient evidence that genomic organization, whereby protein complexes contribute to the formation of long-range physical contacts between distal regulatory elements, plays an important role in dictating gene expression patterns [1, 2]. Many regulatory elements dictate target gene transcription over large genomic distances (up to millions of base pairs), making it a great challenge to detect which regulatory elements control which genes [3]. The development of chromosome conformation capture (3C)-based technologies [4–10] now makes it possible to detect such long-range genomic interactions at high resolution. Moreover, these technologies have uncovered new principles of genome organization, including the discovery of topologically associated domains (TADs) or contact domains [11, 12], genome compartments, and interactions that physically link the regulatory elements of the genome [13], like enhancer-promoter interactions [14–16]. Chromatin interaction analysis by paired-end tag sequencing (ChIA-PET) is a technique which combines ChIP, 3C, and next-generation sequencing, allowing the identification of long-range contacts bound by a transcription factor or chromatin mark of interest [17]. The recently developed long-reads ChIA-PET [18] protocol provides better resolution and requires lower chromatin input than traditional ChIA-PET. HiChIP [19] and PLAC-seq [20] were both developed to improve the efficiency and sensitivity of ChIA-PET through the implementation of transposase-mediated library construction. However, it is a challenge to interpret targeted conformation capture data quantitatively, owing to the high prevalence of sequenced ligation junctions formed from uninformative close-range contacts. Moreover, the data analysis is complicated by the relative inefficiency of chromatin immunoprecipitation-based methods, which often lead to low library complexity. Therefore, robust and efficient computational methods are required to remove the biases associated with these molecular protocols and more accurately quantify chromatin contacts.

The ChIA-PET Tool [21] first proposed a general pipeline for processing of ChIA-PET data, including linker trimming, read alignment, loop detection, and significance estimation. The ChIA-PET Tool uses a hypergeometric (HG) distribution to count loops, and the HG model assumes that the random pairing chance of two anchor regions increases as the sequencing depth of the two anchor regions increases. Several subsequent methods were mostly based on this underlying procedure with only slight modifications. ChiaSig [22] improved the model by employing a non-central HG distribution, and the model added an additional factor, the distance between two anchor regions. The Model-based Interaction Calling from ChIA-PET (MICC) uses a Bayesian mixture model to systematically remove random ligation and random collision noise [23]. Another popular computational pipeline, mango [24], uses a binomial model to detect statistically significant interactions for ChIA-PET data, and also corrects for major sources of ChIA-PET data bias, including differential peak enrichment and genomic proximity. ChIA-PET2 [25] took a Bayesian mixture model to provide a flexible pipeline for analyzing different types of ChIA-PET data, and it also supports allele-specific analyses. ChIA-PET Tool V3 [26] is an updated version of the ChIA-PET Tool, which processes short-read and long-read ChIA-PET data with multithreads. A recently developed method ChIAPoP [27] uses positive Poisson to distinguish the significant interactions from noisy ChIA-PET data. Hichipper [28] employs a background model to identify loops, which incorporates the effect of restriction enzyme site bias. MAPS [29] adopted a zero-truncated Poisson regression framework to explicitly remove the biases of HiChIP/PLAC-seq data, and then identifies the chromatin interactions by the normalized contact frequencies. FitHiChIP [30] leverages the non-uniform coverage and genomic distance scaling of contact counts to compute the significance of estimated loops. Also, HiCCUPS is a loop caller developed for Hi-C data, which also can be used to call HiChIP loops [31].

All the computational methods mentioned above are peak-based and tend to integrate the popular peak calling algorithm MACS2 [32] or similar pipelines to facilitate the positioning of loop anchors. However, given the protocol differences, many of the peak-based computational methods cannot be applied to ChIA-PET and HiChIP data simultaneously. Accordingly, cluster-based methods were developed to fit both types of datasets. cLoops [33] was based on the clustering algorithm cDBSCAN, which takes Paired-End Tags (PETs) and analyzes them directly by a permuted local background to estimate significance. A similar computational pipeline, CID [34], discovers chromatin interactions with an unbiased clustering approach that identifies loop anchors by splitting the PET groups iteratively. Recently, several benchmarking studies on Hi-C methods have been published [35–37]; however, computational benchmarking for targeted chromatin conformation capture-specific methodologies are lacking.

With this in mind, we present a comprehensive benchmark framework, Bacon, to evaluate the performance of targeted chromatin conformation capture-specific methodologies. Due to the intrinsic biases that exist for targeted conformation data, we systematically characterized the differences between two closely related technologies (ChIA-PET and HiChIP) and built the Bacon framework based on the established distinctions. In this study, we benchmarked 12 computational pipelines using 22 experimental datasets and 6 simulations. Finally, Bacon provides practical guidance for users and aids in the rational development of improved pipelines for developers.

## Results

### Data characteristics of ChIA-PET and HiChIP

**Fig. 1:**
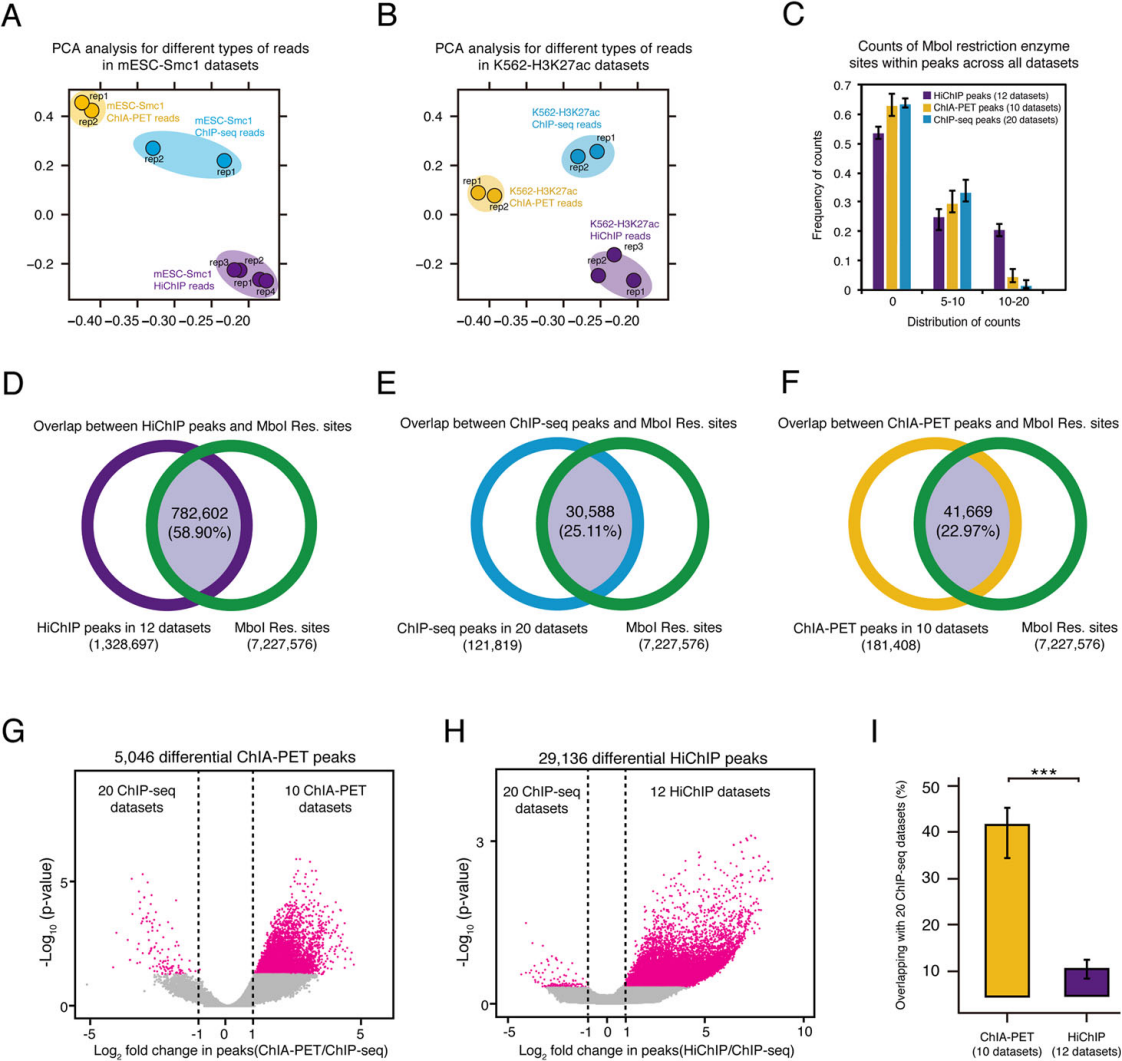
Data characteristics of ChIA-PET and HiChIP. **A** PCA plot for replicates of mESC-Smc1 ChIP-seq, ChIA-PET, and HiChIP reads. **B** PCA plot for replicates of K562-H3K27ac ChIP-seq, ChIA-PET, HiChIP reads. **C** The count distribution of Mbol restriction enzyme sites across different peak types on 12 HiChIP, 10 ChIA-PET, and 20 ChIP-seq datasets. **D** The overlap between HiChIP peaks and Mbol restriction sites across 12 HiChIP datasets. **E** The overlap between ChIP-seq peaks and Mbol restriction sites across 20 ChIP-seq datasets. **F** The overlap between ChIA-PET peaks and Mbol restriction sites across 10 ChIA-PET datasets. **G, H** Differential volcano plots comparing ChIP-seq with ChIA-PET and HiChIP peaks quantitatively on 12 HiChIP, 10 ChIA-PET, and 20 ChIP-seq datasets. **I** Peak co-occupancy between ChIP-seq/ChIA-PET and ChIP-seq/HiChIP on 12 HiChIP, 10 ChIA-PET, and 20 ChIP-seq datasets. Bar plot drawn with standard error bar. ***, *p* value < 1e−3, *p* value was calculated by *t*-test

HiChIP and ChIA-PET both produce similar information about protein-specific topological interactions and are interpreted in much the same way. However, the two protocols differ greatly in how they capture this information. One important distinction between the two is that HiChIP incorporates restriction endonucleases to fragment the genome, while ChIA-PET traditionally relies upon sonication. And while this difference has been taken into account by some computational pipelines [28], a full characterization of the differences and similarities that exist between HiChIP and ChIA-PET is lacking. To characterize the read properties of HiChIP and ChIA-PET libraries, we compared their alignments with publicly available ChIP-seq data (for the processing of ChIP-seq data, see “Methods”). Principal component analysis (PCA) showed that the ChIP-seq, ChIA-PET, and HiChIP replicates clustered together into their respective experimental groups (Fig. 1A, B) (for PCA analysis, see “Methods”). Notably, mESC-Smc1 HiChIP data was heavily impacted by restriction enzyme treatment, as the read distribution of mESC-Smc1 HiChIP presented restriction enzyme cut site bias. Correspondingly, mESC-Smc1 HiChIP reads were sparse in positions without Mbol restriction sites (Additional file 1: Fig. S1A). To compare the bias of these two methodologies in a quantitative manner, we called peaks for 20 ChIP-seq, 10 ChIA-PET, and 12 HiChIP datasets separately, and then calculated the distribution of Mbol restriction enzyme motifs near peaks. We found the count distribution of restriction enzyme sites also showed more enzyme sites located within HiChIP peaks (Fig. 1C). Overall, 58.9% of HiChIP peaks overlapped with Mbol restriction enzyme sites (Fig. 1D-F), which was much greater than the 22.9% observed for ChIA-PET.

An important control for evaluating HiChIP and ChIA-PET experiments is a favorable overlap with ChIP-seq data. We next performed differential analysis between 10 ChIA-PET, 12 HiChIP, and 20 ChIP-seq datasets (See “Methods”). A total of 5046 peaks that differed significantly between ChIA-PET and ChIP-seq were identified, and 3549 of these were ChIA-PET enriched (Fig. 1G). Conversely, 29,136 peaks differed between ChIP-seq and HiChIP datasets, among these, 26,222 were HiChIP-specific (Fig. 1H). We then looked at the peaks in each dataset had in common with ChIP-seq and found that 41.6% of ChIA-PET peaks overlapped with ChIP-seq peaks compared to 10% overlap of ChIP-seq with HiChIP peaks (Fig. 1I). Hence, HiChIP data generates more loops with elevated sensitivity compared to ChIA-PET; however, HiChIP produces data with a strong restriction enzyme site bias and a lower overall agreement with ChIP-seq data than ChIA-PET.

### A benchmark framework for targeted chromatin conformation capture-specific methods

**Fig. 2:**
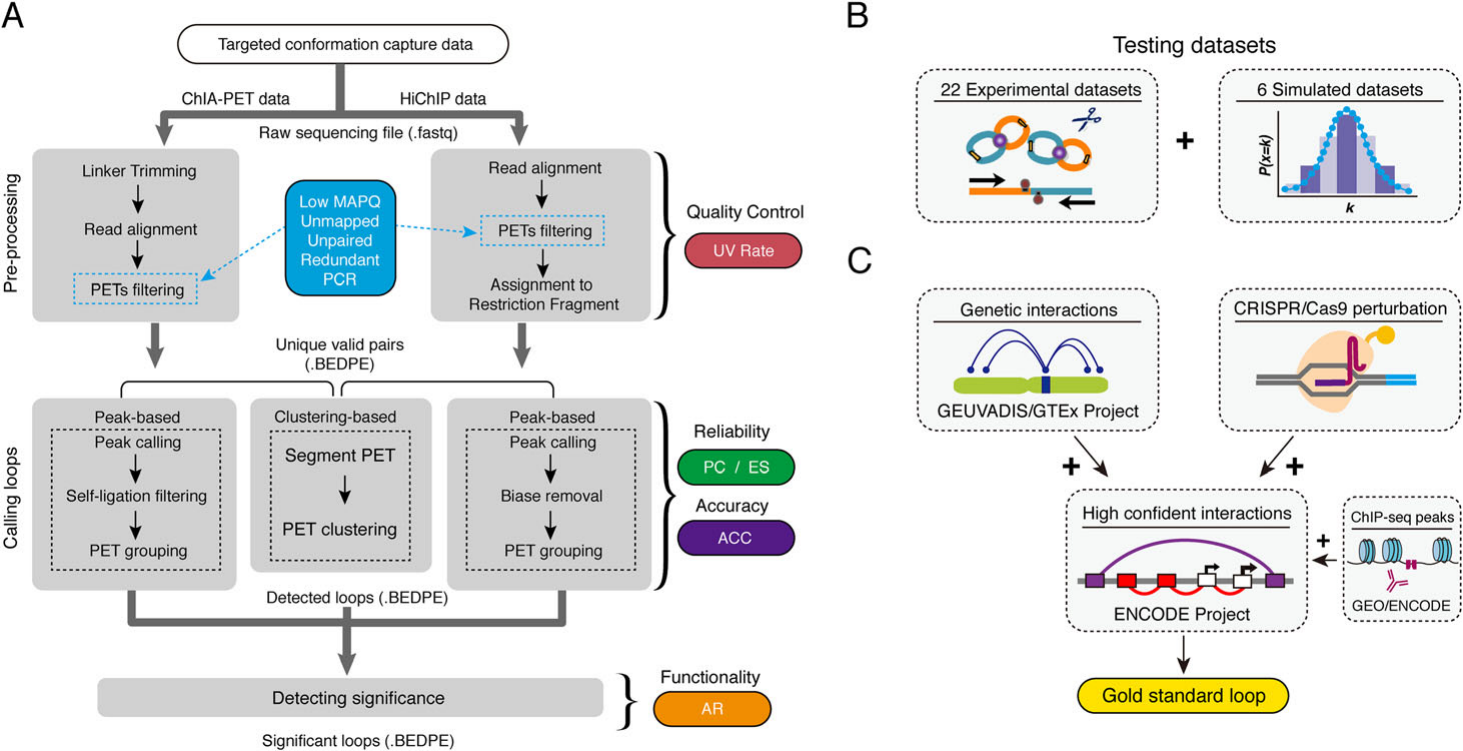
The schema of Bacon. **A** Overview of approach. The processing steps are connected by arrows, blue squares indicate the categories of low-quality PETs to be filtered, and UV Rate, PC/ES, ACC, and AR are the evaluation metrics employed by Bacon to estimate the performance of different methods. **B** The testing datasets integrated by Bacon. **C** Schematic displaying how gold standard loop sets were gathered to evaluate the accuracy of different methodologies

In the current work, we developed Bacon, a computational benchmark framework that enables the characterization of the analysis steps for targeted chromatin conformation capture data, and the evaluation of the performance of different computational methods. Bacon addresses three fundamental processing steps for ChIA-PET and HiChIP datasets, including pre-processing, loop calling, and the detection of significant interactions (Fig. 2A and Additional file 1: Fig. S2). Our framework also provides an evaluation of 12 different computational methods (Additional file 1: Table S1). Further, Bacon integrates 28 ChIA-PET and HiChIP datasets for testing (22 experimental, and 6 simulated datasets) (Fig. 2B and Additional file 2: Table S6) and gathers gold standard interactions from the GEUVADIS Project [38], GTEx Project [39], CRISPRi perturbation screening [40], and ENCODE [41] for evaluating accuracy (Fig. 2C).

Bacon uses the Uniquely Valid Rate (UV Rate) (for the calculation of UV Rate, see “Methods”) to evaluate the quality of wet-lab experiments, and the pre-processing effectiveness of each computational method. For loop calling, Bacon evaluates the reliability of anchors, as well as the accuracy of loops. The two state-of-the-art strategies to identify HiChIP and ChIA-PET loops are peak-based and cluster-based methods. In general, the peak-based methods start with peak calling by implementing MACS2 or other peak calling algorithms. Bacon utilizes peak co-occupancy (PC) (for the calculation of PC, see “Methods”) to evaluate the reliability of anchors identified by the peak-based methods. Cluster-based methods typically use the read density or the distance within two paired-end tags (PETs) to identify loops. Bacon evaluates the enrichment levels of cluster-based anchors using an enrichment score (ES) (for the calculation of ES, see “Methods”). The accuracy of loops (ACC) is evaluated through the comparison of each output to a gold standard loop set (for the collection of gold standard loops and calculation of ACC, see “Methods”).

To detect statistically significant genomic interactions, different strategies are applied by the current computational methods. Bacon validates the functionality of significant loops by calculating the activation rate (AR) (see “Methods”), which estimates the epigenetic functionality of loops through the incorporation of several active histone markers, such as H3K27ac, H3K4me1, and H3K4me3.

### Evaluating the reliability of loop anchors

**Fig. 3:**
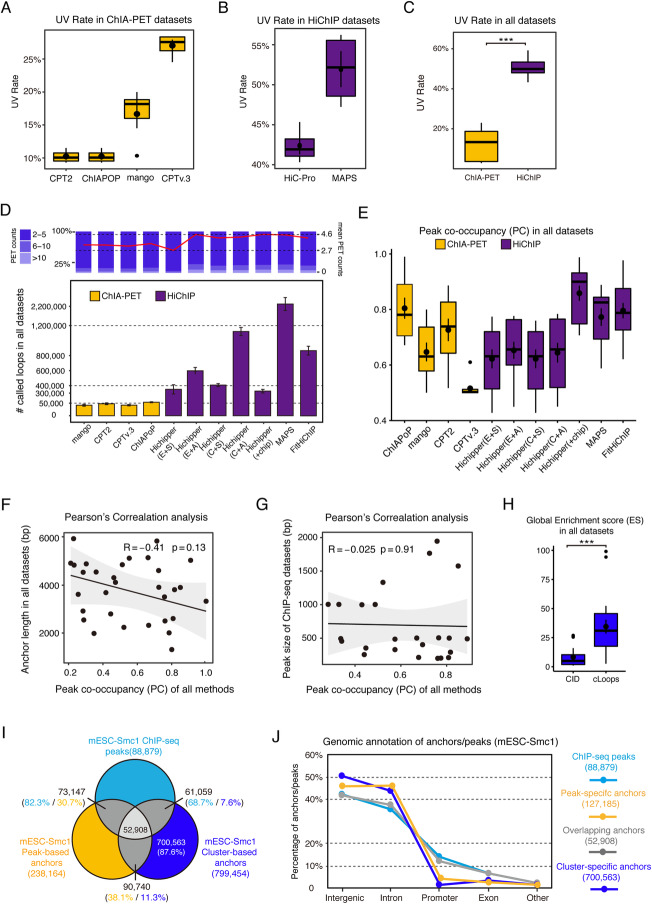
Evaluating the reliability of loop anchors. **A** UV Rate of ChIA-PET analytical methods across all ChIA-PET datasets. **B** UV rate of HiChIP analytical methods across all HiChIP datasets. **C** Comparison of UV Rate between ChIA-PET and HiChIP datasets. **D** Number of loops identified with different computational methods across all datasets. Bar graph displays the distribution of PET counts. The red line represents the average PET counts for the corresponding loop sets. **E** The peak co-occupancy (PC) of peak-based loop anchors. **F** Pearson’s correlation between PC and anchor length. **G** Pearson’s correlation between PC and peak size. *R*, correlation coefficient. *p* value calculated by *t*-test. **H** Global ES of two cluster-based methods in all datasets. *** *p* value < 1e−3, *p* value was calculated by *t*-test. **I** The overlap between mESC-Smc1 ChIP-seq peaks, peak-based anchors, and cluster-based anchors. **J** The genomic annotation of mESC-Smc1 ChIP-seq peaks, peak-based anchors, overlapping anchors, and cluster-specific anchors. All the error bars represent the calculated mean and standard error

To evaluate the pre-processing steps required for any given method, Bacon calculates the UV Rate to estimate the percentage of valid PETs which uniquely map to the reference genome. Bacon considered 0 mismatched and 1 mismatched base pair during linker trimming for ChIA-PET2(CPT2) and ChIAPoP, mango incorporates a fixed setting, and for ChIA-PET Tool V3 (CPTv.3) Bacon used the default linker alignment score (Additional file 1: Table S2 and Fig. S3). The alignment score (MAPQ) was set to 30 to filter out the high-quality PETs. Among all the ChIA-PET datasets we evaluated, CPTv.3 generated the highest UV Rate, followed by mango (Fig. 3A). For HiChIP analysis, only HiC-Pro and MAPS include data pre-processing, and MAPS achieved a higher UV Rate than HiC-Pro (Fig. 3B). HiC-Pro was developed to analyze Hi-C data and as part of its’ two-step alignment process removes dumped pairs, dangling ends, and self-circles. This effectively decreases the total number of unique valid PET output from HiC-Pro, at least partially explaining its reduced UV Rate when compared to MAPS which incorporates these categories of pairs. Moreover, the UV Rate of HiChIP data ranged from 43.28 to 59.18%, which was greater than the UV Rate output from ChIA-PET data (3.23–22.95%) (Fig. 3C). These results suggest that the sensitivity of HiChIP is greater than ChIA-PET, which is consistent with previous studies [19, 42].

Within the framework of Bacon, loops are called from different peak-based and cluster-based methods separately. In accordance with the results of Fig. 3C, the number of loops detected from HiChIP data was greater than for ChIA-PET (Fig. 3D). Among the peak-based methods we compared, MAPS supported the input of reference peaks (high-quality ChIP-seq peaks) to facilitate loop detection and remove noise. Utilizing a conserved strategy, MAPS retains not only the pairs with both ends overlapping a reference peak, but also the pairs with only one end overlapping. Hence, the number of loops called by MAPS ranked first among all the pipelines we evaluated. A HiChIP-specific software package known as Hichipper supports three types of inputs: reference peaks, only self- and dangling peaks, and all HiChIP peaks (Additional file 1: Fig. S4). We compared all the various settings for Hichipper and found that HiChIP (All peaks) and the combination of all replicates as input produced the greatest number of significant loops with this pipeline (Fig. 3D). Currently, there are very few cluster-based methods available. Only CID [34] and cLoops packages [33] are applicable for performing both HiChIP and ChIA-PET analysis, so we chose these to evaluate with Bacon. The results showed that both algorithms generated more loops when applied to HiChIP data compared to ChIA-PET datasets. The analysis of cluster-based results also indicated that CID produced more loops than cLoops (Additional file 1: Fig. S5).

To evaluate the reliability of peak-based loop anchors, Bacon calculates peak co-occupancy (PC) to estimate the overlapping percentage between loop anchors and ChIP-seq peaks. The results of PC analysis showed that ChIAPoP achieved the highest occupancy with published ChIP-seq peaks across all of the ChIA-PET analytical methods, and Hichipper(+chip) achieved the best among all of the HiChIP analytical methods followed by MAPS and FitHiChIP (Fig. 3E), since these three methods took ChIP-seq peaks as input to detect loops. In addition, peak intensity analysis of loop anchors showed that ChIAPoP and Hichipper(+chip) achieved higher peak intensity values than all the other methods (Additional file 1: Fig. S8). To investigate whether PC was impacted by the length of the anchor or the size of peak, we performed Pearson’s correlation analysis between PC and the length of anchor/the size of peak; however, the results did not show significant correlations (Fig. 3F,G).

To estimate the reliability of cluster-based loop anchors, we developed Bacon to calculate the enrichment score (ES) for the loop anchors generated by CID and cLoops. ES was calculated via a site-by-site evaluation, which indicates whether the observed enriched site is significantly enriched, and the global ES reflects the average accuracy of the loops from the whole genome. Overall, we found that cLoops achieved a higher global ES than CID for the datasets we evaluated, indicating cLoops can detect more highly enriched (strong) loops; however, there still exists the possibility that CID is more sensitive in identifying true-weak loops (Fig. 3H and Additional file 1: Fig. S6-S7).

To investigate the differences between peak-based anchors and cluster-based anchors, we combined the loop anchors detected by all the peak-based methods of mESC-Smc1 ChIA-PET/HiChIP datasets, and also combined the loop anchors detected by the cluster-based methods. The adjacent loop anchors within each set were merged and duplicates were removed. The overlapping results showed that 30.7% of peak-based anchors overlapped with ChIP-seq peaks, while only 7.6% of cluster-based anchors overlapped with ChIP-seq peaks, and 87.6% of the cluster-based anchors were specific (Fig. 3I). We next annotated the ChIP-seq peaks, the overlapping anchors derived from each of the three specific loop sets, and cluster-specific anchors. The annotations indicated that cluster-specific anchors were less enriched at promoters (Fig. 3J), which suggests that cluster-based methods detect more loops within non-coding regions in the datasets we analyzed.

To further investigate the properties of the different loops called by these methods, we chose ChIAPoP as the representative ChIA-PET peak-based method and compared the ChIAPoP-specific loops, cLoops-specific loops, and CID-specific loops with active and inactive histone mark ChIP-seq data. The results showed that ChIAPoP produces more active chromatin-enriched peaks with higher H3K27ac signal than cLoops and CID, while cLoops and CID output loops with more inactive H3K27me3-enriched profiles (Additional file 1: Fig. S9A) in the dataset we analyzed. For the analysis of HiChIP, we chose Hichipper(+chip) as representative, and the comparisons showed similar results to what was observed for ChIA-PET (Additional file 1: Fig. S9B).

### Evaluating the accuracy of loops

To evaluate the loops generated by different methods in a quantitative manner, we gathered cell type-specific long-range contacts from our gold standard loop sets. To ensure the fairness of comparison, we generated three gold standard loop sets for each testing dataset (for the gathering of gold standard loop set, see “Methods”). Accuracy (ACC) was calculated for True Positive (TP), False Positive (FP), True Negative (TN), and False Negative (FN) metrics (for the calculation of ACC, see “Methods”), for better comparison and visualization, we re-scaled ACC from 0 to 1.

**Fig. 4:**
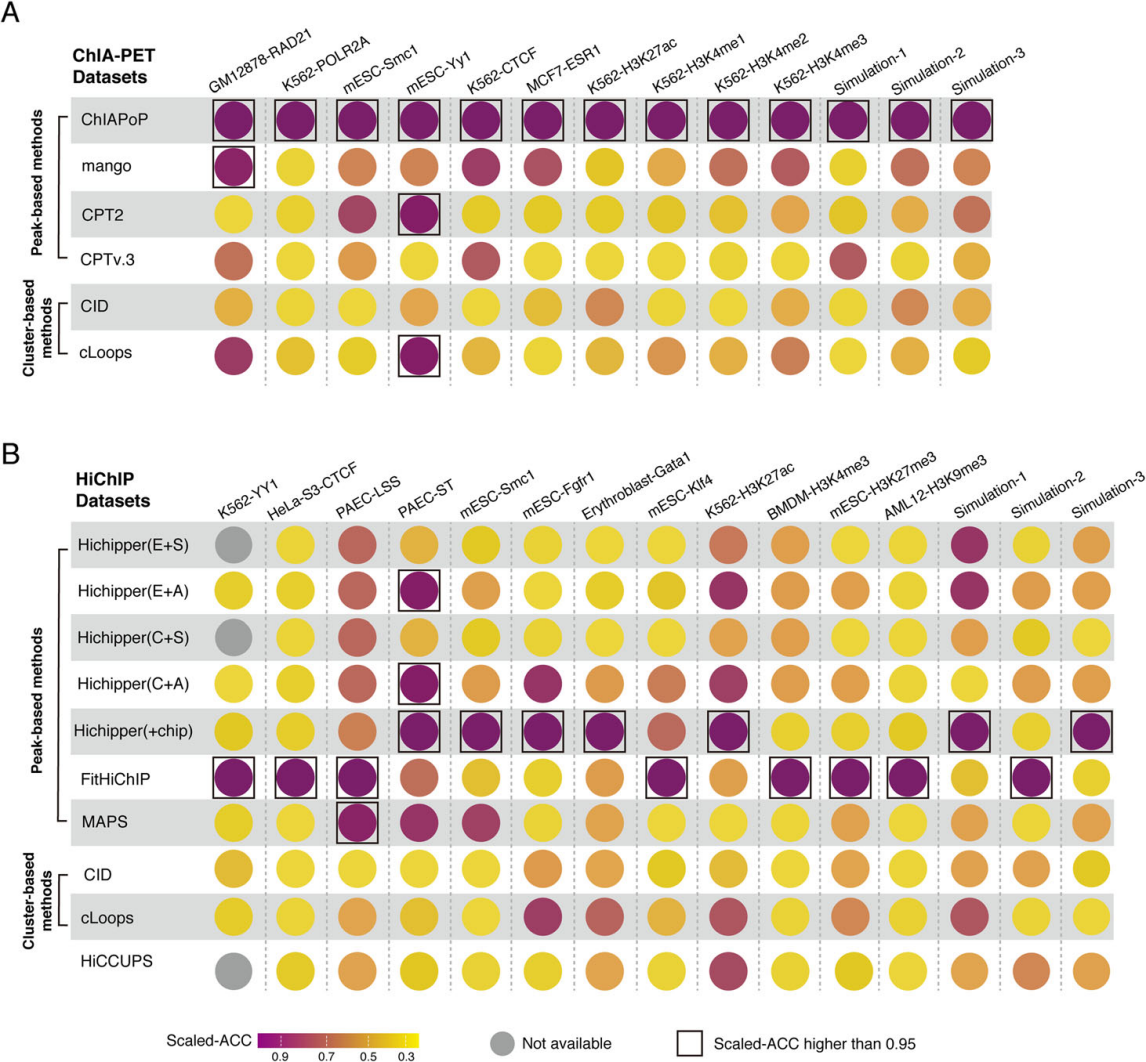
Evaluating the accuracy of loops. **A, B** Dot plot displaying the scaled accuracy of loops. A gray circle indicates that the corresponding method failed to detect loops. The dark squares indicate that the scaled accuracy is greater than 0.95

For the ACC evaluation results of ChIA-PET datasets, ChIAPoP outperformed the other ChIA-PET analytical methods with high scaled-ACC (> 0.95) in all datasets. For HiChIP methods, FitHiChIP and Hichipper(+chip) performed better than the others, which achieved the high scaled-ACC (> 0.95) in 8 and 7 datasets (Fig. 4) (for raw ACC, see Additional file 3: Table S7, and Additional file 4: Table S8).

Although there were more HiChIP loops than ChIA-PET loops (Fig. 3D), the ACC of loops was independent of the number of loops (Additional file 1: Fig. S10A). The ACC of ChIA-PET loops was higher than that of HiChIP loops across all the testing datasets (Additional file 1: Fig. S10B). To investigate what impacted the results of ACC, we calculated Pearson’s correlation coefficient for ACC and three other evaluation metrics. The results suggested that UV Rate barely correlated with ACC (Additional file 1: Fig. S10C), while PC and ES were positively correlated with ACC (Additional file 1: Fig. S10D and S10E).

### Functionality of statistically significant loops

To remove noise and improve the accuracy of detected loops, we next wanted to apply a variety of statistical methods to the final loop outputs produced by each analytical method. Current analysis packages employ different strategies to identify the significance of loops, for example, ChiaSig facilitated non-central hypergeometric (NCHG) distribution [22], and mango employs corrected *p* values to account for multiple hypothesis testing [24]. Since CID can only call loops, as suggested by Guo et al. [34], we utilized the MICC tool [23] to identify the significance of CID loops. FitHiChIP provides two types of background model (loose L or stringent S) to correct biases, here represented by FitHiChIP-L and FitHiChIP-S.

**Fig. 5:**
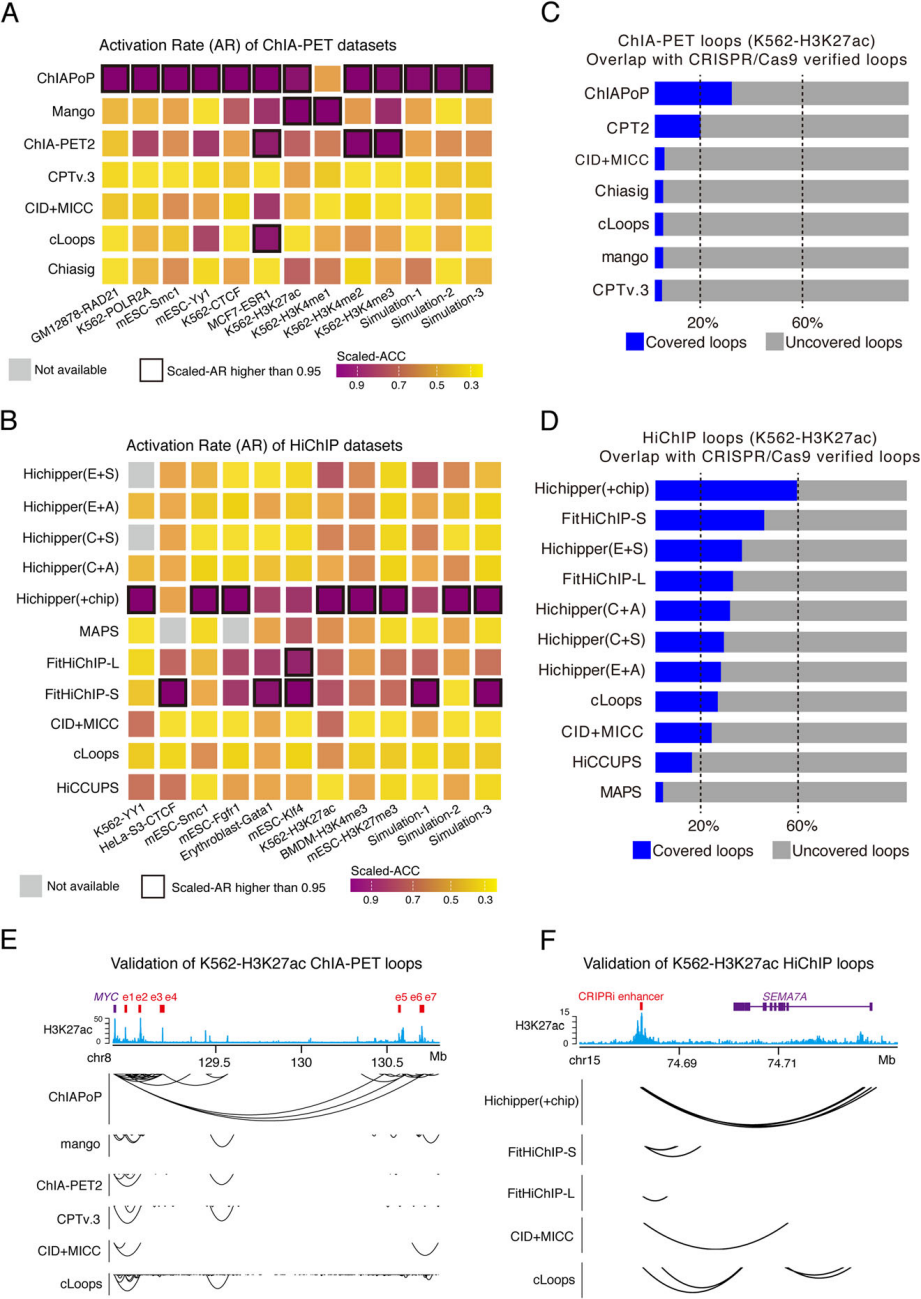
Functionality of statistically significant loops. **A** Activation rate of ChIA-PET analytical methods across all ChIA-PET testing datasets. **B** Activation rate of HiChIP analytical methods across all HiChIP testing datasets. **C** Overlap between ChIA-PET K562-H3K27ac loops and CRISPR/Cas9 verified loops. **D** Overlap between HiChIP K562-H3K27ac loops and CRISPR/Cas9 verified loops. **E** Example of enhancer-*MYC* interactions from K562-H3K27ac ChIA-PET datasets. **F** Example of enhancer-*SEMA7A* interactions from K562-H3K27ac HiChIP datasets. CID + MICC, indicates the combination of CID loop calling and significance detection with the MICC package. The gray square indicates that the corresponding method failed to detect loops. The dark squares indicate that the scaled accuracy was larger than 0.95

To compare the significant loops fairly, we firstly counted the number of significant loops with at least 3 PETs from ChIA-PET, at least 8 PETs with HiChIP data, and then set a *p* value threshold of 0.05 (false discovery rate (FDR) of 0.05 if accessible for the method). To determine the properties and to detect the functionality of these significant loops, we utilized candidate enhancer-like and promoter-like signatures from ENCODE [41] to annotate loops. Next, H3K27ac, H3K4me1, H3K4me3, and H3K27me3 ChIP-seq datasets were used to calculate the activation rate (AR) of enhancer-mediated loops (for the calculation of AR, see “Methods”), for better comparison and visualization, we re-scaled AR from 0 to 1. We then determined the AR of each individual method and found that ChIAPoP obtained the best AR in 12 ChIA-PET datasets, and Hichipper(+chip) obtained the highest AR for 8 of the HiChIP datasets, FitHiChIP-S achieved the highest AR for 5 of the HiChIP datasets (Fig. 5A, B).

To more rigorously assess the functional significance of the output loops from each analytical pipeline, we overlapped the detected loops with large-scale CRISPRi perturbation screening data obtained from K562 cells [40]. We found that ChIAPoP achieved the highest overlapping percentage in ChIA-PET (Fig. 5C), and Hichipper(+chip) performed best in HiChIP datasets, followed by FitHiChIP-S (Fig. 5D). Further, we interrogated the well-studied MYC locus with K562-H3K27ac ChIA-PET data, and we found that most methods detected contacts that overlapped with a set of previously validated MYC enhancers [43]. Notably, ChIAPoP loops contacted more CRISPRi MYC enhancers than the other methods, such as *MYC*-enhancer5, *MYC*-enhancer6, and *MYC*-enhancer7 (Fig. 5E). Secondly, we evaluated K562-H3K27ac HiChIP data against another set of CRISPRi validated loops [40], and we found that there were four methods detecting loops near the SEMA7A locus; however, only Hichipper(+chip) identified loops between this CRIPSRi-validated distal enhancer and the SEMA7A promoter region (Fig. 5F).

## Discussion

The emergence of 3C-based techniques has enabled the accurate detection of 3D genomic interactions. Importantly, 3D genomic contacts are highly dynamic given the variability of chromosome structure [44, 45]. In addition, 3C protocols take an average view of the chromatin interactions from a population of cells, and the limitations of penetrance may lead to the low availability of appreciable contacts, which hinders the interpretation of biological function. The adoption of ChIP techniques makes it possible to identify rare interactions mediated by a protein of interest, which are often undetectable by other 3C-based methods. Currently, it is a challenge to interpret targeted conformation capture data quantitatively. Moreover, low levels of ChIP enrichment often reduce the complexity of HiChIP and ChIA-PET libraries. Given these challenges, we developed Bacon, a benchmark framework to facilitate the comparison of computational methods and provide practical guidance for users and suggestions for the rational design of new analytical pipelines.

**Fig. 6:**
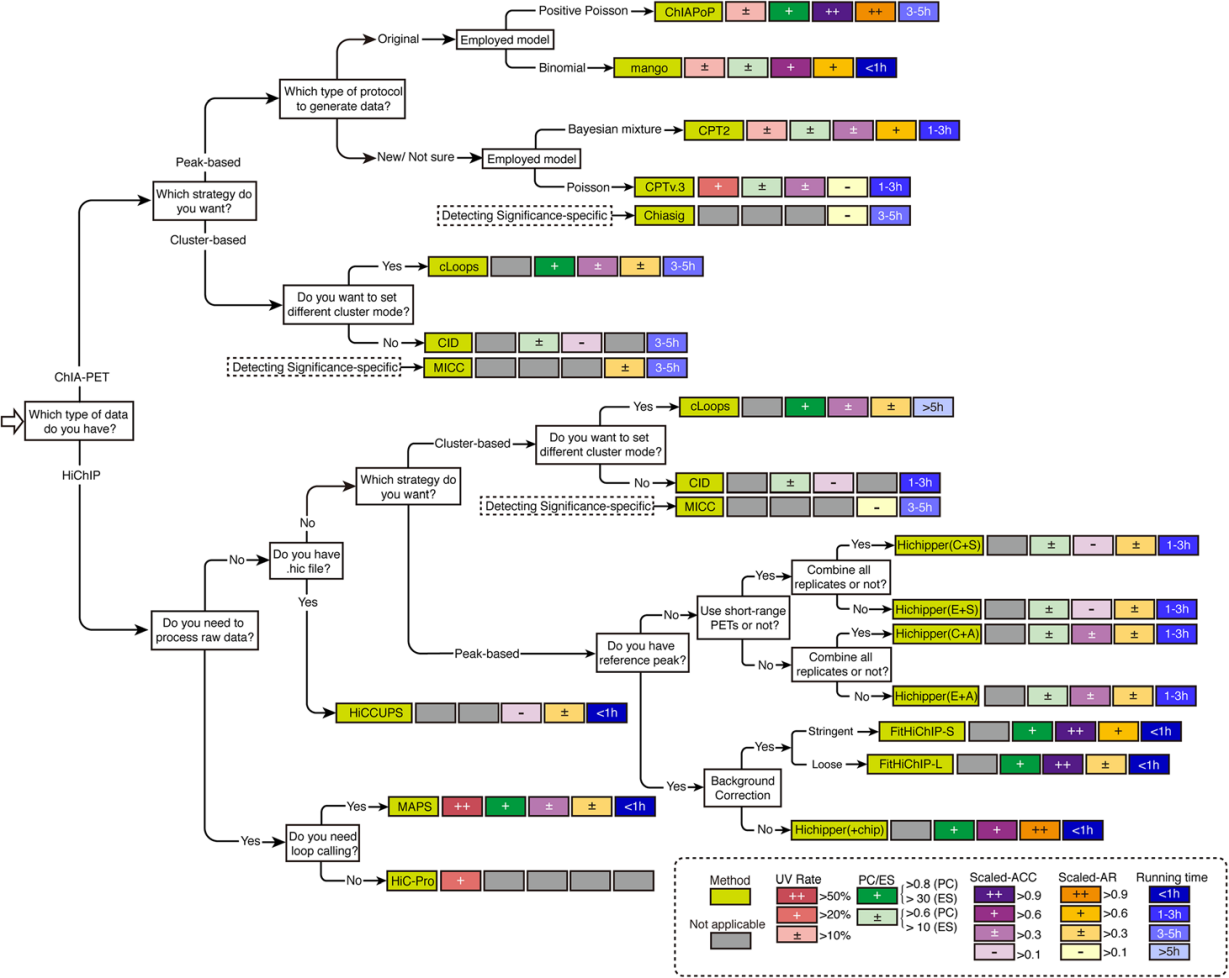
Practical guidance for method users. The average UV Rate, PC/ES, scaled-ACC, scaled-AR, and running time were calculated across all datasets. The results are annotated by color. The average running time only included calling loops and detecting significance across all testing datasets. Testing environment is as follows: Intel(R) Xeon(R) CPU E5-2650 v3 @ 2.30 GHz, 6 cores, 128 GB. The gray square denotes that the corresponding method fails to support the indicated step in the analytical process

To provide practical guidance for users, we considered different conditions, different analysis strategies, and the performance of each tool (Fig. 6). The mean UV Rate, PC/ES, scaled-ACC, and scaled-AR in all datasets were calculated and then annotated based on how well they performed across multiple data sets. We also recorded the running time for each loop calling method across different datasets (Additional file 1: Table S4-S5). For ChIA-PET analysis, ChIAPoP outperformed the others in reliability, accuracy and detecting activation; however, ChIAPoP required more running time than the other peak-based methods, and cannot be applied to datasets generated by long-read ChIA-PET protocols. For the HiChIP analytical methods, FitHiChIP-S and Hichipper(+chip) outperformed the others in PC, ACC, AR, and running time. However, both FitHiChIP and Hichipper only accept the valid pairs output from HiC-Pro to call loops, so if users want to perform the analysis procedure without switching methods, then MAPS is the only choice. We noticed that although cluster-based methods (CID and cLoops) can be applied to both ChIA-PET and HiChIP datasets, the ACC and AR metrics did not stand out, and these two methods also took more running time than the peak-based methods.

Here we provided several suggestions for the future development analytical pipelines for both HiChIP and ChIA-PET analysis. Importantly, more flexible parameters should be considered, such that future chemistries, sequencing read lengths, and other experiment-specific factors can be appropriately accounted for. We found that the methods we analyzed integrated different alignment tools, and the key mapping parameters were fixed by most pipelines. However, for different lengths of raw sequencing data, the alignment settings should be adjustable to achieve optimal results. Additionally, more reasonable self-ligation cutoffs should be set, such that the reads being input for loop calling are completely valid. Self-ligation PETs are filtered out prior to calling loops, and the cutoff between a read PET being designated as a self-ligation product versus inter-ligation product ranges from 5 to 12 kb [21, 33]. While most methods simply set the cutoff as a fixed value or asked users to set it themselves, the cutoff should be defined in a more rational way, such as being based on the distribution model of PET lengths. What is more, the available replicates should be rationally used to correct background and enhance the results, such as Hichipper employed a combinatory strategy to merge all the replicates to detect loops, which achieved the highest reproducibility in all HiChIP datasets (Additional file 1: Fig. S11).

The recently developed long-read ChIA-PET [18] protocols provide better resolution and require lower chromatin input than ChIA-PET. The only publicly available long-read ChIA-PET data is from Tang et al. [46] which does not match any available HiChIP conditions. Thus, we were only able to compare long-read ChIA-PET data with standard ChIA-PET data. Within all the ChIA-PET analytical methods, only CPT2 [25] and CPTv.3 [26] supported the raw data analysis of long-read ChIA-PET data; thus, we compared the performance of the latest approach ChIA-PIPE [47] with CPT2 and CPTv.3. The results showed that ChIA-PIPE performed slightly better than CPT2 and CPTv.3 in all the long-read ChIA-PET datasets (Additional file 1: Table S3). Overall, there still lacks more powerful tools suitable for the long-read ChIA-PET protocol.

Finally, we believe that the next generation of computational tools for analyzing protein-directed 3D chromatin topology should boost sensitivity to account for weak or dynamic interactions. Inherited from the processing pipelines of ChIP-based techniques, peak calling was the most frequently used strategy to detect the enrichment of PETs. Since peak calling results rely highly on ChIP enrichment, some weak or dynamic interactions are likely to go undetected [34]. Cluster-based methods rely on the relative read densities rather than the absolute values, making more locally enriched loops standout compared to peak-based methodologies. Thus, cluster-based methods offer a viable solution to detect more dynamic topological interactions [34]. However, this sensitivity comes at the expense accuracy. How to balance the detection of dynamic loops while maintaining the accurate detection of strong loops remains a challenge which needs to be addressed. A rational approach to the development of such an analytical pipeline would be a mixed model, where the accuracy of the top-performing peak-based approaches and the sensitivity of the cluster-based pipelines are combined. A number of considerations have to be made for such a mixed model, but ultimately it needs to limit noise, boost signal, and output an accurate representation of the factor-enriched 3D genome. Firstly, noise has to be reduced. Both ChIAPoP and Hichipper(+chip) reduced noise more effectively than other pipelines. ChIAPoP cleverly incorporates into their model the use of chimeric reads, in addition to sequencing depth and inter-anchor distance, for estimating noise. And the addition of ChIP-seq data to Hichipper also dramatically improves noise reduction. Both strategies incorporated together within a statistical framework could theoretically aid in the noise reduction necessary for the generation of a mixed peak- and cluster-based model.

## Conclusion

Currently, many computational methods and packages are available to analyze HiChIP and ChIA-PET datasets. However, it is challenging to compare the performance of these different computational pipelines without the use of uniform gold standard datasets and evaluation metrics. With this in mind, we developed a comprehensive benchmark framework, Bacon, to evaluate the performance of different computational methods and provide practical recommendations to fit different analysis requirements. We investigated the diverse characteristics of ChIA-PET and HiChIP datasets, and deployed Bacon to benchmark 12 computational methods comprehensively. The evaluation results indicated that ChIAPoP outperformed the others in reliability and accuracy for ChIA-PET analysis, while FitHiChIP-S and Hichipper(+chip) outperformed the other available methods for HiChIP analysis. However, these methods still presented different deficiencies, and no single method stood out in every analytical aspect. Overall, the lessons learned from our evaluation of these analytical tools can be leveraged to improve the design of future computational pipelines.

## Methods

### Calculation of Uniquely mapped Valid Rate (UV Rate)

Different linker trimming strategies and alignment tools were used by different ChIA-PET and HiChIP analytical methods to pre-process the raw reads. In the pre-processing step of Bacon, we tried 0 and 1 mismatch for ChIAPoP and ChIA-PET2. And for ChIA-PETv.3, we used the default linker alignment score to trim linkers. For alignment, the minimum mapping rate (MAPQ) was set 30, and the duplicates were filtered by Picard. The Uniquely mapped Valid (UV) PETs were retained, and UV Rate was defined as the number of UV PETs divided by the number of total PETs. Although we set the same filtering threshold for different methods, the different fixed settings specific to each method impacted the UV Rate.

### Gathering of gold standard loop sets

The candidate long-range interactions of K562-POLR2A, MCF7-ESR1, K562-H3K27ac, K562-H3K4me1, K562-H3K4me2, and K562-H3K4me3 were downloaded from ENCODE (ENCSR000BZY, ENCSR000BZZ, ENCSR000FDG, ENCSR000FDD, ENCSR000FDE, ENCSR000FDF) [41], and the candidate long-range interactions of mESC-Smc1, mESC-YY1, K562-CTCF, K562-YY1, HeLa-S3-CTCF, PAEC-LSS, PAEC-ST, mESC-FGFR1, erythroblast-Gata1, mESC-Klf4, BMDM-H3K4me3, mESC-H3K27me3, AML12-H3K9me3 were downloaded from GEO under accession number of GSE59395 [48], GSE57911 [49], GSE39495 [41], GSE99519 [50], GSE137849 [51], GSE153013 [52], GSE153013 [52], GSE153884 [53], GSE112717 [54], GSE113339 [55], GSE159629 [56], GSE160656 [57], and GSE141113 [58].

The GEUVADIS eQTL data was downloaded from ftp://ftp.ebi.ac.uk/pub/databases/microarray/data/ex periment/GEUV/E-GEUV-1/analysis_results/. The GTEx eQTL data of transformed lymphocytes was downloaded from https://www.gtexportal.org/home/datasets. For the variants and gene pairs of eQTLs data, we extracted the genomic loci of variants then extended to 5 kb length from both ends and extracted the genomic coordinates of genes from GENCODE v19 annotation. The verified Enhancer-gene pairs in K562 with CRISPR/Cas9 perturbations were downloaded from the study of Gasperini et al. [40].

Then we intersected the verified contacts (eQTLs and CRISPR/Cas9 perturbations pairs) with the candidate loops downloaded from ENCODE and GEO and counted the coverage for both anchors of candidate loops, and the gold standard loop sets were constructed by selected the candidate loops covered by verified contacts significantly (*p* value< 0.05 and FDR < 0.05). For mESC-Smc1, mESC-Yy1, mESC-Fgfr1, erythroblast-Gata1, mESC-Klf4, BMDM-H3K4me3, mESC-H3K27me3, and AML12-H3K9me3, no publicly available eQTLs or CRISPR/Cas9 validated data existed for the calling of gold standard loops. To generate gold standard murine loops, we used the ChIP-seq peaks of corresponding cell line and antibody (GSE22562 [59], GSE31785 [60], GSE65698 [61], GSE112717 [54], GSE113429 [55], GSE23619 [62], GSE162739 [63], GSE143886 [64]) to count the coverage for both anchors, then calculated the significance.

### Generation of Hi-C loops with strong signals

For the cell lines of K562, GM12878, mESC, MCF7, HeLa-S3, and AML12, the compressed binary (.hic) files were downloaded from https://bcm.app.box.com/v/aidenlab/folder/11234760671, and GSE134621 [64]. Then HiCCUPS [31] was used to call loops for .hic files with the resolution of 1 Mb, and KR (Knight-Ruiz) balancing mode was used to normalize the data, the loops with strong signals were defined as FDR ≤ 0.01 and observed counts ≥ 5. For BMDMs, erythroblast, and PAEC, the Hi-C interactions were downloaded from GEO under accession number of GSE109965 [65], GSE168176 [66], and GSE152900, then we filtered the interactions with a threshold of 5.

### Calculation of accuracy (ACC)

To evaluate the accuracy of different computational methods, we regarded gold standard loops as “true” loops, the “false” loops were constructed with the same number as “true” loops. We firstly constructed the potential false loops set by selecting the genomic location avoiding TSS regions of genes and enhancer-like regions from ENCODE, which excluded all potential enhancer-promoter loops. Then we excluded the eQTL containing loops, CRISPR/Cas9 validated loops, and strong Hi-C loop signals from the potential false loops set. Since the number of potential false loops was much greater than the “true” loops, we then randomly selected the same number of false loops from the potential loop set. To eliminate the effect of randomness, we repeated the selection three times, thus we had 3 “false loops” files for each true loops file. Then we defined true positive (TP) as the number of detected loops which intersected with gold standard loops, false positive (FP) as the number of detected loops intersected with “false” loops, true negative (TN) as the number of “false” loops NOT intersected with detected loops, and false negative (FN) as the number of gold standard loops NOT intersected with detected loops. Since there were three “false” loop files for each true loop file, we calculated the mean of three FPs as the final FP value to calculate accuracy. The accuracy (ACC) was calculated as follows:

$$ \mathrm{ACC}=\frac{\mathrm{TP}+\mathrm{TN}}{\mathrm{TP}+\mathrm{TN}+\mathrm{FP}+\mathrm{FN}} $$

### Simulation of ChIA-PET and HiChIP data

To test the performance of computational methods in the simulation datasets with different ChIP antibodies, we generated anchor pairs for ChIA-PET and HiChIP, according to the different data characteristics between these two types of data. For ChIA-PET anchor pairs, a Poisson model was used as described previously [22]. Firstly, the chromosomes were segmented into bins of 5000 bp, the loci of segmented points were recorded and used to construct a potential contact pairs matrix, then we simulated the interaction between genomic loci *i* and *j* as *n*_*ij*_~*Pois*(*λ*_*ij*_), the expectation ${\lambda}_{ij}=\frac{a}{1+\delta }$, with *δ* =  ∣ *i* − *j*∣, *a* represented the number of anchors used for simulation, and the interaction frequencies depend on the genomic distance strongly. The HiChIP anchor pairs were generated with Hi-C data as described by Bhattacharyya [30]. Firstly, we set the bin size (5000 bp) to extract Hi-C pairs as HiChIP anchor pairs. Secondly, the published ChIP-seq data publicly available for each antibody was used to simulate the ChIP enrichment, we then calculated ChIP-seq read coverage for the generated anchor pairs. Next, we set the coverage threshold as 50% to filter the final simulated valid pairs. Overall, we used three different ChIP antibodies to simulate the ChIA-PET and HiChIP data. Finally, we then tested the performances of different methods with these simulated datasets.

### Processing of ChIA-PET, HiChIP and ChIP-seq peaks

We gathered 10 ChIA-PET and 12 HiChIP raw datasets (for the accession number, see https://csuligroup.com/Bacon), and the public ChIP-seq data were derived from NCBI with number GSE22562 [59], GSE31785 [60], GSE65698 [61], GSE112717 [54], GSE113429 [55], GSE23619 [62], GSE162739 [63], and GSE143886 [64]. The raw reads were aligned to mm10 or hg19 reference genome by bowtie2 [67] with default parameters. For track visualizing, the uniquely mapped reads were fed into the “bamCoverage” function of deeptools [68] with “–binSize 10 –normalizeUsing RPGC –effectiveGenomeSize 2150570000” to convert the bam alignment into bigwig. For detecting peak regions, MACS2 [32] was utilized to call peaks with “-q 0.01 -B –SPMR –keep-dup all.” Then the peaks from different datasets were merged by Bedtools with at least 80% length overlap, and we counted reads from all the datasets over these peaks individually.

### Calculation of peak co-occupancy (PC)

The uniquely mapped alignment files of ChIA-PET and HiChIP were obtained in the pre-processing step of Bacon, which were used to call loops by different computational methods. Bacon extracted the anchors from called loops, then the anchors with a minimum 90% of length overlapped with each other were merged. The public ChIP-seq peak was used as target set to detect overlaps with different anchor sets, all the anchors that had at least 1 bp overlap with the ChIP-seq peaks were gathered into a candidate set *A*, the total number of anchors in loop set was represented by *N*, and the number of anchors in *A* was represented by *N*_*A*_. The length of anchor *i* was represented by *L*(*a*_*i*_).The peak overlapped with anchor *i was P*_*i*_, and the length of this peak was *L*(*p*_*i*_), overlapping length between anchor *i* and ChIP-seq peak was represented by *L*(*o*_*i*_). If there were more than one peak overlapped with the anchor, the peak with longest overlapping length was selected. The PC was calculated as follows:

$$ PC=\frac{N_A\ast {\sum}_{i=1}^N\mathit{\max}\left\{\frac{L\left({o}_i\right)}{L\left({p}_i\right)},\frac{L\left({o}_i\right)}{L\left({a}_i\right)}\right\}}{N} $$

### PCA and differential analysis for ChIP-seq, ChIA-PET and HiChIP

Given that ChIP-seq, HiChIP, and ChIA-PET all rely on chromatin immunoprecipitation, they should display similar binding profiles when comparisons are being made for a single protein of interest. We implemented DiffBind [69] to perform PCA analysis and to give a deeper insight into how these experimental groups were associated. The alignment files derived from ChIP-seq, ChIA-PET, and HiChIP replicates were filtered using Samtools [70] with minimum MAPQ 30. Picard was then used to remove duplicates, and the filtered bam files were prepared as the input to function “dba,” then a binding matrix with scores was calculated based on read counts for every sample with function “dba.count.” The data were normalized based on sequencing depth with default setting of function “dba.normalize,” then the function “dba.plotPCA” was used to see how well the samples cluster with one another.

Before running the differential analysis, we used “DownsampleSam” function of Picard with “*P* = 0.2” to downsample the alignment files. “dba.contrast” function with default mode to model the data, as well as specify the comparisons we are interested in like ChIP-seq vs ChIA-PET and ChIP-seq vs HiChIP. Then “dba.analyze” function was used to perform the differential analysis, and the *p* value < 0.05 and log2(fold change) > 1 was used as the threshold of significance to detect differential peaks for one of the antibody. We repeated the differential analysis for ChIP-seq vs ChIA-PET and ChIP-seq vs HiChIP on every antibody, then all the differential peaks were aggregated.

### Calculation of enrichment score (ES)

ES was used to evaluate the enrichment level of loops identified by the cluster-based methods. Bacon defined ES on the assumption that the true enriched loci should be surrounded by relative low enriched PETs, which means the enrichment of anchor location was the local maximum. The genomic coordinates of two anchors were represented by *s1*, *e1*, *s2*, and *e2*, and Bacon firstly calculated the average length of two anchors, ${l}_m=\frac{l_a+{l}_b}{2}$, in which *l*_*a*_ = *e*1 − *s*1, *l*_*b*_ = *e*2 − *s*2. *P*_*x*_ was defined as the number of PETs in genomic region *x*. The enriched number of PETs within the neighbor region of loop *i* was represented by ${P}_n^i$.

$$ {P}_n^i=\mathit{\operatorname{Min}}\left({P}_{s1-{l}_m}^i+{P}_{e1+{l}_m}^i,{P}_{s2-{l}_m}^i+{P}_{e2+{l}_m}^i\right) $$

The PET count of loop *i* was defined as *C*^*i*^, and ES of loop *i* was calculated as follows:

$$ {ES}_i=\frac{C^i}{P_n^i} $$

If the value of *ES*_*i*_ less than 1, indicating the enrichment of neighbor region was higher than the enrichment of loop anchor, loop *i* was thought to be an invalid loop. Thus, the higher the *ES*_*i*_, the more reliable the loop.

To estimate the enrichment level of all the loops in a genome-wide fashion, Bacon calculated the global ES,

$$ {ES}_G=\frac{\sum_{i=1}^N{C}^i}{\sum_{i=1}^N{P}_n^i}\ast \propto $$

in which, ∝ is a coefficient to adjust the enrichment of low PET coverage regions caused by uneven sequencing depth. The PET coverage of whole genome was calculated by C = *LN*/*G*, *C* is for coverage, *G* is the length of genome, *L* is the length of PET, *N* is the number of PETs, *C*_*j*_ is the PET coverage of *j* chromosome, and ∝ is calculated by $\propto =\frac{C}{C_j}$ .

### Estimating resolution level of loops

We firstly calculated the distance between two anchor regions, and the loops were segregated into three types according to the range of distance *d*: *d ≤* 10 kb, 10 kb < *d ≤* 100 kb, and 100 kb < *d* ≤ 1 Mb. The number of loops in each type divided by the total number of loops was the resolution level in this range, and the resolution level was then plotted as heatmap.

### Activation rate (AR) of significant loops

The significant loops were firstly filtered with at least 3 PETs of ChIA-PET data, at least 8 PETs of HiChIP data, and the *p* value threshold was set as 0.05, false discovery rate (FDR) threshold was 0.05 if accessible for the method. The candidate enhancer-like and promoter-like signatures files were downloaded from ENCODE, and the anchors of filtered loops were extracted to overlay with the enhancer-like and promoter-like elements, then the percentages of E-E/E-P/P-P loops were counted. Then the ChIP-seq peaks of active histone markers (H3K27ac, H3K4me1, H3K4me3), ATAC-seq peaks, and repressive histone marker (H3K27me3) were collected to overlap with the E-E/E-P/P-P loops. The overlapping length between anchors and peaks were calculated by Bedtools [71]; if the overlapping length of activate peaks is larger than that of inactive peaks, the loop was thought to be active. Otherwise, the loop was thought to be inactive. If there is no active or repressive peak overlying, or the active overlapping length is equal to the inactive overlapping length, the loop was classified to other type. The percentage of active loops was defined as activation rate (AR). The annotation of loops and the calculation of AR were implemented by homemade scripts (see Bacon webpage).

## Supplementary Information

**Additional file 1: Tables S1-S5** and **Figures S1-S11.**

**Additional file 2: Table S6.** Statistics of experimental datasets and simulation datasets.

**Additional file 3: Table S7.** Evaluation results of ACC in ChIA-PET datasets.

**Additional file 4: Table S8.** Evaluation results of ACC in HiChIP datasets.

**Additional file 5.** Review history.

## Data Availability

The datasets analyzed during the current study are available in the NCBI repository with the GEO accession number of GSE57911 [49], GSE101498 [42, 72], GSE59395 [48], GSE51334 [73], GSE99173 [74], GSE141525 [75], GSE90994 [76, 77], GSE22562 [59], GSE31785 [60], GSE65698 [61], GSE112717 [54], GSE113429 [55], GSE23619 [62], GSE162739 [63], GSE14388 6[64], GSE39495 [41], GSE99519 [50], GSE137849 [51], GSE153013 [52], GSE153884 [53], GSE113339 [55], GSE159629 [56], GSE160656 [57], and GSE141113 [58]. And from the ENCODE project [41] with accession number of ENCSR000CAC, ENCSR597AKG, ENCSR752QCX, ENCSR000AKP, ENCSR000BZY, ENCSR000BZZ, ENCSR000FDG, ENCSR000FDD, ENCSR000FDE, and ENCSR000FDF. All the processed data files, final results, and computational pipeline of Bacon are freely available under GNU General Public License v3.0 from https://csuligroup.com/Bacon and https://github.com/CSUBioGroup/Bacon [78]. The code is also achieved at Zenodo with DOI 10.5281/zenodo.5607035 [79].

## References

[1] Davies JOJ, Oudelaar AM, Higgs DR, Hughes JR (2017). How best to identify chromosomal interactions: a comparison of approaches. Nat Methods..

[2] Bulger M, Groudine M (2011). Functional and mechanistic diversity of distal transcription enhancers. Cell..

[3] Stamatoyannopoulos J (2016). Connecting the regulatory genome. Nat Genet..

[4] Kooren J, Simonis M, de Laat W (2007). An evaluation of 3C-based methods to capture DNA interactions. Nat Methods..

[5] Hagège H, Klous P, Braem C, Splinter E, Dekker J, Cathala G (2007). Quantitative analysis of chromosome conformation capture assays (3C-qPCR). Nat Protoc.

[6] Denker A, de Laat W (2016). The second decade of 3C technologies: detailed insights into nuclear organization. Gene Dev..

[7] van de Werken HJG, de Vree PJP, Splinter E, Holwerda SJB, Klous P, de Wit E (2012). Chapter Four 4C Technology: protocols and data analysis. Methods Enzymol..

[8] van de Werken HJG, Landan G, Holwerda SJB, Hoichman M, Klous P, Chachik R, Splinter E, Valdes-Quezada C, Öz Y, Bouwman BAM, Verstegen MJAM, de Wit E, Tanay A, de Laat W (2012). Robust 4C-seq data analysis to screen for regulatory DNA interactions. Nat Methods..

[9] Ferraiuolo MA, Sanyal A, Naumova N, Dekker J, Dostie J (2012). From cells to chromatin: Capturing snapshots of genome organization with 5C technology. Methods..

[10] Lieberman-Aiden E, van Berkum NL, Williams L, Imakaev M, Ragoczy T, Telling A, Amit I, Lajoie BR, Sabo PJ, Dorschner MO, Sandstrom R, Bernstein B, Bender MA, Groudine M, Gnirke A, Stamatoyannopoulos J, Mirny LA, Lander ES, Dekker J (2009). Comprehensive mapping of long-range interactions reveals folding principles of the human genome. Science..

[11] Dixon JR, Selvaraj S, Yue F, Kim A, Li Y, Shen Y, Hu M, Liu JS, Ren B (2012). Topological domains in mammalian genomes identified by analysis of chromatin interactions. Nature..

[12] Nora EP, Lajoie BR, Schulz EG, Giorgetti L, Okamoto I, Servant N, Piolot T, van Berkum NL, Meisig J, Sedat J, Gribnau J, Barillot E, Blüthgen N, Dekker J, Heard E (2012). Spatial partitioning of the regulatory landscape of the X-inactivation centre. Nature..

[13] Tolhuis B, Palstra R-J, Splinter E, Grosveld F, de Laat W (2002). Looping and interaction between hypersensitive sites in the active β-globin locus. Mol Cell..

[14] Hsieh T-HS, Fudenberg G, Goloborodko A, Rando OJ (2016). Micro-C XL: assaying chromosome conformation from the nucleosome to the entire genome. Nat Methods..

[15] Hsieh T-HS, Weiner A, Lajoie B, Dekker J, Friedman N, Rando OJ (2015). Mapping nucleosome resolution chromosome folding in yeast by Micro-C. Cell..

[16] Tan-Wong SM, Zaugg JB, Camblong J, Xu Z, Zhang DW, Mischo HE, Ansari AZ, Luscombe NM, Steinmetz LM, Proudfoot NJ (2012). Gene loops enhance transcriptional directionality. Science..

[17] Fullwood MJ, Liu MH, Pan YF, Liu J, Xu H, Mohamed YB, Orlov YL, Velkov S, Ho A, Mei PH, Chew EGY, Huang PYH, Welboren WJ, Han Y, Ooi HS, Ariyaratne PN, Vega VB, Luo Y, Tan PY, Choy PY, Wansa KDSA, Zhao B, Lim KS, Leow SC, Yow JS, Joseph R, Li H, Desai KV, Thomsen JS, Lee YK, Karuturi RKM, Herve T, Bourque G, Stunnenberg HG, Ruan X, Cacheux-Rataboul V, Sung WK, Liu ET, Wei CL, Cheung E, Ruan Y (2009). An oestrogen-receptor-α-bound human chromatin interactome. Nature..

[18] Li X, Luo OJ, Wang P, Zheng M, Wang D, Piecuch E, Zhu JJ, Tian SZ, Tang Z, Li G, Ruan Y (2017). Long-read ChIA-PET for base-pair-resolution mapping of haplotype-specific chromatin interactions. Nat Protoc..

[19] Mumbach MR, Rubin AJ, Flynn RA, Dai C, Khavari PA, Greenleaf WJ, Chang HY (2016). HiChIP: efficient and sensitive analysis of protein-directed genome architecture. Nat Methods..

[20] Fang R, Yu M, Li G, Chee S, Liu T, Schmitt AD, Ren B (2016). Mapping of long-range chromatin interactions by proximity ligation-assisted ChIP-seq. Cell Res..

[21] Li G, Fullwood MJ, Xu H, Mulawadi FH, Velkov S, Vega V, Ariyaratne PN, Mohamed YB, Ooi HS, Tennakoon C, Wei CL, Ruan Y, Sung WK (2010). ChIA-PET tool for comprehensive chromatin interaction analysis with paired-end tag sequencing. Genome Biol..

[22] Paulsen J, Rødland EA, Holden L, Holden M, Hovig E (2014). A statistical model of ChIA-PET data for accurate detection of chromatin 3D interactions. Nucleic Acids Res..

[23] He C, Zhang MQ, Wang X (2015). MICC: an R package for identifying chromatin interactions from ChIA-PET data. Bioinformatics..

[24] Phanstiel DH, Boyle AP, Heidari N, Snyder MP (2015). Mango: a bias-correcting ChIA-PET analysis pipeline. Bioinformatics..

[25] Li G, Chen Y, Snyder MP, Zhang MQ (2017). ChIA-PET2: a versatile and flexible pipeline for ChIA-PET data analysis. Nucleic Acids Res..

[26] Li G, Sun T, Chang H, Cai L, Hong P, Zhou Q (2019). Chromatin interaction analysis with updated ChIA-PET tool (V3). Genes-basel..

[27] Huang W, Medvedovic M, Zhang J, Niu L (2019). ChIAPoP: a new tool for ChIA-PET data analysis. Nucleic Acids Res.

[28] Lareau CA, Aryee MJ (2018). hichipper: a preprocessing pipeline for calling DNA loops from HiChIP data. Nat Methods.

[29] Juric I, Yu M, Abnousi A, Raviram R, Fang R, Zhao Y, Zhang Y, Qiu Y, Yang Y, Li Y, Ren B, Hu M (2019). MAPS: Model-based analysis of long-range chromatin interactions from PLAC-seq and HiChIP experiments. Plos Comput Biol..

[30] Bhattacharyya S, Chandra V, Vijayanand P, Ay F (2019). Identification of significant chromatin contacts from HiChIP data by FitHiChIP. Nat Commun..

[31] Durand NC, Shamim MS, Machol I, Rao SSP, Huntley MH, Lander ES, Aiden EL (2016). Juicer provides a one-click system for analyzing loop-resolution Hi-C experiments. Cell Syst..

[32] Zhang Y, Liu T, Meyer CA, Eeckhoute J, Johnson DS, Bernstein BE, Nusbaum C, Myers RM, Brown M, Li W, Liu XS (2008). Model-based analysis of ChIP-Seq (MACS). Genome Biol..

[33] Cao Y, Chen Z, Chen X, Ai D, Chen G, McDermott J, Huang Y, Guo X, Han JDJ (2019). Accurate loop calling for 3D genomic data with cLoops. Bioinformatics..

[34] Guo Y, Krismer K, Closser M, Wichterle H, Gifford DK (2019). High resolution discovery of chromatin interactions. Nucleic Acids Res.

[35] Forcato M, Nicoletti C, Pal K, Livi CM, Ferrari F, Bicciato S (2017). Comparison of computational methods for Hi-C data analysis. Nat Methods..

[36] Yardımcı GG, Ozadam H, Sauria MEG, Ursu O, Yan K-K, Yang T, Chakraborty A, Kaul A, Lajoie BR, Song F, Zhan Y, Ay F, Gerstein M, Kundaje A, Li Q, Taylor J, Yue F, Dekker J, Noble WS (2019). Measuring the reproducibility and quality of Hi-C data. Genome Biol..

[37] Zufferey M, Tavernari D, Oricchio E, Ciriello G (2018). Comparison of computational methods for the identification of topologically associating domains. Genome Biol..

[38] Consortium TG, Lappalainen T, Sammeth M, Friedländer MR, PAC’t H, Monlong J (2013). Transcriptome and genome sequencing uncovers functional variation in humans. Nature..

[39] Aguet F, Brown A, Castel SE, Davis JR, He Y, Jo B (2017). Genetic effects on gene expression across human tissues. Nature..

[40] Gasperini M, Hill AJ, McFaline-Figueroa JL, Martin B, Kim S, Zhang MD (2019). A genome-wide framework for mapping gene regulation via cellular genetic screens. Cell.

[41] Consortium TEP (2012). An integrated encyclopedia of DNA elements in the human genome. Nature..

[42] Mumbach MR, Satpathy AT, Boyle EA, Dai C, Gowen BG, Cho SW (2017). Enhancer connectome in primary human cells identifies target genes of disease-associated DNA elements. Nat Genet.

[43] Fulco CP, Munschauer M, Anyoha R, Munson G, Grossman SR, Perez EM, Kane M, Cleary B, Lander ES, Engreitz JM (2016). Systematic mapping of functional enhancer–promoter connections with CRISPR interference. Science..

[44] Nagano T, Lubling Y, Stevens TJ, Schoenfelder S, Yaffe E, Dean W, Laue ED, Tanay A, Fraser P (2013). Single-cell Hi-C reveals cell-to-cell variability in chromosome structure. Nature..

[45] Kind J, van Steensel B (2014). Stochastic genome-nuclear lamina interactions: modulating roles of Lamin A and BAF. Nucl Austin Tex..

[46] Tang Z, Luo OJ, Li X, Zheng M, Zhu JJ, Szalaj P, Trzaskoma P, Magalska A, Wlodarczyk J, Ruszczycki B, Michalski P, Piecuch E, Wang P, Wang D, Tian SZ, Penrad-Mobayed M, Sachs LM, Ruan X, Wei CL, Liu ET, Wilczynski GM, Plewczynski D, Li G, Ruan Y (2015). CTCF-mediated human 3D genome architecture reveals chromatin topology for transcription. Cell..

[47] Lee B, Wang J, Cai L, Kim M, Namburi S, Tjong H (2020). ChIA-PIPE: a fully automated pipeline for comprehensive ChIA-PET data analysis and visualization. Sci Adv.

[48] Heidari N, Phanstiel DH, He C, Grubert F, Jahanbani F, Kasowski M, Zhang MQ, Snyder MP (2014). Genome-wide map of regulatory interactions in the human genome. Genome Res..

[49] Dowen JM, Fan ZP, Hnisz D, Ren G, Abraham BJ, Zhang LN, Weintraub AS, Schuijers J, Lee TI, Zhao K, Young RA (2014). Control of cell identity genes occurs in insulated neighborhoods in mammalian chromosomes. Cell..

[50] Weintraub AS, Li CH, Zamudio AV, Sigova AA, Hannett NM, Day DS (2017). YY1 is a structural regulator of enhancer-promoter loops. Cell.

[51] Hu G, Dong X, Gong S, Song Y, Hutchins AP, Yao H (2020). Systematic screening of CTCF binding partners identifies that BHLHE40 regulates CTCF genome-wide distribution and long-range chromatin interactions. Nucleic Acids Res.

[52] Moonen J-RAJ, Chappell J, Shi M, Shinohara T, Li D, Mumbach MR, et al. KLF4 recruits SWI/SNF to increase chromatin accessibility and reprogram the endothelial enhancer landscape under laminar shear stress. Biorxiv. 2020:2020.07.10.195768. 10.1038/s41467-022-32566-9 PMC9399231 35999210

[53] Decker B, Liput M, Abdellatif H, Yergeau D, Bae Y, Jornet JM, Stachowiak EK, Stachowiak MK (2020). Global genome conformational programming during neuronal development is associated with CTCF and nuclear FGFR1—The Genome Archipelago Model. Int J Mol Sci..

[54] Cai W, Huang J, Zhu Q, Li BE, Seruggia D, Zhou P, Nguyen M, Fujiwara Y, Xie H, Yang Z, Hong D, Ren P, Xu J, Pu WT, Yuan GC, Orkin SH (2020). Enhancer dependence of cell-type–specific gene expression increases with developmental age. Proc National Acad Sci..

[55] Giammartino DCD, Kloetgen A, Polyzos A, Liu Y, Kim D, Murphy D, et al. KLF4 binding is involved in the organization and regulation of 3D enhancer networks during acquisition and maintenance of pluripotency. Biorxiv. 2019;382473.

[56] Hoeksema MA, Shen Z, Holtman IR, Zheng A, Spann NJ, Cobo I (2021). Mechanisms underlying divergent responses of genetically distinct macrophages to IL-4. Sci Adv.

[57] Crispatzu G, Rehimi R, Pachano T, Bleckwehl T, Cruz-Molina S, Xiao C, Mahabir E, Bazzi H, Rada-Iglesias A (2021). The chromatin, topological and regulatory properties of pluripotency-associated poised enhancers are conserved in vivo. Nat Commun..

[58] Huo X, Ji L, Zhang Y, Lv P, Cao X, Wang Q (2020). The nuclear matrix protein SAFB cooperates with major satellite RNAs to stabilize heterochromatin architecture partially through phase separation. Mol Cell.

[59] Kagey MH, Newman JJ, Bilodeau S, Zhan Y, Orlando DA, van Berkum NL, Ebmeier CC, Goossens J, Rahl PB, Levine SS, Taatjes DJ, Dekker J, Young RA (2010). Mediator and cohesin connect gene expression and chromatin architecture. Nature..

[60] Vella P, Barozzi I, Cuomo A, Bonaldi T, Pasini D (2012). Yin Yang 1 extends the Myc-related transcription factors network in embryonic stem cells. Nucleic Acids Res..

[61] Terranova C, Narla ST, Lee Y-W, Bard J, Parikh A, Stachowiak EK, Tzanakakis ES, Buck MJ, Birkaya B, Stachowiak MK (2015). Global developmental gene programing involves a nuclear form of fibroblast growth factor receptor-1 (FGFR1). Plos One..

[62] Escoubet-Lozach L, Benner C, Kaikkonen MU, Lozach J, Heinz S, Spann NJ, Crotti A, Stender J, Ghisletti S, Reichart D, Cheng CS, Luna R, Ludka C, Sasik R, Garcia-Bassets I, Hoffmann A, Subramaniam S, Hardiman G, Rosenfeld MG, Glass CK (2011). Mechanisms establishing TLR4-responsive activation states of inflammatory response genes. Plos Genet..

[63] Conway E, Rossi F, Tamburri S, Ponzo E, Ferrari KJ, Zanotti M, et al. BAP1 activity regulates PcG occupancy and global chromatin condensation counteracting diffuse PCGF3/5-dependent H2AK119ub1 deposition. Biorxiv. 2020:2020.12.10.419309.

[64] Ji L, Huo X, Zhang Y, Yan Z, Wang Q, Wen B (1863). TOPORS, a tumor suppressor protein, contributes to the maintenance of higher-order chromatin architecture. Biochimica Et Biophysica Acta Bba - Gene Regul Mech..

[65] Link VM, Duttke SH, Chun HB, Holtman IR, Westin E, Hoeksema MA (2018). Analysis of genetically diverse macrophages reveals local and domain-wide mechanisms that control transcription factor binding and function. Cell.

[66] Zhang H, Lam J, Zhang D, Lan Y, Vermunt MW, Keller CA, Giardine B, Hardison RC, Blobel GA (2021). CTCF and transcription influence chromatin structure re-configuration after mitosis. Nat Commun..

[67] Langmead B, Salzberg SL (2012). Fast gapped-read alignment with Bowtie 2. Nat Methods..

[68] Ramírez F, Ryan DP, Grüning B, Bhardwaj V, Kilpert F, Richter AS, Heyne S, Dündar F, Manke T (2016). deepTools2: a next generation web server for deep-sequencing data analysis. Nucleic Acids Res..

[69] Ross-Innes CS, Stark R, Teschendorff AE, Holmes KA, Ali HR, Dunning MJ, Brown GD, Gojis O, Ellis IO, Green AR, Ali S, Chin SF, Palmieri C, Caldas C, Carroll JS (2012). Differential oestrogen receptor binding is associated with clinical outcome in breast cancer. Nature..

[70] Danecek P, Bonfield JK, Liddle J, Marshall J, Ohan V, Pollard MO (2021). Twelve years of SAMtools and BCFtools. Gigascience.

[71] Quinlan AR, Hall IM (2010). BEDTools: a flexible suite of utilities for comparing genomic features. Bioinformatics..

[72] Rubin AJ, Parker KR, Satpathy AT, Qi Y, Wu B, Ong AJ (2019). Coupled single-cell CRISPR screening and epigenomic profiling reveals causal gene regulatory networks. Cell.

[73] Pope BD, Ryba T, Dileep V, Yue F, Wu W, Denas O, Vera DL, Wang Y, Hansen RS, Canfield TK, Thurman RE, Cheng Y, Gülsoy G, Dennis JH, Snyder MP, Stamatoyannopoulos JA, Taylor J, Hardison RC, Kahveci T, Ren B, Gilbert DM (2014). Topologically associating domains are stable units of replication-timing regulation. Nature..

[74] Liu X, Zhang Y, Chen Y, Li M, Zhou F, Li K (2017). In situ capture of chromatin interactions by biotinylated dCas9. Cell.

[75] Zhang T, Zhang Z, Dong Q, Xiong J, Zhu B (2020). Histone H3K27 acetylation is dispensable for enhancer activity in mouse embryonic stem cells. Genome Biol..

[76] Hansen AS, Pustova I, Cattoglio C, Tjian R, Darzacq X (2017). CTCF and cohesin regulate chromatin loop stability with distinct dynamics. Elife..

[77] Cattoglio C, Pustova I, Walther N, Ho JJ, Hantsche-Grininger M, Inouye CJ, Hossain MJ, Dailey GM, Ellenberg J, Darzacq X, Tjian R, Hansen AS (2019). Determining cellular CTCF and cohesin abundances to constrain 3D genome models. Elife..

[78] Tang L, Hill MC, Ellinor PT, Li M: Bacon: a comprehensive computational benchmarking framework for evaluating targeted chromatin conformation capture-specific methodologies. Github https://github.com/CSUBioGroup/Bacon 2021. 10.1186/s13059-021-02597-4 PMC8780810 35063001

[79] Tang L, Hill MC, Ellinor PT, Li M: Bacon: a comprehensive computational benchmarking framework for evaluating targeted chromatin conformation capture-specific methodologies (Version 1.0). Zenodo 10.5281/zenodo.5607035 2021. 10.1186/s13059-021-02597-4 PMC8780810 35063001

