# Supplementary material for: Bacon: a comprehensive computational benchmarking framework for evaluating targeted chromatin conformation capture-specific methodologies
Source: Genome Biol. 2022 Jan 21;23:30. doi: 10.1186/s13059-021-02597-4 (PMC8780810; doi:10.1186/s13059-021-02597-4)
Supplement: Supplementary file 1 — Additional file 1: Tables S1-S5 and Figures S1-S11. [file 13059_2021_2597_MOESM1_ESM.docx]

**Supplemental Materials**

**Bacon: A comprehensive computational benchmarking framework for evaluating targeted chromatin conformation capture-specific methodologies**

Li Tang^1^, Matthew C. Hill^2,3^, Patrick T. Ellinor^2,3^, Min Li^1*^

1. **Comparison of data characteristics**
2. **Procedure of processing targeted conformation capture data**
3. **Comparison of methods popularity**
4. **Comparison of pre-processing step of different methods**
5. **Comparison of all HiChIP PETs and short-range PETs**
6. **Comparison of cluster-based methods**
7. **Comparison of different loops with peak intensity and histone marks**
8. **Evaluating the accuracy of loops**
9. **Comparison of original and long read ChIA-PET protocols**
10. **Comparison of reproducibility**
11. **Comparison of running time**
12. **Comparison of data characteristics**

We found that mESC-Smc1 HiChIP data was heavily impacted by restriction enzyme treatment, as the read distribution of mESC-Smc1 HiChIP presented restriction enzyme cut site bias. Correspondingly, mESC-Smc1 HiChIP reads were sparse in positions without Mbol restriction sites (Figure. **S1**).


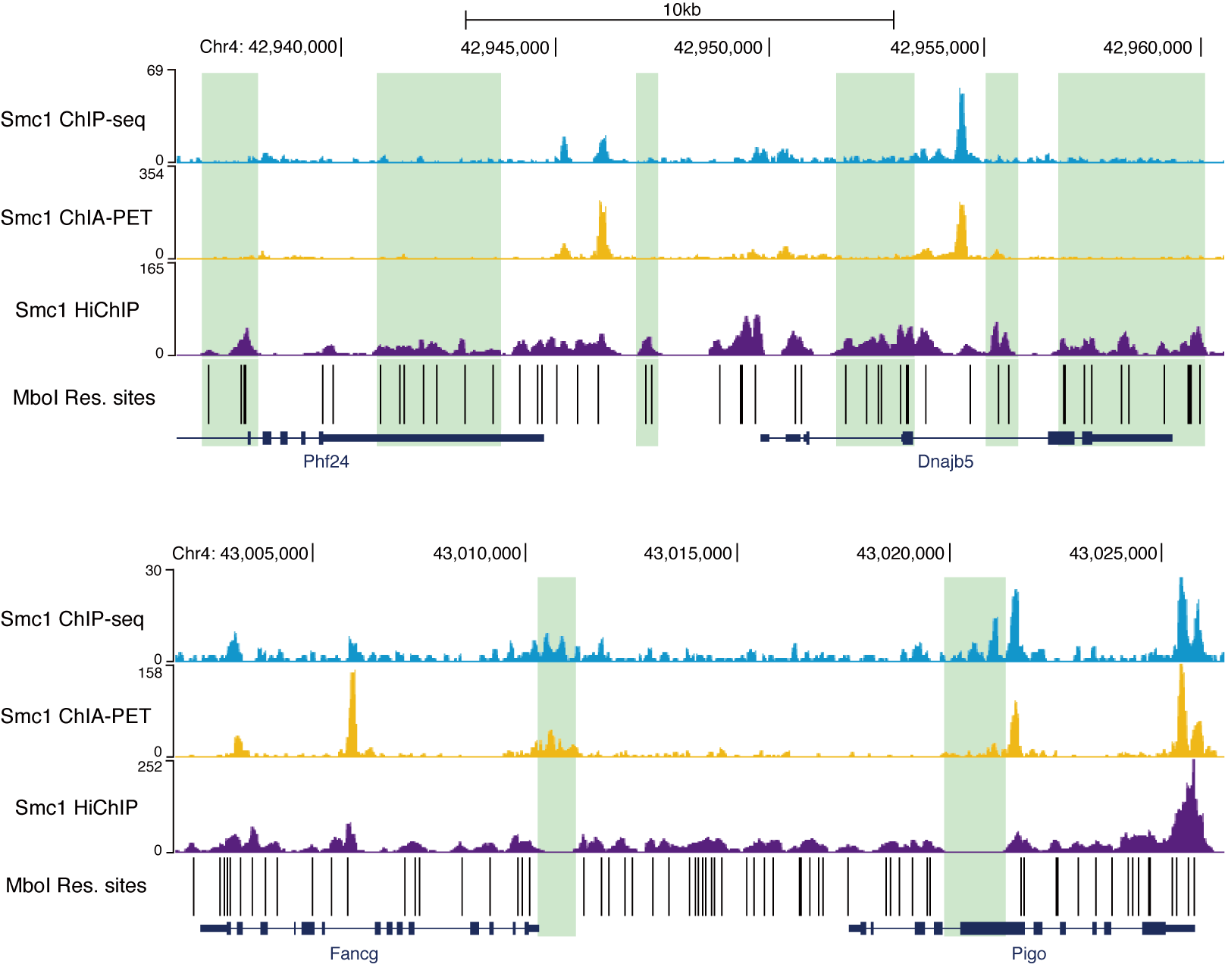


**Figure S1.** Genome browser tracks of Smc1 ChIP-seq, ChIA-PET and HiChIP in mESC. The green bars of upper plot indicate the HiChIP reads which overlap with Mbol restriction sites, when no ChIP-seq/ChIA-PET reads are enriched. Green bars on the lower track indicate HiChIP reads that are sparse in positions without Mbol restriction sites, when there are ChIP-seq/ChIA-PET reads enriched

1. **Procedure of processing targeted conformation capture data**

Chromatin interaction analysis by paired-end tag sequencing (ChIA-PET) is a technique which combines ChIP, 3C and next-generation sequencing to profile long-range contacts bound by a transcription factor or chromatin mark of interest. The recently developed HiChIP and PLAC-seq protocols reveal better resolution and require lower chromatin input than ChIA-PET. The procedure for processing these targeted datasets requires three major steps: pre-processing, calling loops, and the calculation of loop significance (**Fig. S2**). During pre-processing, the trimmed paired-end tags (PETs) are aligned to a reference genome, then the mapped PETs are divided into valid and invalid pairs. For ChIA-PET data, only valid PETs are used for subsequent analysis, while the invalid HiChIP PETs are usually used to measure the ChIP enrichment level for later bias correction.


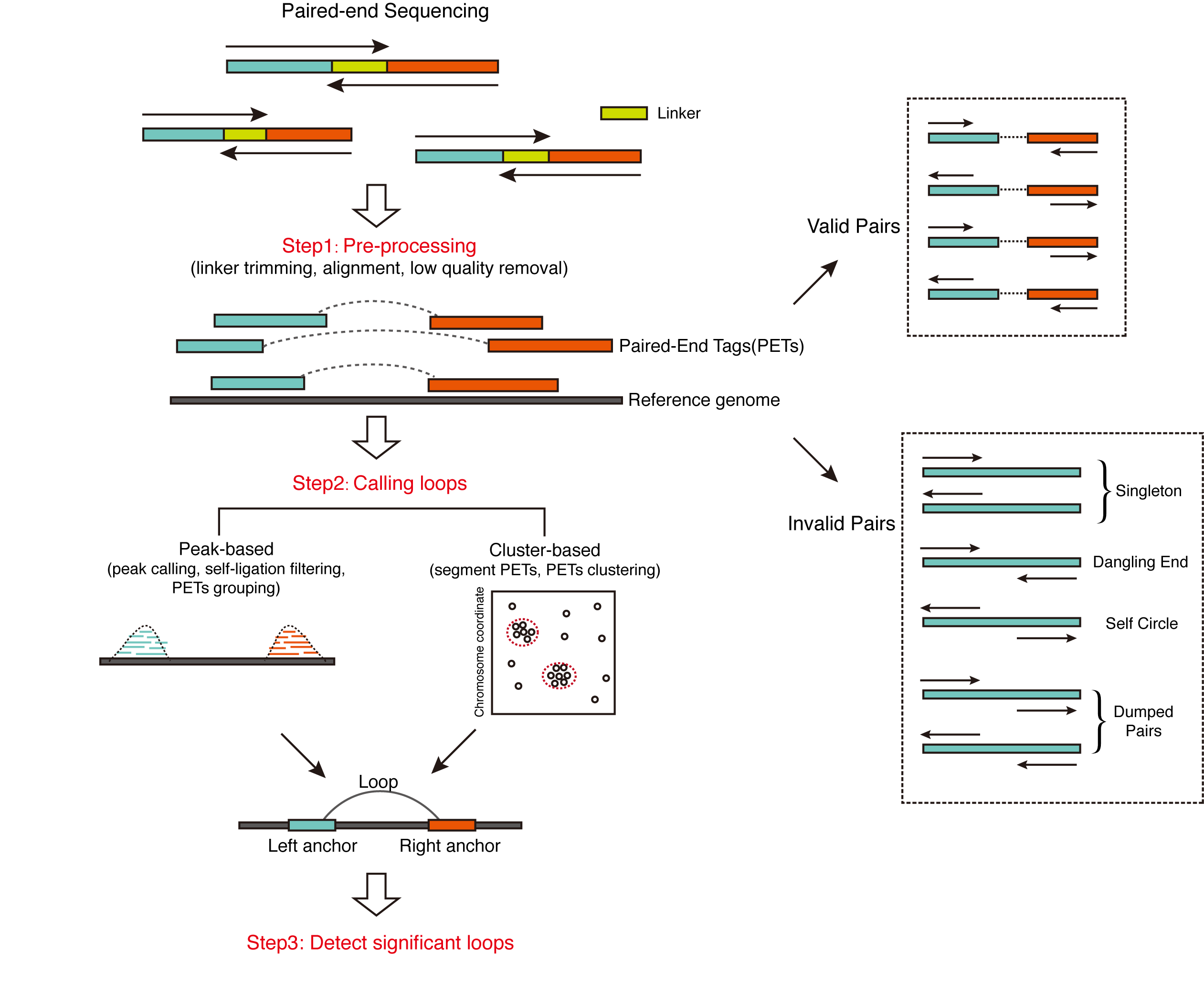


**Figure S2**. Schematic detailing the procedure of data processing, and the types of reads detected from HiChIP and ChIA-PET libraries. The processing can be characterized as three steps: pre-processing, calling loops, and the calculation of loop significance. Two dash boxes show the valid PETs and invalid PETs, the valid PETs are expected to involve two fragments, while the PETs only involve one fragment are regarded as invalid, which can be classified into singleton, dangling end, self-circle, and dumped pairs.

1. **Comparison of methods popularity**

To compare the popularity of different methods and give a guidance for users to choose, we selected the most representative citation for each method. The total citation times for each method was counted Google Scholar, up to August 2021 (**Table S1**).

**Table S1. Comparison of methods popularity**

| **Category** | **Methods** | **Year** | **Citation Times** | **Representative citation** |
| --- | --- | --- | --- | --- |
| ChIA-PET | Mango | 2015 | 98 | “Reads supporting contacts called using the Mango pipeline for GM12878 Smc1a HiChIP and GM12878 CTCF Advanced ChIA-PET” (Maxwell *et al*., *Nature*, 2016) |
|  | ChIAPoP | 2019 | 2 | “model-based Interaction calling from ChIA-PET data employs a hierarchical mixed probability model and ChIAPoP relies on a Poisson model” (Hernández-Lemus *et al.*, *Genes*, 2019) |
|  | ChIA-PET2 | 2017 | 55 | “The ChIA-PET2 software was used for quality control and identification of chromatin interactions” (Xiao *et al.*, *Cell*, 2019) |
|  | CPTv.3 | 2019 | 6 | “The package designed for ChIA-PET data processing” (Hong *et al.*, *Genes*, 2020) |
|  | Chiasig | 2014 | 39 | “Several computational pipelines have thus been developed to estimate background contact frequencies based on this genomic proximity bias” (Hnisz *et al*., *Science*, 2016) |
| HiChIP | Hichipper | 2018 | 63 | “raw reads were processed and loops identified using hichipper” (Zirkel *et al.*, *Molecular cell*, 2018) |
|  | MAPS | 2019 | 41 | “Chromatin loops were identified between active promoters and distal regulatory regions in microglia, neurons, and oligodendrocytes” (Nott *et al.*, *Science*, 2019) |
|  | FitHiChIP | 2019 | 41 | “H3K4me3 and H3K27me3-HiChIP loops were identified using FitHiChIP” (William *et al.*, *Nature plants*, 2019) |
|  | HiCCUPS | 2016 | 83 | “The data was aligned against the hg19 reference genome. All contact matrices used for further analysis were KR-normalized with Juicer” (Rao *et al.*, *Cell*, 2017) |
| For both | CID | 2019 | 3 | “Raw read files were analyzed with HiC-Pro, and interactions were subsequently called by CID” (Krismer *et al*., *Nucleic acids research*, 2020) |
|  | cLoops | 2019 | 17 | “using the cLoops peak-calling package33 with stringent statistical filtering (Methods), we identified 1,985 such point interactions genome-wide in pachytene spermatocytes” (Alavattam *et al*., *Nature Structural & Molecular Biology*, 2019) |
|  | MICC | 2015 | 25 | “Significant interactions were calculated using MICC” (Rowley *et al*., *Molecular cell*, 2017) |

1. **Comparison of commonly used pre-processing steps**

Most ChIA-PET-specific analytical methods were developed to handle the whole procedure from pre-processing through to significance detection. Within the pre-processing procedure, different linker trimming strategies and mapping tools have been used (**Table S2**). Since there was no clear description for the linker mismatch performed by mango (fixed setting), we tried 0 and 1 mismatch for ChIAPoP and ChIA-PET2. And for ChIA-PETv.3 we used the default linker alignment score, the results showed that the number of mismatches caused only a small impact on the UV Rate (**Fig. S3**). For alignment, the minimum mapping rate (MAPQ) was set 30. Although we set the same filtering threshold for different methods, the different fixed settings specific to each method impacted the UV rate. The difference was primarily caused by the key parameters passed on to the different alignment tools. ChIA-PET analysis methods fixed key parameters for the alignment tools, such as CPT2 which integrates BWA to perform alignment, and allows only two fixed optional modes: “bwa aln” (for reads around 20bp) and “bwa mem” (for reads >70bp). CPTv.3 also integrates BWA to perform mapping, while the fix parameters were “bwa aln” and “bwa samse”. The different modes of alignment tools can cause the prepreocessing results to vary a lot.

**Table S2**. Comparison of pre-processing strategies

| **Category** | **Methods** | **Mismatch in linker** | **Mapping tools** |
| --- | --- | --- | --- |
| ChIA-PET | ChIAPoP | Allow to set | Bowtie |
|  | Mango | Fix setting | Bowtie |
|  | ChIA-PET2 | Allow to set | BWA |
|  | ChIA-PETv.3 | Defined linker alignment score | BWA |
| HiChIP | HiC-Pro | — | Bowtie2 |
|  | MAPS | — | BWA |


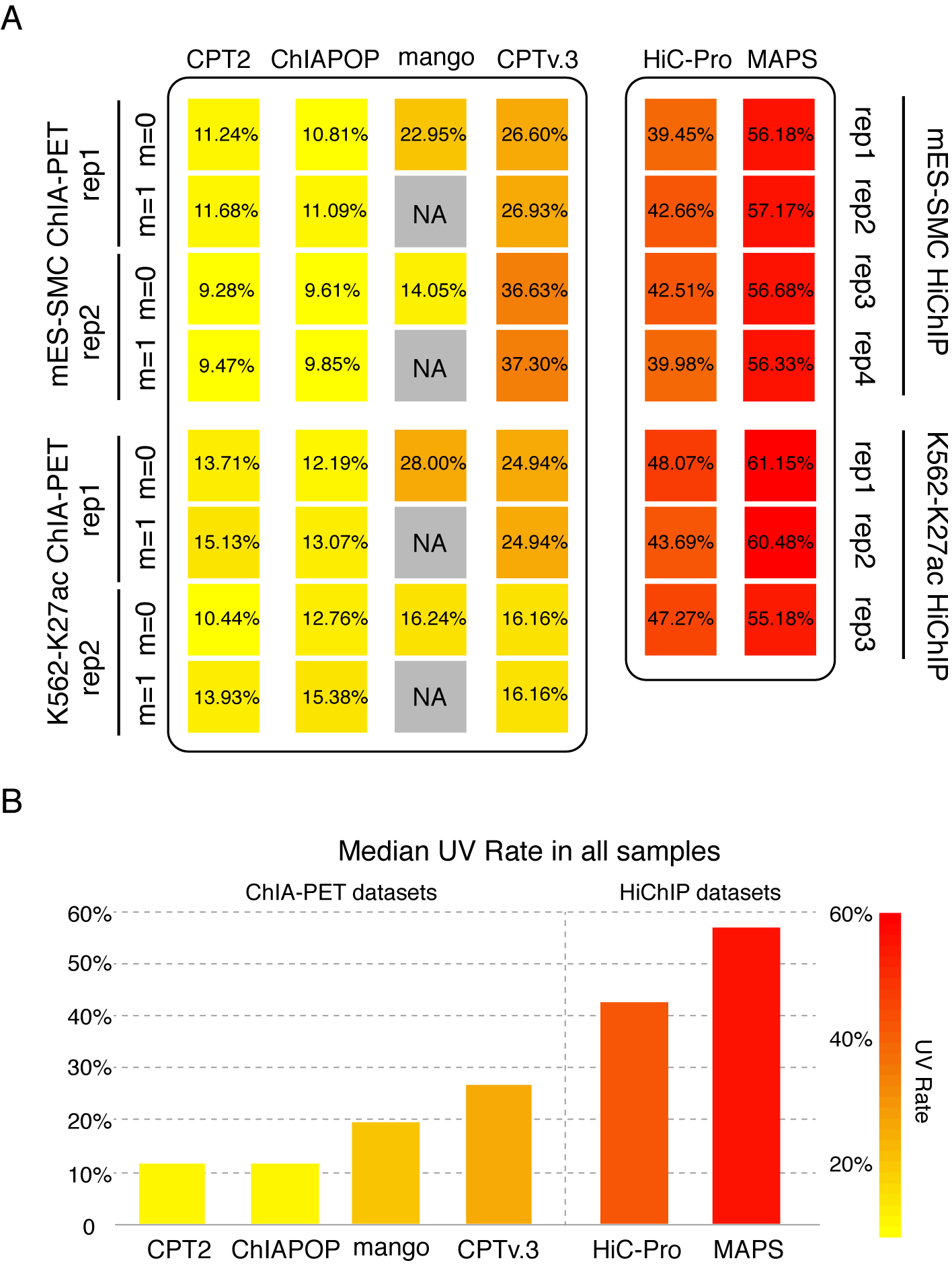


**Figure S3. Uniquely mapped Valid PETs Rate (UV Rate) differ between HiChIP and ChIA-PET datasets.** The UV Rate of different computational methods calculated per individual sample. ChIA-PET2 (CPT2) and ChIA-PET Tool V3 (CPTv.3). The number of mismatches considered during linker trimming for ChIA-PET data, 0 mismatch (m=0), 1 mismatch (m=1). NA, data not available.

1. **Comparison of all HiChIP PETs and short-range PETs**

As an alternative approach to HiChIP peak calling, Mumbach *et al.* recommended using only self-ligation and dangling-end reads (S+D) to detect peaks. To compare the effectiveness of different peak calling parameters, we used only self- and dangling- HiChIP (S+D) reads, all HiChIP (A) reads, as well as ChIA-PET reads to call peaks, we then overlapped the ChIP-seq peaks with these peaks. The highest overlapping percentage of peaks called (41.3%) came from ChIA-PET peaks, and HiChIP (S+D) overlapped more than HiChIP (A) (**Fig. S4A**). Therefore, using only self- and dangling- HiChIP (S+D) reads can reduce the background signal effectively from HiChIP data. We next selected high-confidence peaks (q-value < 0.01) from each dataset. The number of HiChIP (S+D) peaks reduced over 2-fold compared with HiChIP (A) peaks (**Fig. S4B**). And the width of HiChIP (S+D) peaks also reduced significantly (**Fig. S4C**).

**
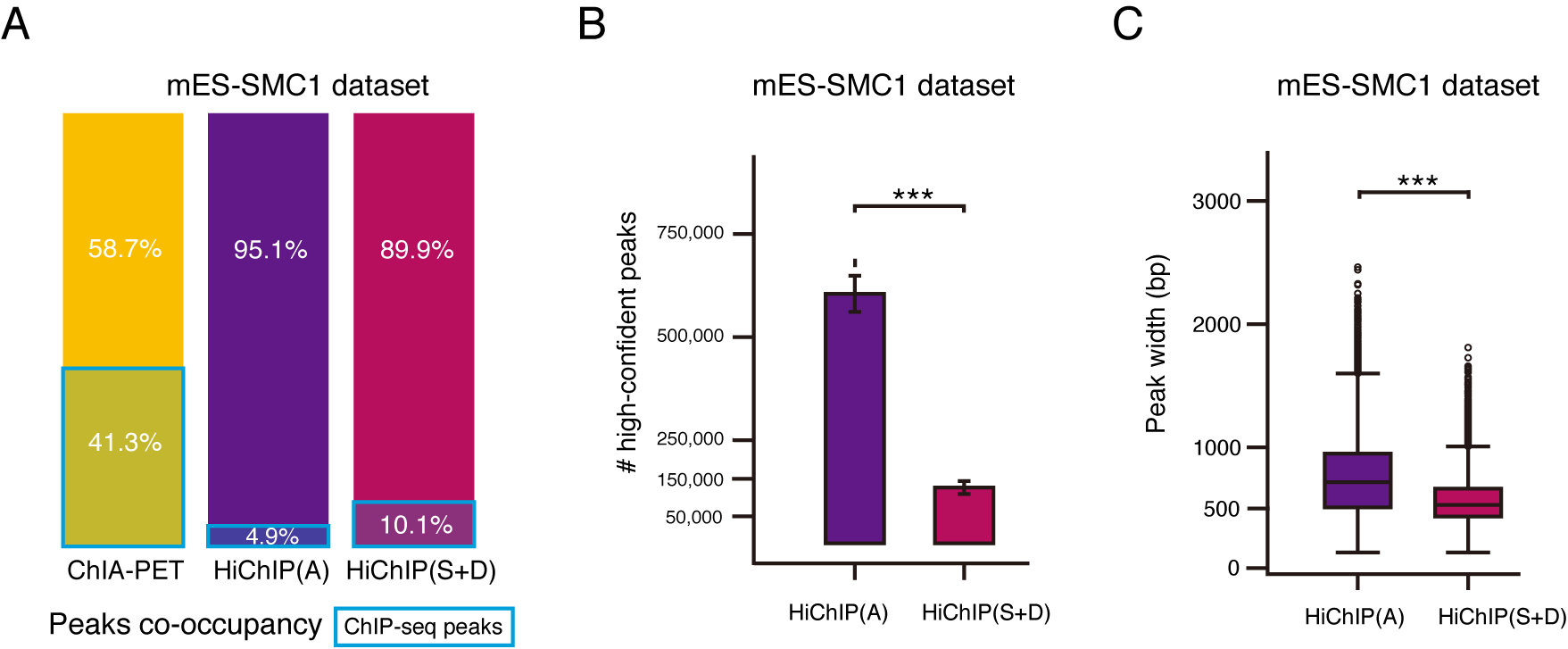
**

**Figure S4. Comparison of all HiChIP PETs and short-range PETs.** (A) Peak co-occupancy between ChIP-seq and ChIA-PET, HiChIP(S+D) and HiChIP(A). (B) Number of high-confident peaks in HiChIP(S+D) and HiChIP(A). (C) The width of peak in HiChIP(S+D) and HiChIP(A). Bar plot drawn with standard error bar. ***, p-value <1e-3, p-value was calculated by t-test.

1. **Comparison of cluster-based methods**

Currently, there are very few cluster-based methods available. Only CID and cLoops packages are applicable for performing HiChIP and ChIA-PET analysis. We applied both and found that that these algorithms produced more loops when applied to HiChIP than ChIA-PET datasets. The results also indicated that CID output more loops than cLoops (**Fig. S5**)


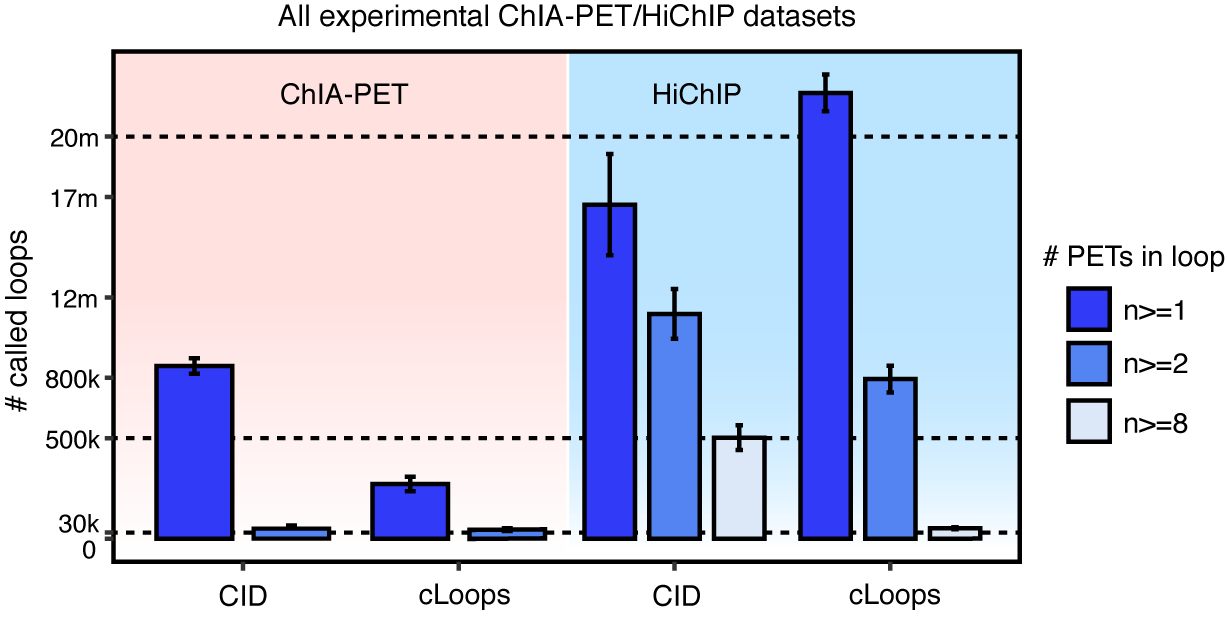


**Figure S5.** The number of loops detected by two cluster-based methods. The color legend indicated different ranges of PET counts in loop

We next wanted to more thoroughly compare the loop enrichment outputs from both CID and cLoops. Polycomb domains (PDs) and super-enhancer domains (SDs) are typically located within a loop between two interacting CTCF sites co-occupied by cohesin. PDs play important roles in maintaining the pluripotent ESC state, while SDs are considered important for normal expression of genes located in the vicinity of the SD. The *Gata2* locus lies within a PD (**Fig. S6**), and the *Nanog* gene, which encodes a key pluripotency transcription factor, is located within an SD (**Fig. S7**). At the *Gata2* locus, the 2D density plots showed that both ChIA-PET and HiChIP can detect obvious read enrichment in anchor regions (**Fig. S6A, S6C)**. While at the *Nanog* locus, there is no obvious enrichment in the anchor regions when you focus on HiChIP read density (**Fig. S7C)**. We next calculated ES performance of cLoops and CID by genomic coordinate and aligned these results to both loci **(Fig. S6C, S6D, S7C, and S7C).** In the upstream anchor region of the *Gata2* locus, cLoops produced a higher ES than other non-anchor regions, while CID didn’t output higher enrichment in this anchor (**Fig. S6B)**. Similarly, for the SD anchors of the *Nanog* locus locus, cLoops outperformed CID (**Fig. S7B, S7D)**. However, for the HiChIP output at the *Gata2* locus, neither method produced an obvious ES near the anchor regions (**Fig. S6D)**.


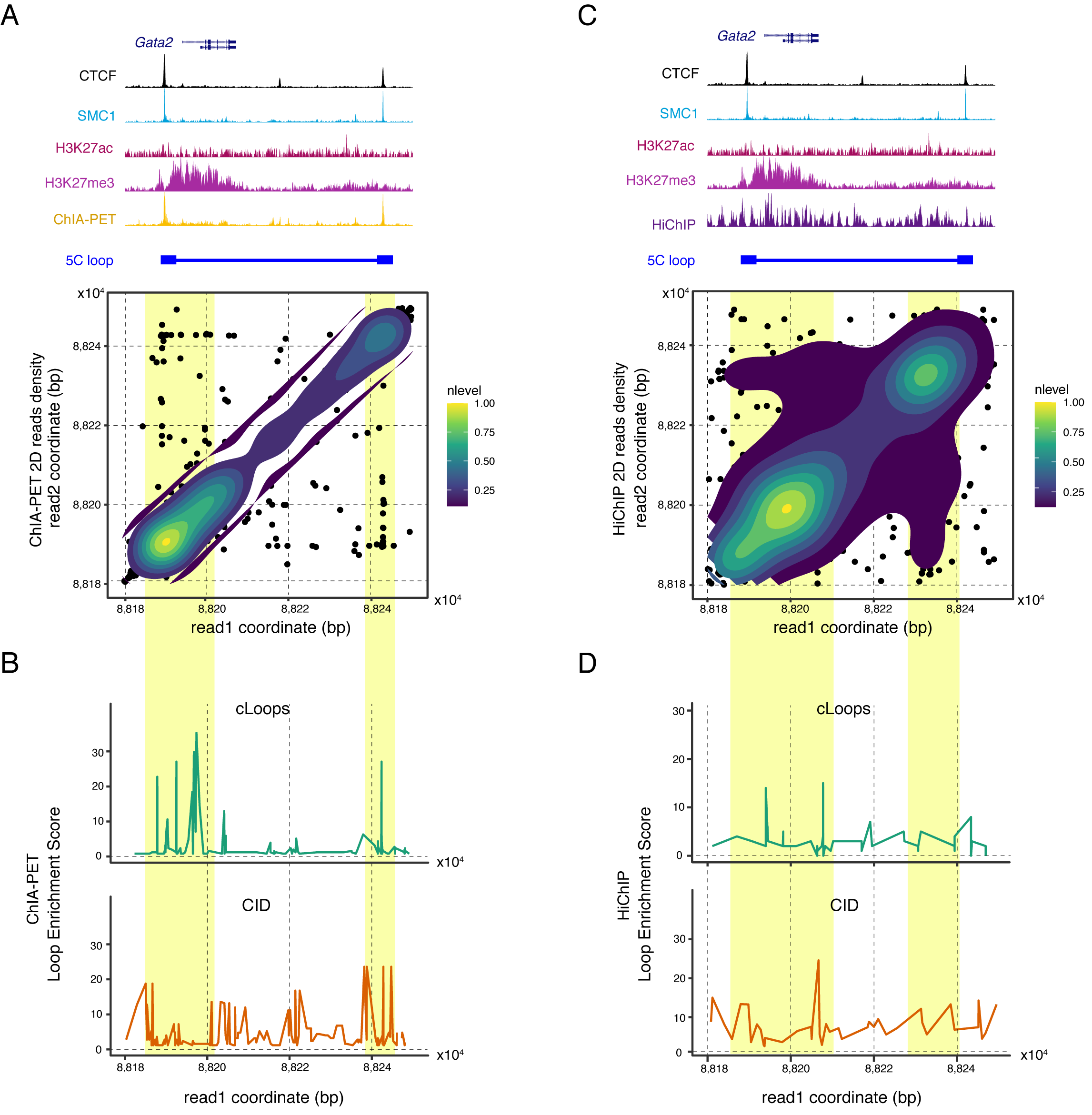


**Figure S6.** (A) The ChIP-seq tracks near *Gata2* locus, the blue line represented loop within domain which has been verified by 5C, and the blue squares at two ends were anchors. The ChIA-PET read enrichment represented by 2D density plot. (B) The loop enrichment score of cLoops and CID by genomic coordinates near *Gata2* locus. (C) The HiChIP read enrichment represented by 2D density plot. (D) The loop enrichment score of cLoops and CID by genomic coordinates near *Gata2* locus.


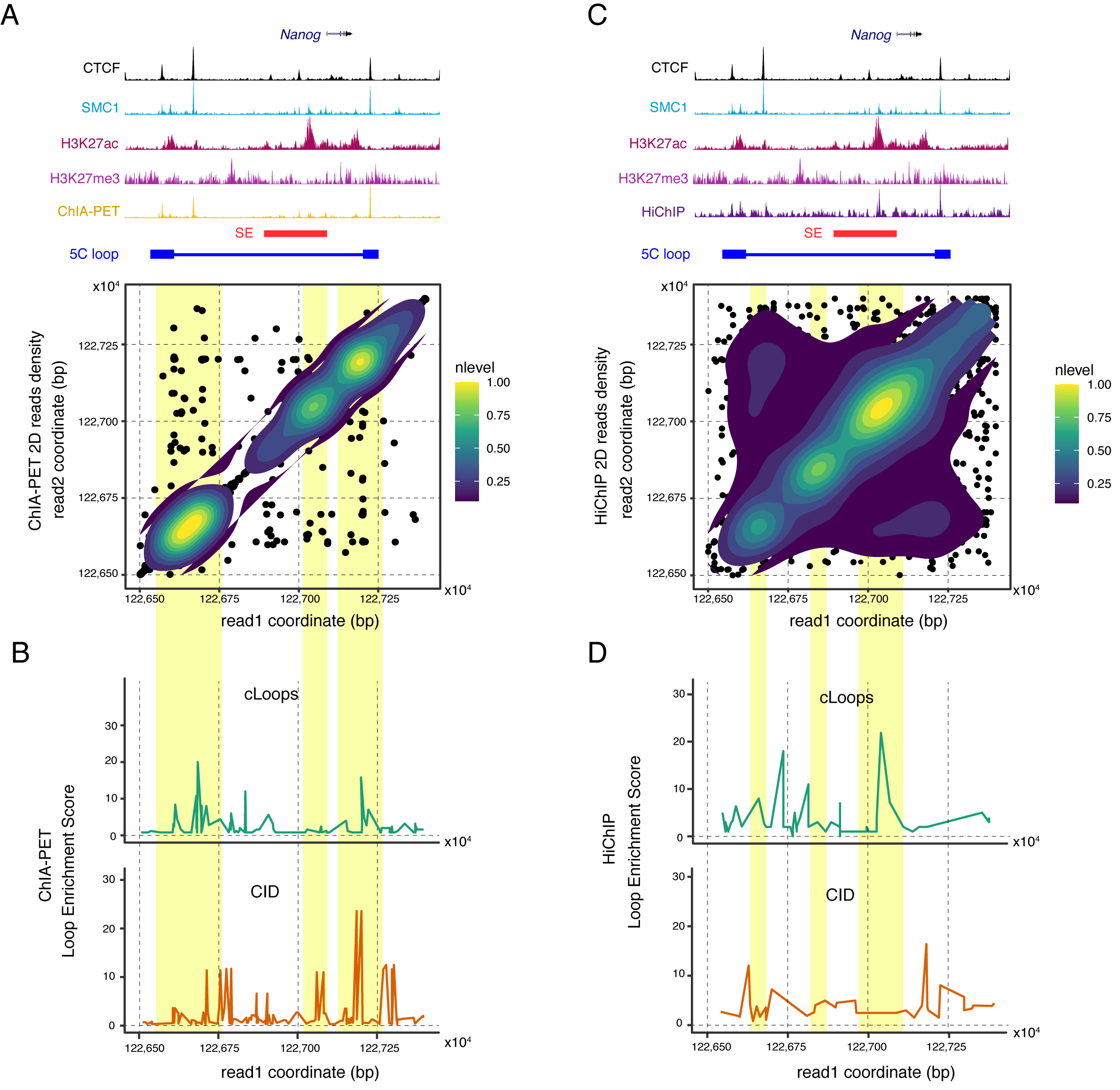


**Figure S7.** (A) Genome browser tracks at the *Nanog* locus. The blue line represent the loop within the contact domain which has been verified by 5C, and the blue squares signify the anchors. The ChIA-PET read enrichment is represented by a 2D density plot. (B) The loop enrichment score of cLoops and CID by genomic coordinates at the *Nanog* locus. (C) HiChIP read enrichment represented by 2D density plot. (D) The loop enrichment score of cLoops and CID by genomic coordinates near the *Nanog* locus.

1. **Comparison of different loops with peak intensity and chromatin state**

We used Principle Component decomposition to compare the differences between loop calls. For ChIA-PET datasets, the loops called by four peak-based methods (ChIAPoP, mango, CPT2 and CPTv.3) were similar, while the loops output from two cluster-based methods appeared as distinct from each other (**Fig. S8A**). The evaluation of peak intensity from loop anchors showed that ChIAPoP loops produced the highest signal, and the two cluster-based methods didn’t show any obvious intensity differences near peak center (**Fig. S8B-S8C**). For HiChIP data, we noticed that the loops detected by Hichipper clustered together (**Fig. S8D**), and among the different parameters evaluated Hichipper(+chip) output the highest peak intensity (**Fig. S8E-S8F**).

To investigate the differences between all methods, we overlapped the optimal loops called by ChIAPoP with the other ChIA-PET tool outputs individually. The results showed higher overlapping percentages for the three peak-based methods (ranges from 69.2% to 95.2%), while the two cluster-based methods overlapped less than 10% with ChIAPoP (**Fig. S8G)**. For HiChIP-specific analysis methods, we overlapped the optimal loops called by Hichipper(+chip) with the other widely-used methods. We found that the cluster-based method CID generated the highest overlapping percentage with Hichipper(+chip) (**Fig. S8H)**.

We next wanted to further investigate the properties of different loops called by these methods. We chose ChIAPoP as the representative ChIA-PET peak-based method, and compared the ChIAPoP-specific loops with cLoops-specific, and CID-specific loops with two histone marks. The results showed that ChIAPoP produced higher H3K27ac signals than cLoops and CID, while cLoops and CID output higher H3K27me3 signals than ChIAPoP (**Fig. S9A**). Suggesting that the peak-based methods detected more active loops than cluster-based methods, while cluster-based methods detected more inactive loops. For HiChIP analysis methods, we chose Hichipper(+chip) as representative to compare against other pipelines. The HiChIP-specific comparison showed that the peak-based methods detected more active loops than the cluster-based methods, and cluster-based methods detected more inactive loops (**Fig. S9B**).


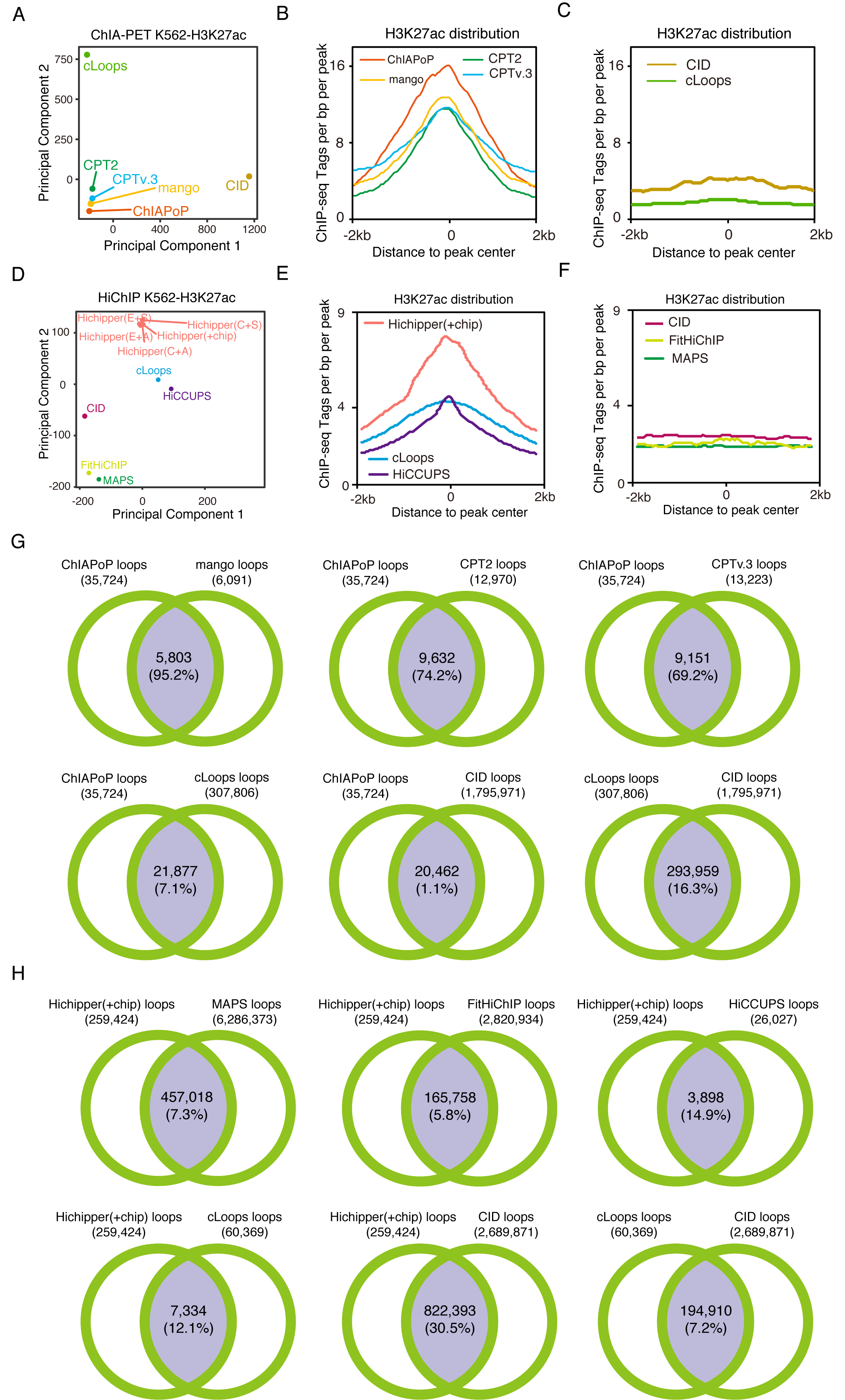


**Figure S8. Comparison of different loops with peak intensity.** (A) Principal component analysis of loops detected by different methods in K562-H3K27ac ChIA-PET dataset. (B) H3K27ac ChIP-seq Peak density of loops (detected by ChIAPoP, mango, CPT2, and CPTv.3). (C) H3K27ac ChIP-seq Peak density of loops (detected by cLoops and CID). (D) Principal component analysis of loops detected by different methods in K562-H3K27ac HiChIP dataset. (E) H3K27ac ChIP-seq Peak density of loops (detected by Hichipper(+chip), FitHiChIP, and MAPS). (F) H3K27ac ChIP-seq Peak density of loops (detected by CID, cLoops, and HiCCUPS). (G) Overlap between different ChIA-PET analytical methods. (H) Overlap between different HiChIP analytical methods.

**
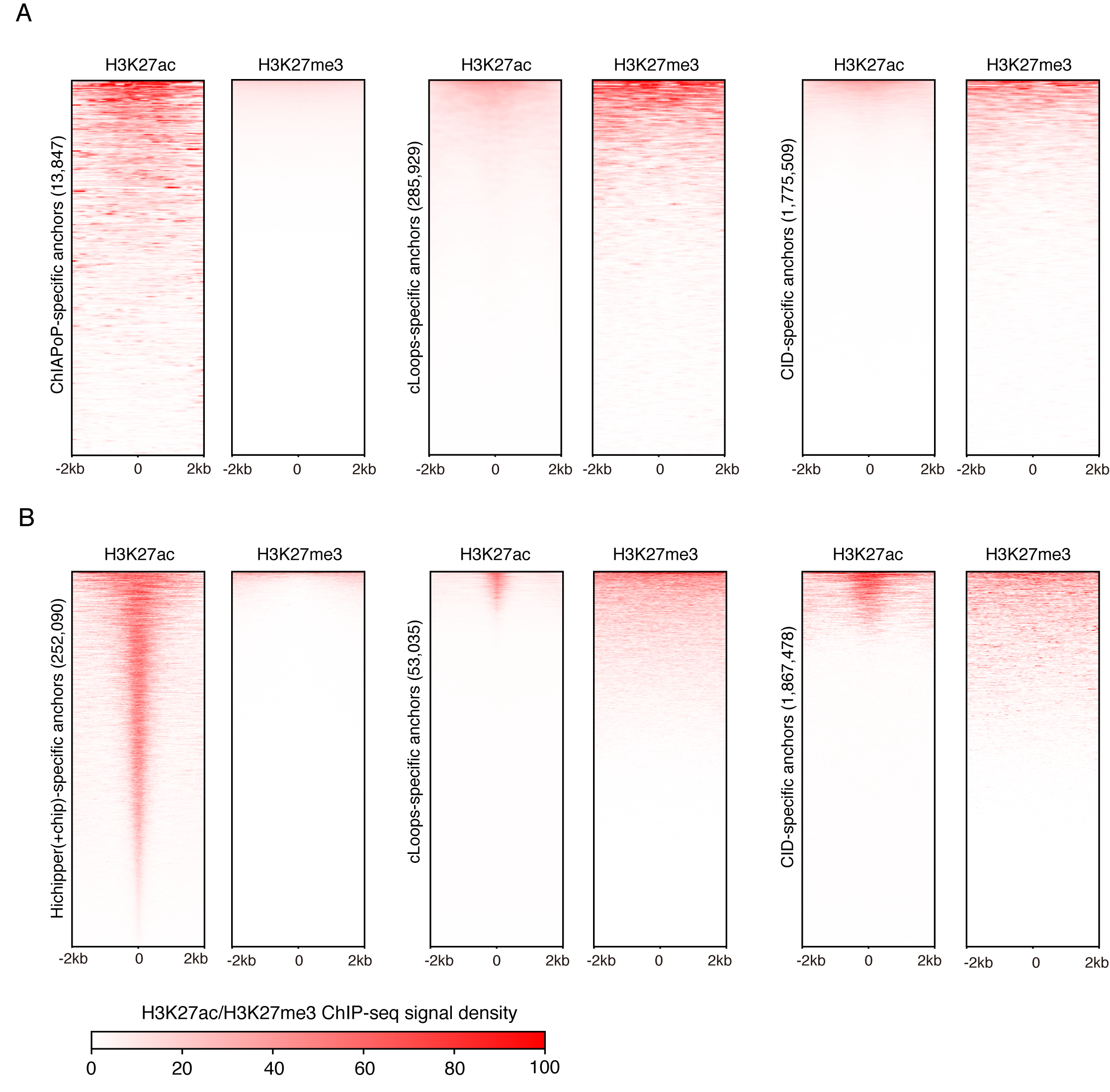
**

**Figure S9. Comparison of different loop anchors with histone marks.** (A) Heatmaps showing the comparison of ChIAPoP-specific loop anchors and two cluster-based methods loop anchors. (B) Heatmap of Hichipepr(+chip)-specific loop anchors and two cluster-based methods loop anchors.

1. **Evaluating the accuracy of loops**

To ensure the fairness of comparison, we generated three golden loop sets for each testing dataset with different significance thresholds (for the gathering of golden loop set, see methods). The accuracy (ACC) was then calculated with True Positive (TP), False Positive (FP), True Negative (TN) and False Negative (FN) metrics (for the calculation of ACC, see methods).

The ACC of loops was independent of the number of loops. And the ACC of ChIA-PET loops was higher than that of HiChIP loops across the 8 testing datasets (**Fig. S10A** and **S10B**). To investigate which characteristics impacted the results of ACC, we next calculated Pearson’s correlation coefficient for ACC and the other three evaluation metrics. The results suggested that UV Rate weakly correlated with ACC (**Fig. S10C**), while PC and ES were positively correlated with ACC (**Fig. S10D** and **S10E**).

**
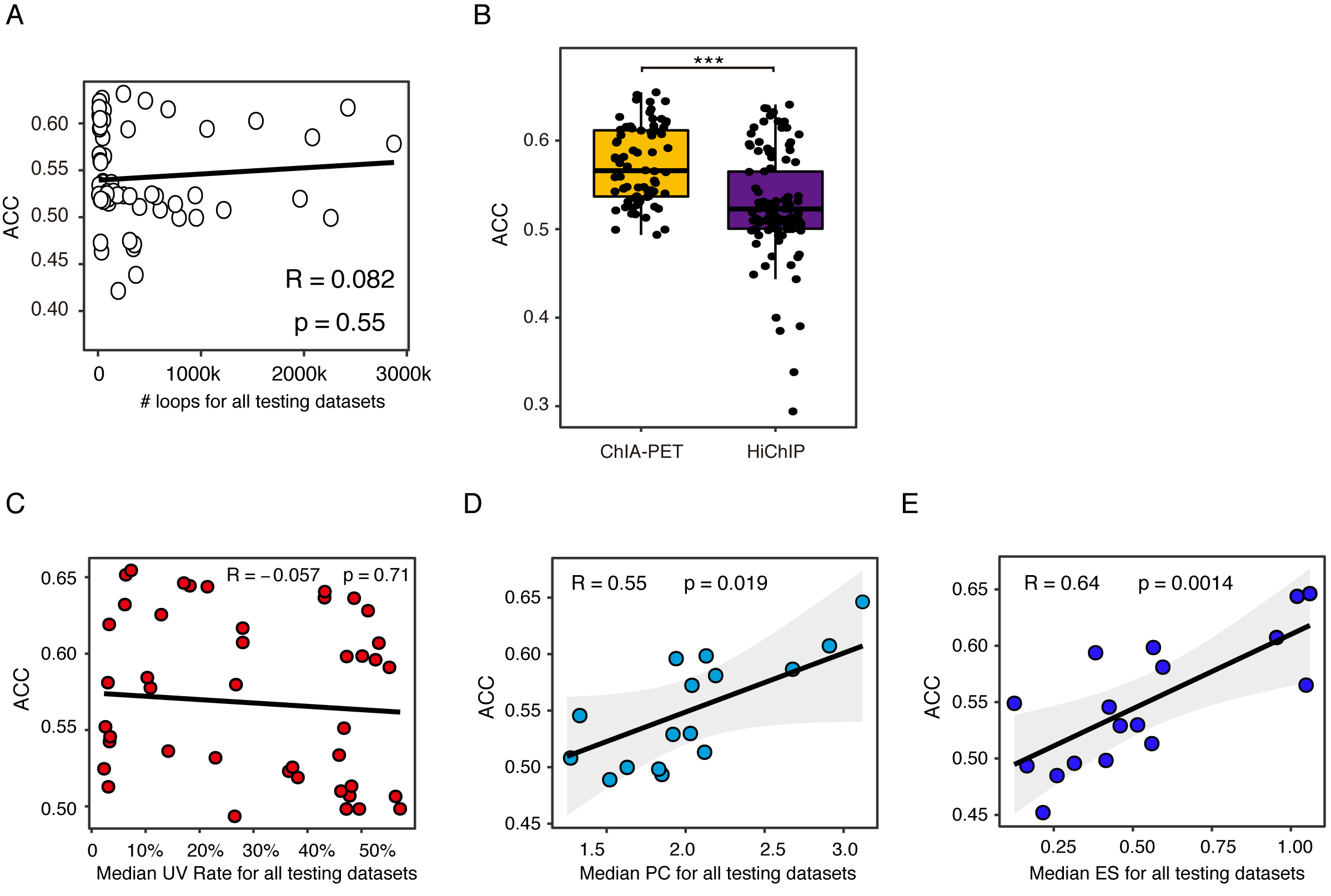
**

**Figure S10. Evaluating the accuracy of loops.** (A) The Pearson’s correlation between ACC and the number of loops. (B) The ACC of ChIA-PET and HiChIP datasets. (C) The Pearson’s correlation between ACC and UV Rate. (D) The Pearson’s correlation between ACC and PC. (E) The Pearson’s correlation between ACC and ES. R, correlation coefficient. p-value of correlation was calculated by t-test. *** p-value<1e-3, p-value was calculated by t-test.

1. **Comparison between original and long read ChIA-PET protocols**

The original ChIA-PET protocol generates short read pairs around 20bp, which identified the genomic interactions effectively, while the long-read protocol increased the length of paired-end tags to 150 bp, which improved the alignment rate and the overall accuracy. Since there was no published GM12878-RNAPII HiChIP data available, we only compared the original ChIA-PET protocol with the long-read ChIA-PET data here. Within all the ChIA-PET analytical methods, only ChIA-PET2 (CPT2) and ChIA-PETv.3 (CPTv.3) supported the raw data analysis of long-read ChIA-PET data, thus we compared the performance of the latest approach ChIA-PIPE with CPT2 and CPTv.3. To compare the efficiency and sensitivity of the two protocols, we calculated the UV Rate from these datasets, the results showed that the long-read protocol detected more uniquely valid PETs, which was identical to the results from a previous study. In addition, all the methods achieved higher peak occupancy (PC), accuracy (ACC), and active rate (AR) with the long-read protocol. Overall, ChIA-PIPE performed slightly better than CPT2 and CPTv.3 for both types of datasets (**Table S3**).

**Table S3. Comparison between original and long-read protocols**

|  | GM12878  Original ChIA-PET | GM12878  Long read ChIA-PET |
| --- | --- | --- |
| *Target protein* | RNAP II | RNAP II |
| *Sequencing length* | 2 × 20 bp | 2 × 150 bp |
| *UV Rate* | 34.0% ± 3.2% | 49.2% ± 3.0% |
| *PC(CPT2)* | 0.7230 | 0.8321 |
| *PC(CPTv3)* | 0.7405 | 0.8846 |
| *PC(ChIA-PIPE)* | 0.7983 | 0.8560 |
| *ACC(CPT2)* | 0.5320 | 0.5735 |
| *ACC(CPTv3)* | 0.5678 | 0.5915 |
| *ACC(ChIA-PIPE)* | 0.6172 | 0.6392 |
| *AR(CPT2)* | 0.7406 | 0.7931 |
| *AR(CPTv3)* | 0.8174 | 0.8243 |
| *AR(ChIA-PIPE)* | 0.8055 | 0.8331 |

1. **Comparison of reproducibility**

We performed reproducibility analysis for the all methods. We firstly combined the loops output by different replicates from each tool, and the number of combined loops for tool *i* was defined as *N_i_*, then we intersected the loops of different replicates, the number of loops called by all replicates was defined as *n_i_*, the reproducibility of tool *i* was defined as ${n_{i}}/{N_{i}}$. The reproducibility results showed that Hichipper with “C+S” and “C+A” modes achieved the best reproducibility in all HiChIP datasets, followed by Hichipper(+chip) (**Fig. S11**).


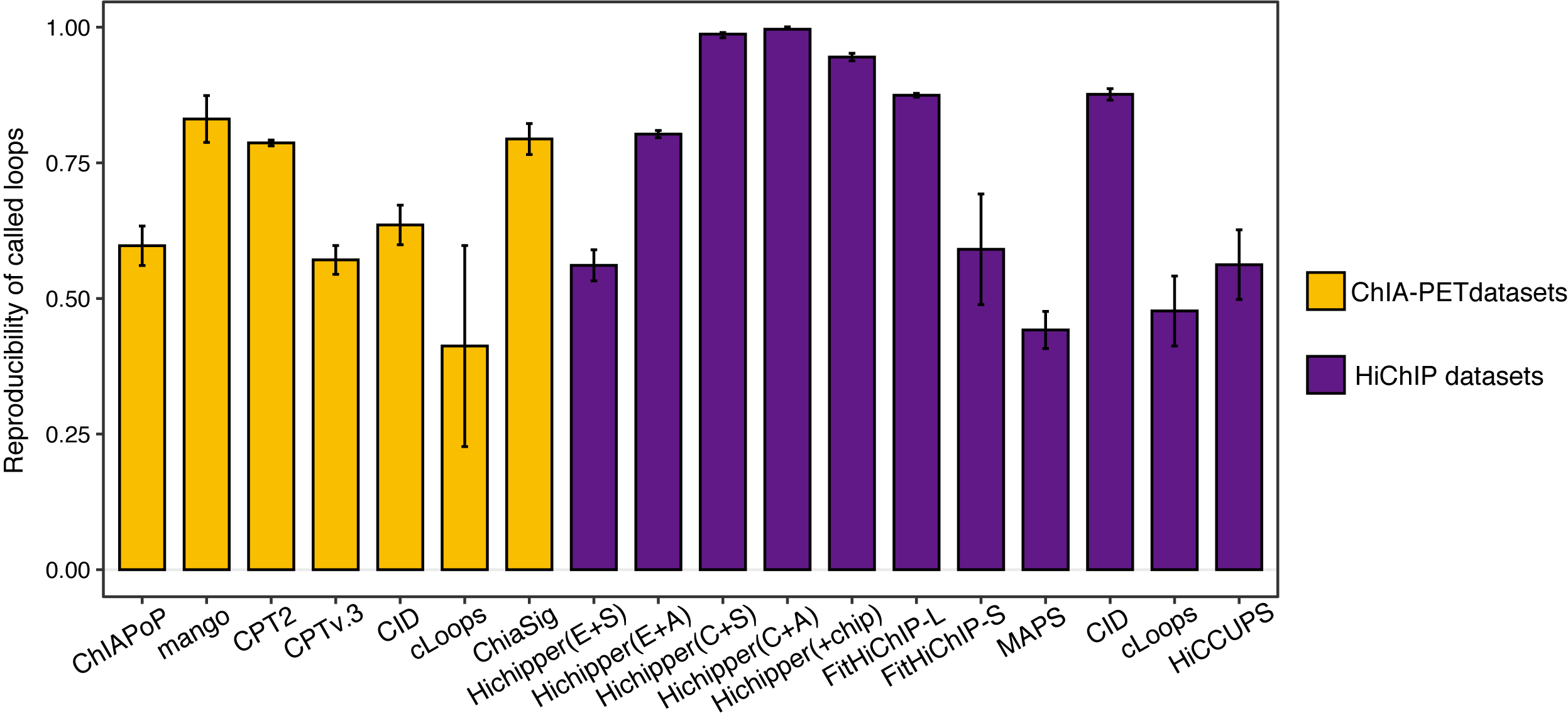


**Figure S11**. Comparison of reproducibility.

1. **Comparison of running time**

The mean running time of different methods where calculated after preprocessing. Since the alignment step of these methods was implemented by other mapping tools (like BWA, Bowtie), here we only counted the running time of linker trimming, calling loops, and detecting the significance of loops for the ChIA-PET analytical methods (**Table S4**). As most of the HiChIP methods didn’t allow running a single analysis step, we counted the total running time of calling loops and detecting significance of loops for HiChIP analytical methods (**Table S5**).

**Table S4. Running time of ChIA-PET methods**

|  | \| **Trim linker** \| \| --- \| | **Call loops** | **Detect significance** |
| --- | --- | --- | --- | --- |
| CPT2 | 33m30.213s | 77m23.194s | 72m44.413s |
| ChIAPoP | 54m44.540s | 257m51.898s | 68m19.476s |
| CPTv.3 | 55m16.291s | 84m5.758s | 2m30.024s |
| mango | 14m59.074s | 37m16.724s | 17m56.702s |
| CID | **—** | 57.852s | 447m15.852s |
| cLoops | **—** | 168m18.471s | 55m9.602s |
| Chiasig | **—** | **—** | 262m11.782s |

**Table S5. Running time of HiChIP methods**

|  | **Call loops & Detect significance** |
| --- | --- |
| Hichipper(E+S) | 63m17.901s |
| Hichipper(E+A) | 120m57.253s |
| Hichipper(C+S) | 148m17.585s |
| Hichipper(C+A) | 133m54.787s |
| Hichipper(+chip) | 17m4.740s |
| MAPS | 52m19.251s |
| FitHiChIP-L | 54m41.099s |
| FitHiChIP-S | 42m14.139s |
| CID | 139m25.29s |
| cLoops | 370m24.243s |
| HiCCUPS | 25m52.10s |
