# Supplementary material for: Bacon: a comprehensive computational benchmarking framework for evaluating targeted chromatin conformation capture-specific methodologies
Source: Genome Biol. 2022 Jan 21;23:30. doi: 10.1186/s13059-021-02597-4 (PMC8780810; doi:10.1186/s13059-021-02597-4)
Supplement: Supplementary file 5 — Additional file 5. Review history. [file 13059_2021_2597_MOESM5_ESM.docx]

Review History

**First round of review**

**Reviewer 1**

**Were you able to assess all statistics in the manuscript, including the appropriateness of statistical tests used?**

No, I do not feel adequately qualified to assess the statistics.

**Comments to author:**

In this manuscript, Tang et al develop a computational benchmarking framework for comparing targeted chromatin conformation capture-specific methods. This is novel, to my knowledge. I am familiar with Forcato et al., Nature Methods, 2017, which compares computational methods for Hi-C, but this is done with Hi-C and not for methods such as Hi-ChIP and ChIA-PET. Overall, the paper is important to the field, as comparison of software is necessary for researchers to know which are the optimum tools for use. However, a major criticism that I have is that it is hard to know what are the "true" loops. Forcato et al., Nature Methods, 2017 overcame this challenge by using simulated data. Therefore, I am wondering if Tang et al can similarly develop simulated data for comparison between different software.

Additional comments

1. In the paper, besides mentioning the new HiChIP method, I think it is important to also cite the newer papers with newer and easier to use versions of ChIA-PET. So for this point "The recently developed HiChIP [18]and PLAC-seq [19]protocols reveal better resolution and require lower chromatin input than ChIA-PET." - I think is only valid when compared with the old, traditional ChIA-PET. I think the new ChIA-PET papers which have new long reads protocols should also be mentioned as they are also better in resolution and require lower chromatin input. In analysing the data and comparing methods, it would also be good to refer to data from these newer papers.

2. I am also interested to see what are the recommendations like, and if they are different from the older, traditional ChIA-PET and the newer long ChIA-PET approaches.

3. Lines 86-88 "However,the differences between ChIA-PET and HiChIPprotocolsrender many of thepeak-based computational methods not applicable to both ChIA-PET and HiChIP data simultaneously." => I am interested to know what peak-based methods are not applicable. Is MACS2 not applicable? Or is it OK but others are not, and if so, why? What are these differences that complicate such analyses?

4. There are a lot of references to concepts such as loop anchors, splitting PETs, and so on. I think a glossary and/or schematic figures in the Supplement would greatly aid the readers in understanding these concepts.

5. The authors make reference to "popular Hi-ChIP-specific software package[s]" such as Hichipper. I was wondering if the authors could include a supplementary table or supplementary information comparing the various kinds of software, and also mentioning what are the papers that these methods have been used in? This will help to give the readers some idea of the comparisons between these different methods and their popularity. Also, the authors discuss the run time of the various methods, and a table comparing the run times of the different methods would be useful.

6. In Figure 4, the authors compare some of the characteristics of the loops between the different softwares. I am curious to know what is the degree of similarity in loop calls between different methods? What loops are called differently by different methods, and is there any reason for the different loop calls? I think this will help to further guide the recommendations.

7. In the discussion, the authors said "Overall,improved computational methods are urgently needed to effectively detect chromatin interactions from these datasets" but did not give any suggestions for what should be improved in each method. Based on Bacon, can the authors identify some points where improvements can be made? Would running two different methods and comparing the data between them help to improve the quality of loops called?

Minor comments

8. On https://csuligroup.com/Bacon/Recommendation, under recommendations, "Hichipper(ref)" is listed. Is "ref" supposed to be there? You can also see this in the paper at line 353.

9. The manuscript is quite understandable, but still has some grammar errors. Another round of proof-reading would be helpful.

10. Lines 54-55 "Chromatin interaction analysis by paired-end tag sequencing (ChIA-PET) is a technique which combines ChIP and 3C" => Technically, it is a combination of ChIP, 3C and next-gen sequencing. The combination of ChIP and 3C is ChIP-3C.

**Reviewer 2**

**Were you able to assess all statistics in the manuscript, including the appropriateness of statistical tests used?**

Yes, and I have assessed the statistics in my report.

**Comments to author:**

In this paper, the authors presented a comprehensive benchmark framework, Bacon, to evaluate the performance of 12 computational methods (but only 10 showed in the workflow), and provide practical recommendations for users working with HiChIP and/or ChIA-PET analysis. The detected significant loops were validated by epigenomic features and published Hi-C profiles. The evaluation results indicated that no single method outperforms the others in every aspect, and that improved computational methods are needed to effectively detect chromatin interactions. Indeed, it is very disappointing that there is no good suggestion for choosing a tool. I have several serious concerns about the work.

Major:

1. It is not necessary to include "preprocessing" step in the evaluation since the data quality is from experimental parts and can be QC using many other mapping tools.

2. The better way to evaluate the tools with biological replicates. Since both HiChIP and ChIA-PET have biological replicates, the authors should examine the reproducibility of binding peaks and called loops from biological replicates by each of 12 tools.

3. For Peak Co-occupancy (PC), the authors should clarify the novelty of PC compared to the results from diffBind, as both methods tend to address how many 'shared peaks/diff peaks' between ChIP-seq peaks and ChIA-PET/HiChIP peaks. For me, the diffBind used a reliable statistical inference to identify the differentiated peaks but PC just simply found 1bp overlapped peaks between ChIP-seq peaks and ChIA-PET/HiChIP peaks.

4. For Enrichment Score (ES), the authors assumed that 'the true enriched loci should be surrounded by relative low enriched PETs' before calculating the ES. Thus, the authors should verify their assumption either from previous literatures or from the data they used.

5. The authors should explain how to determine the value of ∝ in calculating global ES with low sequencing depth.

6. In P10L172, the authors claimed that MAPS performed better than HiC-Pro based on the result that unique valid rate of MAPS is higher than HiC-Pro's. However, as these two programs only performed read alignment and PETs filtering in the framework, the author should make sure that both programs used similar level of mapping and filtering with their parameters and clarify which mapping parameters or filters cause this big difference. Otherwise, the high UV rate might result from a more tolerant mapped setting or loose filtering process. The same concerns also in the UV rate in the ChIA-PET.

7. More analyses and visualizations should be performed to comprehensively tell the difference between programs, such as the distribution of the loop length from different programs; the peak intensity from different peak-based programs; histone modification signal on the anchors; visualizing identified loops from different programs on the Hi-C matrix.

8. Using epigenomic data and Hi-C data as validations is not a bad idea. However, the authors oversimplified it with a simple correlation of E-P looping vs enhancer marks. The authors should come up a more systematic way to validate the loops and binding peaks.

9. HiChIP-Peaks should be included in the comparison.

10. Figure 2B provides the same information in Figure 2A.

11. P18L289, H3K27ac peak is narrow-peak that recognize by ENCODE (https://www.encodeproject.org/chip-seq/histone/) and author should double-check the statement in the paper their cited.

Minor:

12. Is there any particular reason to choose mismatch 1? Since the larger mismatch allowed, the more reads will map to the reference genome. The result of mismatch 1 doesn't give any new information.

13. Figure. 3C is hard to understand, why are there two HiChIP peaks flank the Mbol Res. Motif?

14. MAPS uses feather to do the mapping part. It would be better to annotate MAPS with feather in Figure 1.

15. Figure 5B should be a joint plot instead of a Hexbin plot.

16. The authors should make the term consistent, e.g. P19L317 'Supplemental Figure. 5C' and P19L318 'Fig S5C'

17. There are too many typos in the manuscript, e. g. P21L338 uses the pass tense but P21L339 uses the present tense. The authors should carefully check the English grammar for all of the paper.

**Reviewer 3**

**Were you able to assess all statistics in the manuscript, including the appropriateness of statistical tests used?**

Yes, and I have assessed the statistics in my report.

**Comments to author:**

This manuscript describes a computational work to evaluate 12 analytical tools for two specific types of 3D genome profiling data, ChIA-PET and HiChIP. The authors compare the results from the tools on two datasets from each technique, one mouse cell type and one human cell line, respectively. The evaluation results indicate that no single method is superior. The manuscript is written clearly and straightforward to follow. While systematic comparison is important in the field and the data shown in this manuscript may be of interest to some researchers, I have some concerns on the design, approach, scientific rigor, and significance of this work. Specifically,

Major issues:

1. There are fundamental flaws on the study design. A benchmarking study comparing different methods should have uniform gold (or silver) standards and comprehensive evaluation criteria. It is not sufficient to simply compare results of the tools on 2 datasets. There are plenty of datasets generated from ChIA-PET and HiChIP, why the authors only chose these 2 particular datasets, and why these two datasets can be used for benchmarking, are completely unjustified. What if the results only reflect the quality difference on these 2 datasets? The quality of the benchmarking data should be thoroughly assessed before using them for benchmarking other tools. Besides, different results from different tools on data generated using different technologies are expected. How the different results can benchmark tool performances is unclear and needs better justification on the rationale.

2. The approaches and many conclusions are not rigorous. The description of the different results throughout Figures 2 and 3 is confusing as it is unclear whether the goal was to compare characteristics of the 2 technologies, to compare the qualities of the datasets, or to compare the performance of the tools. For example, the authors claimed that UV Rate is higher for HiChIP than ChIA-PET (Fig. 2). It seems to be an intrinsic characteristic of the technologies or the quality variations of the datasets. What conclusion can one make about method benchmarking from these? Also, their findings that HiChIP is more sensitive than ChIA-PET in capturing valid PETs, that HiChIP requires a substantially lower cellular input compared to ChIA-PET, and that HiChIP exhibits a significant restriction enzyme-bias compared to ChIA-PET, are all known and have been shown in previous publications (PMID: 27643841, PMID: 29489746).

3. Many conclusions drawn from the analysis are not supported by the data. For example, the authors claimed that "HiChIP has higher RE bias than ChIA-PET". But the data supporting this conclusion is not convincing enough. Again, is this a finding about the 2 technologies, the 2 different datasets, or the different tools?

4. Figure 3C is not convincing to show that HiChIP peaks are enriched for Mbol RE motif sites. Instead of this cumulative plot, the authors should show the count distribution of Mbol RE sites on all HiChIP peaks comparing others. The existing plot cannot rule out the possibility that Mbol motif may only be enriched on very few HiChIP peaks.

5. Lines 200-202, what do the authors mean by "HiChIP peaks were more distinct"? Fig. 3E and 3F only tells there are a lot of ChIA-PET specific peaks with low ChIP-seq signal (E) and a lot of ChIP-seq specific peaks with low HiChIP signal (F). The authors' interpretation was very insufficient and confusing.

6. Fig 3J, is it from one dataset or combining all datasets? If one, which one? If combined, what's the justification of combining results from different datasets together?

7. Lines 304-305 and Fig 5B, it does not seem to be a fair comparison from which the conclusion that cLoops outperforms CID was drawn. As the authors stated, CID produced more loops than cLoops, so it is possible that CID has high sensitivity is able to identify more true weak loops. Without a fair false-positive and false-native estimation, this conclusion does not hold.

8. What is the justification of using histone mark data and Hi-C data to validate loops? Conceptually, loop formation itself should be independent with histone marks. Hi-C is supposed to have lower resolution than ChIA-PET or HiChIP data. The identified loops should be validated by interaction-based methods or knockdown/knockout approaches.

9. The final conclusion and recommendations do not seem to be practically helpful to the audience or the community. The recommendations the authors can make from this study is very limited. The website describes what they did in this study, but does not provide any further information about the quality or justification of the selected published data. The authors did not provide a computational pipeline for the users to test their own new data or to benchmark any other new methods.

10. Calculations of most evaluation measures, including UV rate, PC, AA, and APA are application of standard procedures, with little methodological advances. Using established measures is not necessarily a bad thing, but the extensive lack of justification and over-interpretation of the data make the rational questionable.

Minor issues:

11. The authors mentioned they compared 12 methods designed for ChIA-PET and HiChIP data, but there are only 11 methods listed in figure4-6 and the website: CHIA-PET2, ChIA-PETv3, ChIApop, ChiaSig, CID, cLoops, FitHiChIP, HiC-Pro, HiChipper, mango, MAPS. Also, HiC-Pro is designed to process Hi-C data and I assume the authors only use HiC-Pro for data pre-processing. They should clearly explain what they refer to as those 12 methods.

12. SD is used as an abbreviation for both self-ligation and dangling-end reads (S+D) in Hichipper(SD), and super-enhancer domains (SDs). It is very confusing and the authors should make the use abbreviations with clarity and distinguishability.

13. On the Bacon website, there seems to be a mislabel/switch for the recommendation of tools for HiChIP and ChIA-PET data. Currently ChIA-PET tools are recommended for HiChIP data and HiChIP tools are recommended for ChIA-PET data.

**Authors Response**

**Point-by-point responses to the reviewers’ comments:**

Reviewer 1:

In this manuscript, Tang et al develop a computational benchmarking framework for comparing targeted chromatin conformation capture-specific methods. This is novel, to my knowledge. I am familiar with Forcato et al., Nature Methods, 2017, which compares computational methods for Hi-C, but this is done with Hi-C and not for methods such as Hi-ChIP and ChIA-PET. Overall, the paper is important to the field, as comparison of software is necessary for researchers to know which are the optimum tools for use. However, a major criticism that I have is that it is hard to know what are the "true" loops. Forcato et al., Nature Methods, 2017 overcame this challenge by using simulated data. Therefore, I am wondering if Tang et al can similarly develop simulated data for comparison between different software.

*Response: We thank the reviewer for the suggestion. To overcome the challenge of lacking “true” loops, we gathered long-range interactions from the ENCODE project, and then qualified a loop as “true” if it had eQTL and/or CRISPR/Cas9 perturbation validated contacts. These “true” loops we refer to as our gold standard loop set. We then evaluated the accuracy of different tools by comparing them against these gold standard loops. In addition, we developed 6 simulated datasets (3 ChIA-PET and 3 HiChIP) by integrating Hi-C and ChIP-seq data with a Poisson model to test the performance of different methods. The complete description of the experimentally-derived, and simulated loops is provided in the revised manuscript. Moreover, we have added a graphical representation of their use as shown in* ***Response Figure 1*** *(also see Figure 1 in the revised manuscript). The gold-standard loops were also incorporated into our calculations for accuracy (ACC), to help aid in the comparisons of the different software packages and processing pipelines.*

Additional comments

1.In the paper, besides mentioning the new HiChIP method, I think it is important to also cite the newer papers with newer and easier to use versions of ChIA-PET. So for this point "The recently developed HiChIP [18]and PLAC-seq [19] protocols reveal better resolution and require lower chromatin input than ChIA-PET." - I think is only valid when compared with the old, traditional ChIA-PET. I think the new ChIA-PET papers which have new long reads protocols should also be mentioned as they are also better in resolution and require lower chromatin input. In analysing the data and comparing methods, it would also be good to refer to data from these newer papers.

*Response: We agree with the reviewer that the long-read ChIA-PET protocol should be cited and we have added this to our revised manuscript. To ensure fairness when comparing methods, we selected traditional ChIA-PET and HiChIP datasets that were both derived from the same cell line and immunoprecipitated the same target protein. Unfortunately, the only publicly available long-read ChIA-PET data is from Tang et al. (2015), which doesn’t match any available HiChIP conditions. Thus, we were only able to compare long-read ChIA-PET data with standard ChIA-PET data. Overall, we addressed the reviewers concern by extending our benchmarking to long-read ChIA-PET data analysis through evaluating the performance of different analytical approaches on these datasets. Please see immediately below, Reviewer 1, point 2 for more details.*

2. I am also interested to see what are the recommendations like, and if they are different from the older, traditional ChIA-PET and the newer long ChIA-PET approaches.

*Response: We compared the differences between original and long-read ChIA-PET protocols, as well as tested the performance of different methods on these two types of datasets. Within all the traditional ChIA-PET analytical methods, only ChIA-PET2 (CPT2) and ChIA-PETv.3 (CPTv.3) supported the raw data analysis of long-read ChIA-PET data, thus we compared the performance of latest approach ChIA-PIPE [1] with CPT2 and CPTv.3. The results of these methods showed that the long-read protocols detected more uniquely valid PETs. In addition, all the methods achieved better results of peak occupancy (PC), accuracy (ACC), and active rate (AR) in the long-read protocol. Overall, ChIA-PIPE performed slightly better than CPT2 and CPTv.3 for both types of datasets (****Response Table 1****).*

*At the suggestion of the reviewer we have added* ***Response Table 1*** *to the Supplemental Material as* ***Supplemental Table S2****.*

3. Lines 86-88 "However, the differences between ChIA-PET and HiChIP protocols render many of the peak-based computational methods not applicable to both ChIA-PET and HiChIP data simultaneously." => I am interested to know what peak-based methods are not applicable. Is MACS2 not applicable? Or is it OK but others are not, and if so, why? What are these differences that complicate such analyses?

*Response: Given the strong bias of HiChIP data for restriction sites, most, if not all, peak-based methods are not applicable without modifications to the peak-calling parameters. MACS2 is incorporated by pipelines like Hichipper [2]. However, Hichipper solves the restriction enzyme (RE) bias issue by incorporating a background correction modelled after the RE-bias. These types of corrections lead to the generation of many small peaks that have to then be merged, and statistically present high p-values making their utility and quantification difficult [3].*

4. There are a lot of references to concepts such as loop anchors, splitting PETs, and so on. I think a glossary and/or schematic figures in the Supplement would greatly aid the readers in understanding these concepts.

*Response: We thank the reviewer for the suggestion. We have added schematic figure to better explain and visualize these terms and concepts (****Response Figure 2****).*

*At the suggestion of the reviewer we have added* ***Response Figure 2*** *to the Supplemental Material as* ***Supplemental Fig. S1****.*

5. The authors make reference to "popular Hi-ChIP-specific software package[s]" such as Hichipper. I was wondering if the authors could include a supplementary table or supplementary information comparing the various kinds of software, and also mentioning what are the papers that these methods have been used in? This will help to give the readers some idea of the comparisons between these different methods and their popularity. Also, the authors discuss the run time of the various methods, and a table comparing the run times of the different methods would be useful.

*Response: We thank the reviewer for their suggestion. We selected the most representative citation for each method. And counted the total citations for each method output by Google Scholar up to May 2021 (****Response Table 2****).*

*At the suggestion of the reviewer we have added* ***Response Table 2*** *to the Supplemental Material as* ***Supplemental table S3****.*

*The running time for each of the different methods was determined during the processing step. For ChIA-PET-specific analytical methods the alignment steps taken by different pipelines was often implemented by different genome aligners (like BWA, or Bowtie), so we only counted the running time of linker trimming, calling loops, and the detection of loop significance (****Response Table 3)****. And as most of the HiChIP methods didn’t allow running a single analysis step, we counted the total running time of calling loops, and detecting loop significance for HiChIP-specific pipelines (****Response Table 4****).*

*At the suggestion of the reviewer we have added Response Table 3 and 4 to the Supplemental Material as Supplemental table S4 and S5. We have also added information regarding the runtime of all the pipelines we evaluated to main Figure 6, so users can quickly visualize this information.*

6. In Figure 4, the authors compare some of the characteristics of the loops between the different software. I am curious to know what is the degree of similarity in loop calls between different methods? What loops are called differently by different methods, and is there any reason for the different loop calls? I think this will help to further guide the recommendations.

*Response: We agree with the reviewer that it’s important to address exactly what distinguishes the loops output from the various software packages here. To determine the degree of similarity between each peak-based method we used Principle Component decomposition to roughly compare the global differences between loop calls for H3K27ac-specific experiments. For ChIA-PET datasets, the loops called by four different peak-based methods (ChIAPoP, mango, CPT2 and CPTv.3) were similar, however, for these same datasets the two cluster-based methods produced loop outputs that were distinct from each other (Response Figure 3A). And the H3K27ac ChIP-seq peak intensity at anchors showed that loops output from ChIAPoP produced the highest signal near peak centers (Response Figure 3B-3C). For HiChIP datasets, we noticed that the loops detected by Hichipper with various parameters clustered together (Response Figure 3D), and that among those Hichipper(+chip) produced the highest H3K27ac ChIP-seq peak intensity compared to all other peak-based methodologies (****Response Figure 3E-3F****).*

*To investigate the differences between methods, we overlapped the optimal loops called by ChIAPoP with other ChIA-PET analytical methods individually. The results showed higher overlapping percentages between the three peak-based methods (ranges from 69.2% to 95.2%), compared to the two cluster-based methods (less than 10%) (****Response Figure 3G)****. For HiChIP analytical methods, we overlapped the optimal loops called by Hichipper(+chip) with other methods. We found that CID, a cluster-based method, achieved the highest overlapping percentage with the peak-based method Hichipper(+chip) (****Response Figure 3H)****.*

*To further investigate the properties of the different loops called by these methods, we chose ChIAPoP as the representative ChIA-PET peak-based method, and compared the ChIAPoP-specific loops, cLoops-specific loops, and CID-specific loops with active and inactive histone mark ChIP-seq data. The results showed that ChIAPoP produces more active chromatin-enriched peaks with higher H3K27ac signal than cLoops and CID, while cLoops and CID output loops with more inactive H3K27me3-enriched profiles (****Response Figure 4A****). This suggests that the peak-based methods outperform the cluster-based methods at detecting loops with active enhancer signatures. For the analysis of HiChIP, we chose Hichipper(+chip) as representative, and the comparisons showed similar results to what was observed for ChIA-PET. Overall, the peak-based methods detected more loops with H3K27ac enrichment than did the cluster-based methods. And the cluster-based methods detected more inactive or poised loops with heterochromatic H3K27me3 enrichment (****Response Figure 4B****).*

*The differences between each analysis pipeline that could be contributing to the differences in loop outputs are difficult to detail precisely, however, some of the factors contributing to the top performing pipelines are worth noting. For ChIA-PET analysis, ChIAPoP performed favorably and output loops with lower levels of H3K27me3. Loops with enrichment for H3K27me3, and a lack of H3K27ac are likely noise. And ChIAPoP was designed specifically to reduce the noise of ChIA-PET data. It does this by modeling sequencing depth, and genomic distance between anchors. Moreover, ChIAPoP incorporates chimeric reads into its model for noise correction, which cLoops and CID do not. Clearly, using the noise-associated chimeric reads in their underlying model produces beneficial increases in noise reduction over existing methods, and increases the output of loops associated with active chromatin regions. Future analytical pipelines should work to incorporate this chimeric read information, instead of discarding it at after pre-processing. Hichipper(+chip) appeared to outperform other analysis pipelines at detecting loops with enhancer signatures, and diminishing noise. As Hichipper(+chip) incorporates ChIP-seq peaks as input to call loops, it effectively eliminates the restriction enzyme bias associated with HiChIP peak calling. Cluster-based methods call loops using PET-enrichment, independent of peak regions, and likely contributes to the enhanced noise associated with these methods. However, both cluster-based methods were able to detect bivalent regions with co-occurring H3K27ac and H3K27me3 loops. These bivalent-associated loops are presumably poised regulatory elements. While the functionality of these loops is unknown and their enrichment minimal, it is notable that the cluster-based methods offer a different level of sensitivity towards these regions.*

*At the suggestion of the reviewer we have added Response Figure 3 and Response Figure 4 to the Supplemental Material as Supplemental Fig. S8 and S9.*

7. In the discussion, the authors said "Overall, improved computational methods are urgently needed to effectively detect chromatin interactions from these datasets" but did not give any suggestions for what should be improved in each method. Based on Bacon, can the authors identify some points where improvements can be made? Would running two different methods and comparing the data between them help to improve the quality of loops called?

*Response: The reviewer raises a good point. Namely, that it would be helpful to guide the reader through the optimal method selection. To address this point, we have now added a practical guide for the users and we have provided suggestions to developers (****Response Figure 5****).*

*At the suggestion of the reviewer we have added* ***Response Figure 5*** *as* ***Figure 6*** *to the revised manuscript, and the following to the discussion:*

*“To provide practical guidance for users, we considered different conditions, analytic strategies, and the performance of each tool (Fig. 6). The ACC/AR generated by different datasets were re-scaled from 0-1, and then annotated based on how well they performed across multiple data sets. We also recorded the running time for each loop calling method across different datasets. For ChIA-PET analysis, ChIAPoP outperformed the others in accuracy and detecting activation, however, ChIAPoP required more running time than the other peak-based methods, and can’t be applied to datasets generated by long-read ChIA-PET protocols. For the HiChIP analytical methods, Hichipper with the reference ChIP-seq peaks as input performed best in ACC, AR, and running time. And if no ChIP-seq peaks are available to provide to Hichipper, then Hichipper(C+S) was the best alternative. However, Hichipper only accepts the valid pairs output from HiC-Pro to call loops, so if users want to perform the analysis procedure without switching methods, then MAPS represents the only choice. We noticed that although cluster-based methods (CID and cLoops) can be applied to both ChIA-PET and HiChIP datasets, the ACC and AR metrics didn’t stand out, and these two methods also took more running time than the peak-based methods.*

*Here we provided several suggestions for the future development analytical pipelines for both HiChIP and ChIA-PET analysis. Importantly, more flexible parameters should be considered, such that future chemistries, sequencing read lengths, and other experiment-specific factors can be appropriately accounted for. Importantly, we found that the methods we analyzed integrated different alignment tools, and the key mapping parameters were fixed by most pipelines. However, for different lengths of raw sequencing data, the alignment settings should be adjustable to achieve optimal results. Additionally, more reasonable self-ligation cutoffs should be set, such that the reads being input for loop calling are completely valid. Self-ligation PETs are filtered out prior to calling loops, and the cutoff between a read PET being designated as a self-ligation product versus inter-ligation product ranges from 5 kb to 12 kb [4,5]. While most methods simply set the cutoff as a fixed value or asked users to set it themselves, the cutoff should be defined in a more rational way, such as being based on the distribution model of PET lengths.*

*Finally, we believe that the next generation of computational tools for analyzing protein-directed 3D chromatin topology should boost sensitivity to account for weak or dynamic interactions. Inherited from the processing pipelines of ChIP-based techniques, peak calling was the most frequently used strategy to detect the enrichment of PETs. Since peak calling results rely highly on ChIP enrichment, some weak or dynamic interactions are likely to go undetected [6]. Cluster-based methods rely on the relative read densities rather than the absolute values, making more locally-enriched loops standout compared to peak-based methodologies. Thus, cluster-based methods offer a viable solution to detect more dynamic topological interactions. However, this sensitivity comes at the expense accuracy. How to balance the detection of dynamic loops while maintaining the accurate detection of strong loops remains a challenge which needs to be addressed. A rational approach to the development of such an analytical pipeline would be a mixed model, where the accuracy of the top-performing peak-based approaches and the sensitivity of the cluster-based pipelines are combined. A number of considerations have to be made for such a mixed model, but ultimately it needs to limit noise, boost signal, and output an accurate representation of the factor-enriched 3D genome. Firstly, noise has to be reduced. Both ChIAPoP and Hichipper(+chip) reduced noise more effectively than other pipelines. ChIAPoP cleverly incorporates into their model the use of chimeric reads, in addition to sequencing depth and inter-anchor distance, for estimating noise. And the addition of ChIP-seq data to Hichipper also dramatically improves noise reduction. Both strategies incorporated together within a statistical framework could theoretically aid in the noise reduction necessary for the generation of a mixed peak- and cluster-based model.”*

Minor comments

8. On https://csuligroup.com/Bacon/Recommendation, under recommendations, "Hichipper(ref)" is listed. Is "ref" supposed to be there? You can also see this in the paper at line 353.

*Response: The Hichipper(Ref) represented using reference ChIP-seq peak as input to call loops in Hichipper method. For easier understanding, we have changed Hichipper(Ref) to Hichipper(+chip).*

9. The manuscript is quite understandable, but still has some grammar errors. Another round of proof-reading would be helpful.

*Response: We are sorry for the trouble. In our revised manuscript, we have performed a carefully review to remove any grammatical errors.*

10. Lines 54-55 "Chromatin interaction analysis by paired-end tag sequencing (ChIA-PET) is a technique which combines ChIP and 3C" => Technically, it is a combination of ChIP, 3C and next-gen sequencing. The combination of ChIP and 3C is ChIP-3C.

*Response: Thanks for the comment, we have modified this sentence in the introduction.*

Reviewer #2:

In this paper, the authors presented a comprehensive benchmark framework, Bacon, to evaluate the performance of 12 computational methods (but only 10 showed in the workflow), and provide practical recommendations for users working with HiChIP and/or ChIA-PET analysis. The detected significant loops were validated by epigenomic features and published Hi-C profiles. The evaluation results indicated that no single method outperforms the others in every aspect, and that improved computational methods are needed to effectively detect chromatin interactions. Indeed, it is very disappointing that there is no good suggestion for choosing a tool. I have several serious concerns about the work.

Major:

1. It is not necessary to include "preprocessing" step in the evaluation since the data quality is from experimental parts and can be QC using many other mapping tools.

*Response: Thanks for the comment, we agree with the reviewer that “preprocessing” steps can be QC using mapping tools. However, within the pre-processing steps there is a considerable degree of variance, especially for many ChIA-PET pipelines which perform pre-processing and loop calling in a single workflow, including different linker trimming strategies and mapping tools (****Response Table 5****). Firstly, since there was no clear description for the linker mismatch of mango (fix setting), we tried 0 and 1 mismatch for ChIAPoP and ChIA-PET2, and ChIA-PETv.3. The results showed that the mismatch setting caused a small, but detectable, impact on UV Rate. Although we set the same MAPQ filtering threshold for different methods, the different fixed settings inside some pipelines caused the UV Rate to vary considerably. Thus, we believe pre-processing is a necessary step to consider for the overall evaluation.*

***Response Table 5. Comparison of pre-processing strategies***

| ***Category*** | ***Methods*** | ***Mismatch in linker*** | ***Mapping tools*** |
| --- | --- | --- | --- |
| *ChIA-PET* | *ChIAPoP* | *Allow to set* | *Bowtie* |
|  | *Mango* | *Fix setting* | *Bowtie* |
|  | *ChIA-PET2* | *Allow to set* | *BWA* |
|  | *ChIA-PETv.3* | *Defined linker alignment score* | *BWA* |
| *HiChIP* | *HiC-Pro* | *—* | *Bowtie2* |
|  | *MAPS* | *—* | *BWA* |

*Also see below Reviewer 2, Response 6.*

2. The better way to evaluate the tools with biological replicates. Since both HiChIP and ChIA-PET have biological replicates, the authors should examine the reproducibility of binding peaks and called loops from biological replicates by each of 12 tools.

*Response: We agree that the reproducibility of biological replicates is a good evaluation metric to compare these various methods. We have taken the reproducibility analysis for different methods, we firstly combined the loops of different replicates by each tool, the number of combined loops for tool i was defined as N_i_, then we intersected the loops of different replicates, the number of loops called by all replicates was defined as n_i_, the reproducibility of tool i was defined as , the reproducibility results showed that Hichipper tool with “C+S” and “C+A” modes achieved the best reproducibility in all HiChIP datasets, followed by Hichipper(+chip)*

*We have added to* ***Response Figure 6 to*** *Supplemental Material as* ***Supplemental Fig. S13.***

3. For Peak Co-occupancy (PC), the authors should clarify the novelty of PC compared to the results from diffBind, as both methods tend to address how many 'shared peaks/diff peaks' between ChIP-seq peaks and ChIA-PET/HiChIP peaks. For me, the diffBind used a reliable statistical inference to identify the differentiated peaks but PC just simply found 1bp overlapped peaks between ChIP-seq peaks and ChIA-PET/HiChIP peaks.

*Response: We agree that diffBind is a powerful tool for the differential analysis of ChIP-seq binding sites. Indeed, diffBind identifies statistically significant differentially bound sites based on read densities, and uses statistical routines developed for RNA-Seq differential expression (DeSeq2 and edgeR). While the motivation of PC is to compare the peak occupancy of loop anchors. The input for PC requires only loops and ChIP-seq peaks, so users can evaluate the reliability of their own loop anchors without providing the large bam files necessary to run diffBind. To improve the algorithm design of PC, we considered more elements to reconstruct PC.*

*To address the reviewers concerns we added following to the methods section of revised manuscript:*

*“The uniquely mapped alignment files of ChIA-PET and HiChIP were obtained in the preprocessing step of Bacon, which were used to call loops by different computational methods. Bacon extracted the anchors from called loops, then the anchors with a minimum 90% of length overlapped with each other were merged. The publicly available ChIP-seq peaks were then used as target sets to detect overlaps with different anchor sets. All the anchors that had at least 1 bp overlap with a ChIP-seq peak were gathered into a candidate set (A). The total number of anchors in the loop set was represented by N, and the number of anchors in A was represented by N_A_. The length of anchor i was represented by The peaks that overlapped with anchor i was Pi, and the length of this peak was . The overlapping length between anchor i and the ChIP-seq peak was represented by . If there were more than one peak overlapped with the anchor, the peak with the longest overlapping length was selected. The PC was calculated as follows:*

**PC=(N_A*∑_(i=1)^N▒max⁡(L(o_i )/L(p_i ) ,L(o_i )/L(a_i ) ) )/N**

4. For Enrichment Score (ES), the authors assumed that 'the true enriched loci should be surrounded by relative low enriched PETs' before calculating the ES. Thus, the authors should verify their assumption either from previous literatures or from the data they used.

*Response: We thank the reviewer for the suggestion. The assumption was based on the thought that the cluster center should be recognized as the read density maximum point locally, which is far away from any higher density points. This type of analysis only relies on the relative densities rather than the absolute values. The assumption was verified in the following previous study [7].*

*We have added the following to discussion:*

*“Cluster-based methods rely on the relative read densities rather than the absolute values, making more locally-enriched loops standout compared to peak-based methodologies. Thus, cluster-based methods offer a viable solution to detect more dynamic topological interactions [6,7]”*

5. The authors should explain how to determine the value of ∝ in calculating global ES with low sequencing depth.

*Response: We thank the reviewer for the suggestion, we have added the following to the methods section of the revised manuscript:*

*“∝ was a coefficient to adjust the enrichment of low PET coverage regions caused by uneven sequencing depth. The PET coverage of the whole genome was calculated by C=LN/G, C stood for coverage, G is the length of genome, L is the length of PET, N is the number of PETs, Cj is the PET coverage of j chromosome, was calculated by .”*

6. In P10L172, the authors claimed that MAPS performed better than HiC-Pro based on the result that unique valid rate of MAPS is higher than HiC-Pro's. However, as these two programs only performed read alignment and PETs filtering in the framework, the author should make sure that both programs used similar level of mapping and filtering with their parameters and clarify which mapping parameters or filters cause this big difference. Otherwise, the high UV rate might result from a more tolerant mapped setting or loose filtering process. The same concerns also in the UV rate in the ChIA-PET.

*Response: For the pre-processing step, we set the minimum mapping rate (MAPQ) to 30, mismatch 0 and 1 were set for the linker trimming of CPT2 and ChIAPoP, mango was fixed to default settings, and CPTv.3 used the default linker alignment score.*

*For the filtering of HiChIP data, MAPS removes low-quality alignments, unmapped PETs, multiple-alignments, and PCR duplications. While HiC-Pro was developed to process Hi-C data, which proposed a two-step alignment, the first step removed the same parts as MAPS, the second step removed dumped pairs, dangling ends, and self-circles, making the total number of unique valid PETs output from HiC-Pro fewer than MAPS.*

*For the ChIA-PET data, we noticed that the number of mismatches allowed during linker trimming indeed impacted the UV Rate, however, the impact was small. The greatest difference was caused by the key parameters passed on to the genome alignment tools. For the development of ChIA-PET analytical methods, the easiest way to perform alignment was by integrating published aligners (like Bowtie, and BWA). Many of the ChIA-PET analytical methods fix key parameters of alignment tools, such as CPT2 which integrated BWA to perform alignment, and provides only two optional modes: “bwa aln” (for reads around 20bp) and “bwa mem” (for reads >70bp). CPTv.3 also integrates BWA to perform mapping, and utiluzes fixed parameters “bwa aln” and “bwa samse”. To make these differences known to the users we added* ***Response Table 5*** *to Supplemental Material as* ***Supplemental table S1.***

7. More analyses and visualizations should be performed to comprehensively tell the difference between programs, such as the distribution of the loop length from different programs; the peak intensity from different peak-based programs; histone modification signal on the anchors; visualizing identified loops from different programs on the Hi-C matrix.

*Response: We have added the comparison for the distribution of loop distance (length), which displays the distribution of loops detected by the different tools. The loop distances of ChIA-PET data ranged from 10 kb to 100 kb, and the loops from HiChIP data range up to 1 Mb (****Response Figure 7****).*

*We have also added the peak intensity comparison for different methods, which showed that ChIAPoP and Hichipper(+chip) display the highest peak intensity within ChIA-PET and HiChIP analytical methods, respectively (****Response Figure 3B-3F****). For the chromatin mark composition of different loops, we have incorporated the candidate enhancer-like and promoter-like signatures output from ENCODE [8] to annotate the loops, as well as histone mark ChIP-seq data for H3K27ac, H3K4me1, H3K4me3, and H3K27me3. We also used these histone marks to calculate the Activation Rate (AR) of enhancer-mediated loops. The AR results showed that ChIAPoP and Hichipper(+chip) detected the greatest number of active Enhancer-Promoter loops (****Response Figure 8A-8B****). To further investigate the activation differences between peak-based and cluster-based methods, we also added the comparison of histone marks between these two types of loop anchors (above,* ***Response Figure 4****).*

*We agree that displaying the Hi-C matrix is suitable to visualize Hi-C data; however, for the visualization of loops smaller than 1 Mb, we believe arcs display the chromatin interactions more clearly. We added two representative examples for illustration to the updated* ***Figure 5****. Firstly, we interrogated the MYC locus with K562-H3K27ac ChIA-PET data, and we found that most methods detected contacts which overlapped with a set of previously validated MYC enhancers [9]. Notably, ChIAPoP loops contacted more CRISPRi MYC enhancers than the other methods, such as MYC-enhancer5, MYC-enhancer6, and MYC-enhancer7 (****Response Figure 8E)****. Secondly, we evaluated K562-H3K27ac HiChIP data against another set of CRISPRi validated loops [10]****, and*** *we found that there were four methods detecting loops near the SEMA7A locus, however, only Hichipper(+chip) identified loops between this CRIPSRi-validated distal enhancer and the SEMA7A promoter region (****Response Figure 8F****).*

*We have added* ***Response Figure 8*** *to revised manuscript as* ***Figure 5.***

8. Using epigenomic data and Hi-C data as validations is not a bad idea. However, the authors oversimplified it with a simple correlation of E-P looping vs enhancer marks. The authors should come up a more systematic way to validate the loops and binding peaks.

*Response: We agree with the reviewer that our incorporation of Hi-C data, our enhancer-directed comparisons are not effective for evaluating the functional presence of loops. To more rigorously assess the function of the loops output from the various methods we benchmarked, we overlapped the detected loops with large-scale CRISPR/Cas9-validated perturbation screening data performed in K562 cells [9,10]. We found that ChIAPoP and Hichipper(+chip) achieved the highest overlapping percentage in ChIA-PET and HiChIP datasets (Response Figure 8C-8D), respectively, which was identical to the results of the ACC output (Response Figure 12). As mentioned above, we also added two examples of enhancer-gene interactions for illustration, highlighting some of these putatively functional topological interactions (Response Figure 8E-8F).*

9. HiChIP-Peaks should be included in the comparison.

*Response: We have systematically compared HiChIP peaks, ChIA-PET peaks, and ChIP-seq peaks. Firstly, Principal Component Analysis (PCA) showed that the ChIP-seq, ChIA-PET, and HiChIP replicates clustered together into their respective groups. Next, we also compared the restriction enzyme bias of ChIA-PET and HiChIP in a quantitative manner, and called peaks for ChIP-seq, ChIA-PET and HiChIP datasets separately. Finally, we calculated the distribution of Mbol restriction enzyme motifs near peaks. We found a similar occupancy profile between ChIP-seq and ChIA-PET peaks, while HiChIP exhibited a higher occupancy with Mbol restriction enzyme sites (see Figure 2 in revised manuscript).*

10. Figure 2B provides the same information in Figure 2A.

*Response: Thanks for the suggestion, we have removed the original Figure 2A in the revised manuscript.*

11. P18L289, H3K27ac peak is narrow-peak that recognize by ENCODE (https://www.encodeproject.org/chip-seq/histone/) and author should double-check the statement in the paper their cited.

*Response: We thank the reviewer for pointing this out. We have corrected it in the revised manuscript.*

Minor:

12. Is there any particular reason to choose mismatch 1? Since the larger mismatch allowed, the more reads will map to the reference genome. The result of mismatch 1 doesn't give any new information.

*Response: We agree that the larger mismatch allowed, the more reads will map to the reference genome. Please see response above to Reviewer-2, points 1 and 6.*

13. Figure. 3C is hard to understand, why are there two HiChIP peaks flank the Mbol Res. Motif?

*Response: During the HiChIP protocol MboI digested ends are ligated together and these sites are tagged with biotin. Streptavidin beads are later used to enrich for these fragments that contain ligated junctions. Hence, these reads contain MboI restriction sites at their interior. The sequenced fragments are at the up- and down-stream portions of the fragment. These sequenced fragments, that are undigested by MboI, contribute to the anchors which pileup to form peaks. Hence, the peak centers lack MboI sites while the adjacent sequences of the fragment contain high levels of MboI sites, as these were enriched during the biotin pulldown. See diagram below for clarification (Response Figure 9).*

14. MAPS uses feather to do the mapping part. It would be better to annotate MAPS with feather in Figure 1.

*Response: Thanks for the comment, “feather” was a name given by MAPS to describe the step of mapping and pre-processing, which was similar as the other methods, and the mapping part of MAPS was implemented by BWA. To avoid misunderstanding, we have removed the all the annotation of methods in Figure 1.*

15. Figure 5B should be a joint plot instead of a Hexbin plot.

*Response: Thanks for the comment, we have modified “Hexbin plot” to “joint plot”.*

16. The authors should make the term consistent, e.g. P19L317 'Supplemental Figure. 5C' and P19L318 'Fig S5C'

*Response: Thanks for the suggestion, we have uniformed the figure legends.*

17. There are too many typos in the manuscript, e. g. P21L338 uses the pass tense but P21L339 uses the present tense. The authors should carefully check the English grammar for all of the paper.

*Response: Thanks for the suggestion, we have carefully checked the typos and modified the English grammar.*

Reviewer #3:

This manuscript describes a computational work to evaluate 12 analytical tools for two specific types of 3D genome profiling data, ChIA-PET and HiChIP. The authors compare the results from the tools on two datasets from each technique, one mouse cell type and one human cell line, respectively. The evaluation results indicate that no single method is superior. The manuscript is written clearly and straightforward to follow. While systematic comparison is important in the field and the data shown in this manuscript may be of interest to some researchers, I have some concerns on the design, approach, scientific rigor, and significance of this work. Specifically,

Major issues:

1. There are fundamental flaws on the study design. A benchmarking study comparing different methods should have uniform gold (or silver) standards and comprehensive evaluation criteria. It is not sufficient to simply compare results of the tools on 2 datasets. There are plenty of datasets generated from ChIA-PET and HiChIP, why the authors only chose these 2 particular datasets, and why these two datasets can be used for benchmarking, are completely unjustified. What if the results only reflect the quality difference on these 2 datasets? The quality of the benchmarking data should be thoroughly assessed before using them for benchmarking other tools. Besides, different results from different tools on data generated using different technologies are expected. How the different results can benchmark tool performances is unclear and needs better justification on the rationale.

*Response: We thank the reviewer for their comment. Please first see above; Reviewer 1, response 1. And to compare the different characteristics between ChIA-PET and HiChIP data, we chose studies conducted on the same cell type and with the same antibody for ChIA-PET and HiChIP, which is why we used two K562-H3K27ac and two mES-SMC1 datasets for esting. We agree that more datasets are needed to generate a more comprehensive evaluation, so we have added K562-CTCF, GM12878-RAD21 of ChIA-PET, YY1, and H3K27ac HiChIP from the GM12878 cell line for testing. In addition, 6 simulation datasets were generated by integrating Hi-C and ChIP-seq data with a Poisson model to test the performance of different methods, as mentioned above. The statistics and quality of all the testing datasets are summarized in Response Table 6-7.*

*We have added* ***Response Table 6-7*** *to* ***Supplemental Table 1,*** *all the datasets are available at* [*https://csuligroup.com/Bacon/Home*](https://csuligroup.com/Bacon/Home)*.*

2. The approaches and many conclusions are not rigorous. The description of the different results throughout Figures 2 and 3 is confusing as it is unclear whether the goal was to compare characteristics of the 2 technologies, to compare the qualities of the datasets, or to compare the performance of the tools. For example, the authors claimed that UV Rate is higher for HiChIP than ChIA-PET (Fig. 2). It seems to be an intrinsic characteristic of the technologies or the quality variations of the datasets. What conclusion can one make about method benchmarking from these? Also, their findings that HiChIP is more sensitive than ChIA-PET in capturing valid PETs, that HiChIP requires a substantially lower cellular input compared to ChIA-PET, and that HiChIP exhibits a significant restriction enzyme-bias compared to ChIA-PET, are all known and have been shown in previous publications (PMID: 27643841, PMID: 29489746).

*Response: To clarify Figures 2 and 3, we have completely reorganized the data. Figure 2A has been removed (see reviewer 2, response 10). And Figure 2B, has been merged with the previous Figure 3. See below, Response Figure 9, for the configuration of the updated Figure 2. Further, we have greatly simplified the results section to clarify what is being shown.*

*The motivation was to construct a comprehensive evaluation framework for targeted chromatin conformation capture-specific methods. HiChIP is a derivative technology of ChIA-PET, and both technologies are widely used in the genomics community, thus we felt it beneficial to include both. We performed a side-by-side comparison of the two different experimental protocols to understand how the subtle differences in experimental procedures influence the datasets which are produced. And while many comparisons have been done on ChIA-PET and Hi-C, very few have systematically evaluated HiChIP and ChIA-PET head-to-head, to the best of our knowledge. Thus, we believe it is necessary to include this comparison to further understand the data structures being input into the tools we are evaluating, in an effort to tease apart how they perform given the pros and cons for each protocol. And to understand how each tool solves or mitigates the biases associated with the input data, we really wanted to have and to present those biases explicitly and in thorough detail. Overall, the comparison between data characteristics aided the evaluation of the computational methods.*

*Many researchers treat the two, HiChIP and ChIA-PET, as equivalent strategies or use the loops derived from each method to present evidence which supports the same conclusions. For example, a distal regulatory element interacts with a gene of interest. Our results suggest otherwise, that the two methods should not be seen as equivalent, despite a fair degree of concordance. As the reviewer has noted, while HiChIP generates substantially more loops it also produces more noise. Moreover, HiChIP peaks don’t overlap as well with ChIP-seq peaks as do ChIA-PET peaks. This disagreement of HiChIP data with ChIP-seq is especially important for researchers looking at transcription factors to be aware of when designing their experiments and selecting an analytical tool.*

*We agree that some of the findings have been shown in previous publications, and we have acknowledged this in our updated manuscript. However, the restriction bias was only covered by a single figure in a short correspondence-style publication, thus we believe it is justified to include this analysis in our manuscript.*

*To address these concerns, we have re-organized* ***Response Figure 10*** *as* ***Figure 2*** *in the revised manuscript. And we have updated the results for this figure to read as follows:*

3. Many conclusions drawn from the analysis are not supported by the data. For example, the authors claimed that "HiChIP has higher RE bias than ChIA-PET". But the data supporting this conclusion is not convincing enough. Again, is this a finding about the 2 technologies, the 2 different datasets, or the different tools?

*Response: We re-organized the results, and added more clarifications to make this clearer. Please see Reviewer 3, point 2. For the RE bias comparison between HiChIP and ChIA-PET data, we have added* ***Response Figure 11*** *to support the conclusion, please see immediately below for more details. Also, you acknowledged above that this point has been touched on in the literature (PMID: 29489746).*

*This finding is a result of the technology, which we feel many users overlook when presenting these types of data. HiChIP technology, like Hi-C, utilizes restriction enzymes to fragment the genome prior to proximity ligation and biotin labelling. ChIA-PET utilizes sonication to fragment the genome, and then linkers are inserted during the proximity ligations with specially designed and highly-specific MmeI restriction enzyme sites. These sites within the linker allow the enzyme to cut ~20 bp outside of the linker, which is why traditional ChIA-PET requires such short reads. Hence the restriction digested utilized for ChIA-PET is only utilized by design to enrich for ligation sites, whereas, for HiChIP the restriction enzyme sites dictate where the proximity ligation occurs. As you mentioned above, we are not the first to detail this bias [2]. However, we feel strongly that it needs to be addressed up front for all users to be aware of, and for all future software developers to consider strongly. Moreover, some models, e.g. hichipper, correct for this bias while others (CID, cLoops) do not.*

4. Figure 3C is not convincing to show that HiChIP peaks are enriched for Mbol RE motif sites. Instead of this cumulative plot, the authors should show the count distribution of Mbol RE sites on all HiChIP peaks comparing others. The existing plot cannot rule out the possibility that Mbol motif may only be enriched on very few HiChIP peaks.

*Response: Thanks for the suggestion, we have analyzed the count distribution of Mbol RE sites on all HiChIP/ChIP-seq/ChIA-PET peaks, the results show that more than 60% of ChIP-seq/ChIA-PET peaks are without Mbol restriction enzyme sites, while most HiChIP peaks had 5-10 enzyme sites (****Response Figure 11****). Also, it’s important to note that most of this enrichment is adjacent to peaks, not at the peak center, see above reviewer 2, point 13.*

*We have added* ***Response Figure 11*** *to the Supplemental material as* ***Supplemental Fig.S3B.***

6. Fig 3J, is it from one dataset or combining all datasets? If one, which one? If combined, what's the justification of combining results from different datasets together?

*Response: The figure was from a single mES-SMC1 HiChIP dataset, limited by the length of manuscript, we have moved* ***Fig. 3G-3J*** *to supplemental material as* ***Supplemental Fig. S4****, and added clarification to the figure legend.*

7. Lines 304-305 and Fig 5B, it does not seem to be a fair comparison from which the conclusion that cLoops outperforms CID was drawn. As the authors stated, CID produced more loops than cLoops, so it is possible that CID has high sensitivity is able to identify more true-weak loops. Without a fair false-positive and false-native estimation, this conclusion does not hold.

*Response: The global Enrichment Score was proposed to evaluate the enrichment level of cluster-based loops by PET coverage, which estimated the average enrichment state of loops across the genome. Indeed, CID produced more loops than cLoops, however, the average enrichment of loops was lower than cLoops.*

*We also estimated the true positive, false positive, true negative, and false negative loops by intersection with our gold standard loop sets (see above), and then calculated the accuracy (ACC) of detected loops. The ACC results showed that cLoops outperformed CID, which was identical to the estimation of Enrichment Score (****Response Figure 12****).*

*We have added* ***Response Figure 11*** *to the revised manuscript as* ***Figure 4****, and we also added the following to results:*

*“****Evaluating the accuracy of loops***

*To evaluate the loops generated by different methods in a quantitative manner, we gathered cell type-specific long-range contacts from our gold standard loop sets. To ensure the fairness of comparison, we generated three gold standard loop sets for each testing dataset with different significance thresholds (for the gathering of gold standard loop set, see methods). Accuracy (ACC) was calculated for True Positive (TP), False Positive (FP), True Negative (TN) and False Negative (FN) metrics (for the calculation of ACC, see methods).*

*Although there were more HiChIP loops than ChIA-PET loops (****Fig. 4A****), the ACC of loops was independent of the number of loops (****Supplemental Fig. S10A****). The ACC of ChIA-PET loops was higher than that of HiChIP loops across the 8 testing datasets (****Supplemental Fig. S10B****). To investigate what impacted the results of ACC, we simulated ChIA-PET and HiChIP datasets with a Poisson distribution model and high-quality ChIP-seq data (see methods). The evaluation results of the simulation datasets showed that a positive correlation of ACC with ChIP-seq enrichment (****Supplemental Fig. S10C****). Next, we calculated the Pearson’s correlation coefficient for ACC and three other evaluation metrics. The results suggested that UV Rate barely correlated with ACC (****Supplemental Fig. S10D****), while PC and ES were positively correlated with ACC (****Supplemental Fig. S10E*** *and* ***S10F****).*

*We then compared the ACC of different methods separately. For ChIA-PET datasets, ChIAPoP achieved the best with K562-H3K27ac and mES-SMC1 datasets, and CPTv.3 achieved the best with GM12878-RAD21 data. For K562-CTCF data, the cluster-based method cLoops achieved the highest ACC. The PC of K562-CTCF was significantly lower than the other three datasets, while the ES of K562-CTCF had no obvious distinction (****Supplemental Fig. S10G*** *and* ***S10H****). These results indicated that the PC of ChIA-PET datasets fluctuated according to the ChIP enrichment, while the ES was less impacted by this parameter. Generally, when the PC of ChIA-PET datasets was over 0.65, ChIAPoP or CPTv.3 were good choices, however, if the PC was too low, the cluster-based method cLoops performed better. For the HiChIP datasets, Hichipper(+chip) output the highest ACC in all datasets.*

8. What is the justification of using histone mark data and Hi-C data to validate loops? Conceptually, loop formation itself should be independent with histone marks. Hi-C is supposed to have lower resolution than ChIA-PET or HiChIP data. The identified loops should be validated by interaction-based methods or knockdown/knockout approaches.

*Response: We agree that this is not appropriately described as validation. And we have changed the phrasing from “validation” to “functionality”. For the validation of significant loops, please see above response to reviewer 2, point 7.*

*We assessed histone marks to evaluate the status of loop anchors, which is mainly useful when evaluating ChIA-PET and HiChIP data targeting histone marks. The reviewer is correct that this same analysis may not hold true for transcription factors that bind throughout the genome independently histone modifications. Mainly, for histone mark-directed 3D datasets this allows us to annotate these loops, and determine if they are likely to reside in active or inactive regulatory regions. We performed additional analysis to clarify this point. The results revealed that the E-P loop percentage of mES-SMC1, a structural component of cohesion, was lower than that of H3K27ac- and YY1-targeted datasets (Response Figure 13A). Which can be explained by the fact that H3K27ac is an active enhancer- and promoter- associated histone mark, and YY1 binds to active enhancers and promoter–proximal elements [11,12]. In addition, the AR results indicated there were more activated Enhancer-mediated loops in H3K27ac- and YY1-targeted datasets (Response Figure 13B). We also quantified the percentage of E-P loops output from each method, and we found that some methods performed better than others (Response Figure 13C-13D). Interestingly, despite its high performance in most other metrics, ChIAPoP output loops with a lower percentage of E-P annotations compared to other ChIA-PET-specific methodologies.*

*We have added Response Figure 13 as Supplemental Fig. S11 to Supplemental Material.*

9. The final conclusion and recommendations do not seem to be practically helpful to the audience or the community. The recommendations the authors can make from this study is very limited. The website describes what they did in this study, but does not provide any further information about the quality or justification of the selected published data. The authors did not provide a computational pipeline for the users to test their own new data or to benchmark any other new methods.

*Response: Thanks for the comment. We have added more testing datasets, re-constructed the framework of Bacon, and re-analyzed the results to provide a practical guidance for users. Moreover, we have added additional recommendations to future developers based based on our results. Please see reviewer 1, point 7.*

*We have also added more clarifications regarding the datasets used in this study, recommendation guidelines, and all the scripts to the Bacon website. The computational pipeline of Bacon has been submitted to GitHub page (*[*https://github.com/CSUBioGroup/Bacon)*](https://github.com/CSUBioGroup/Bacon))*.*

10. Calculations of most evaluation measures, including UV rate, PC, AA, and APA are application of standard procedures, with little methodological advances. Using established measures is not necessarily a bad thing, but the extensive lack of justification and over-interpretation of the data make the rational questionable.

*Response: We have toned down our responses in the results to avoid over-interpretation of our results.*

*In the study of Mumbach et al. [13], the percentage of uniquely mapped valid PETs was used to evaluate the efficiency of HiChIP and ChIA-PET data, here we defined this metric as UV Rate to estimate the quality of datasets as well as evaluate the pre-processing step of different tools.*

*To demonstrate the co-occupancy between loop anchors and ChIP-seq peaks, Lareau et al. [2] analyzed the cumulated peak density near loop anchors. In our study, we combined peak density and the length of anchors to calculate PC, which evaluated the reliability of loop anchors in a quantitative manner. In the study of Hnisz et al. [14], histone marks of H3K27ac and H3K27me3 were used to aid understand the activity of chromatin interactions, here we proposed AR to quantify the activity of loops with the ChIP-seq signals of H3K27ac, H3K4me1, H3K4me3 and H3K27me.*

*APA was proposed by Rao et al [15] to quantify how well the Hi-C data supported the ChIA-PET interactions, which was commonly used to compare the performance of different tools [5,16,17], thus, we utilized APA as one of the evaluation metrics.*

Minor issues:

11. The authors mentioned they compared 12 methods designed for ChIA-PET and HiChIP data, but there are only 11 methods listed in figure4-6 and the website: CHIA-PET2, ChIA-PETv3, ChIApop, ChiaSig, CID, cLoops, FitHiChIP, HiC-Pro, HiChipper, mango, MAPS. Also, HiC-Pro is designed to process Hi-C data and I assume the authors only use HiC-Pro for data pre-processing. They should clearly explain what they refer to as those 12 methods.

*Response: We thank the comment. HiC-Pro was used to pre-process the HiChIP data for Hichipper and FitHiChIP, the 12 methods were: CHIA-PET2, mango, ChIA-PETv3, ChIApop, ChiaSig, MICC, CID, cLoops, FitHiChIP, HiChipper, MAPS, HiCCUPS.*

12. SD is used as an abbreviation for both self-ligation and dangling-end reads (S+D) in Hichipper(SD), and super-enhancer domains (SDs). It is very confusing and the authors should make the use abbreviations with clarity and distinguishability.

*Response: Thanks for the suggestion, we have modified the Hichipper(SD) as Hichipper(S+D) in the revised paper, and removed the abbreviation of super-enhancer domains.*

13. On the Bacon website, there seems to be a mislabel/switch for the recommendation of tools for HiChIP and ChIA-PET data. Currently ChIA-PET tools are recommended for HiChIP data and HiChIP tools are recommended for ChIA-PET data.

*Response: Thanks for the suggestion, we have checked the website and fixed all the errors.*

*References*

*1. Lee B, Wang J, Cai L, Kim M, Namburi S, Tjong H, et al. ChIA-PIPE: A fully automated pipeline for comprehensive ChIA-PET data analysis and visualization. Sci Adv. 2020;6:eaay2078.*

*2. Lareau CA, Aryee MJ. hichipper: a preprocessing pipeline for calling DNA loops from HiChIP data. Nat Methods. 2018;15:155.*

*3. Shi C, Rattray M, Orozco G. HiChIP-Peaks: A HiChIP peak calling algorithm. Bioinformatics. 2020;36:3625–31.*

*4. Li G, Fullwood MJ, Xu H, Mulawadi FH, Velkov S, Vega V, et al. ChIA-PET tool for comprehensive chromatin interaction analysis with paired-end tag sequencing. Genome Biol. 2010;11:R22.*

*5. Cao Y, Chen Z, Chen X, Ai D, Chen G, McDermott J, et al. Accurate loop calling for 3D genomic data with cLoops. Bioinformatics. 2019;*

*6. Guo Y, Krismer K, Closser M, Wichterle H, Gifford DK. High resolution discovery of chromatin interactions. Nucleic Acids Res. 2019;47:gkz051-.*

*7. Rodriguez A, Laio A. Clustering by fast search and find of density peaks. Science. 2014;344:1492–6.*

*8. Consortium TEP. An integrated encyclopedia of DNA elements in the human genome. Nature. 2012;489:57.*

*9. Fulco CP, Munschauer M, Anyoha R, Munson G, Grossman SR, Perez EM, et al. Systematic mapping of functional enhancer–promoter connections with CRISPR interference. Science. 2016;354:769–73.*

*10. Gasperini M, Hill AJ, McFaline-Figueroa JL, Martin B, Kim S, Zhang MD, et al. A Genome-wide Framework for Mapping Gene Regulation via Cellular Genetic Screens. Cell. 2019;176:377-390.e19.*

*11. Mumbach MR, Satpathy AT, Boyle EA, Dai C, Gowen BG, Cho SW, et al. Enhancer connectome in primary human cells identifies target genes of disease-associated DNA elements. Nat Genet. 2017;49:ng.3963.*

*12. Weintraub AS, Li CH, Zamudio AV, Sigova AA, Hannett NM, Day DS, et al. YY1 Is a Structural Regulator of Enhancer-Promoter Loops. Cell. 2017;171:1573-1588.e28.*

*13. Mumbach MR, Rubin AJ, Flynn RA, Dai C, Khavari PA, Greenleaf WJ, et al. HiChIP: efficient and sensitive analysis of protein-directed genome architecture. Nat Methods. 2016;13:919–22.*

*14. Hnisz D, Day DS, Young RA. Insulated Neighborhoods: Structural and Functional Units of Mammalian Gene Control. Cell. 2016;167:1188–200.*

*15. Rao SSP, Huntley MH, Durand NC, Stamenova EK, Bochkov ID, Robinson JT, et al. A 3D Map of the Human Genome at Kilobase Resolution Reveals Principles of Chromatin Looping. Cell. 2014;159:1665–80.*

*16. Huang W, Medvedovic M, Zhang J, Niu L. ChIAPoP: a new tool for ChIA-PET data analysis. Nucleic Acids Res. 2019;47:gkz062-.*

*17. Phanstiel DH, Boyle AP, Heidari N, Snyder MP. Mango: a bias-correcting ChIA-PET analysis pipeline. Bioinformatics. 2015;31:3092–8.*

**Second round of review**

**Reviewer 1**

The manuscript has been much improved and I think the authors have addressed most of my concerns. However, I have a few concerns remaining:

1. Last time, I mentioned that the lack of "true loops" makes it difficult to compare, and suggested a simulation approach. The authors have now obtained a set of "gold standard loops" from experiments where CRISPR validation was done. I appreciate the effort, but this is not a simulation approach, and as a result, there are issues. Specifically, they mention "Accuracy(ACC) was calculated for True Positive(TP), False Positive(FP), True Negative (TN) and False Negative (FN)metrics". However, I think sometimes, there may be loops that are true, but CRISPR perturbation may not lead to a change perhaps because there might be other loops around for stabilization or other reasons. Therefore, I am not sure about the "false positive" measurement. I would urge the authors to try to use some simulated data as was done with Forcato et al., on at least a portion of the genome.

2. I said last time that "The recently developed HiChIP [18]and PLAC-seq [19] protocols reveal better resolution and require lower chromatin input than ChIA-PET" is incorrect, because there is the new long-reads ChIA-PET method. The authors have cited the long-reads ChIA-PET, but I think this sentence is still present in the manuscript, and I think it is incorrect because the new long-reads ChIA-PET method has good resolution and requires less chromatin input.

3. The authors say "Unfortunately, the only publicly available long-read ChIA-PET data is from Tang et al. (2015), which doesn’t match any available HiChIP conditions. Thus, we were only able to compare long-read ChIA-PET data with standard ChIA-PET data." - I think this is OK, but it should be pointed out clearly and discussed in the manuscript that this is why there is no comparison with long read ChIA-PET data.

**Reviewer 2**

I think the work is substantially improved and addressed most of my concerns. However, I still have few concerns:

Major:

I am still confused about how their UV rate works. From the manuscript, it looks like a higher UV rate means better performance. But according to their answer to my comment 6, HiC-Pro has less UV rate since it performs "the second step removed dumped pairs, dangling ends, and self-circles, making the total number of unique valid PETs". Does this mean lower UV rate refers to more solid results? Thus, my question is why the UV rate could measure the preprocessing performance?

The authors added the overlap length as a factor in calculating PC score, which is way better than their old version. However, I am wondering what is the advantage of using max⁡(L(o_i )/L(p_i ) ,L(o_i )/L(a_i ) ) instead of using (L(o_i))/(L(a_i)) as the measure for the overlap, since the author mainly tried to measure the loop accuracy instead of ChIP-seq peak? Also, it is interesting to know the length distribution of the anchors called by different programs and also the size distribution of the public ChIP-seq data. Because in the current version of the PC equation, the anchors and ChIP-seq peak size significantly affect the results. Or the author could use L(o_i ) distribution's features, e.g. FWHM, as the PC score. Because the L(o_i )=0 already means no overlap, which includes the information of NA. Use both NA and L(o_i ) is redundant from my perspective.

Minor:

Why didn't the authors highlight the changes in the revised manuscript?

**Reviewer 3**

The manuscript has been somewhat revised in the resubmitted version with some new data added and some presentations reorganized. However, the authors did not fully address most concerns that I raised in the comments to the first submitted version, including my comment #5 completely ignored in the point-by-point response. Overall, my major concerns on the design, approach, scientific rigor, and overall quality of this work still remain. I would suggest that the authors use a recent review paper published in Genome Biology (PMID: 31221194) about essential guidelines for benchmarking work to thoroughly evaluate and rewrite the manuscript. Below is a point-by-point follow-up to the authors' response to my previous comments, with more comments to this revised version included.

Point-by-point to previous comments:

1. Fundamental flaws on the study design. The authors made some efforts by adding a "gold standard" with defined "true loops" and simulated data (see below points 1A, 1B, and 1C about issues with these new data). However, the actual ChIA-PET and HiChIP datasets (4 each) are still far from adequate as a comprehensive benchmarking study. It is not convincing to draw conclusions and recommendations based on results from these few selected datasets. The ENCODE project alone contains 81 ChIA-PET datasets from human cell lines (https://www.encodeproject.org/matrix/?type=Experiment&control_type!=*&status=released&perturbed=false) and the GEO database contains 144 data series (589 samples) for HiChIP (as of June 2021). It is essential to assess the performances of each method on a wide range of actual experimental data to ensure that any conclusion or recommendation can be generalizable and should not reflect the reference data itself. The Weber et al. 2019 paper published in Genome Biology (PMID: 31221194) suggests that all methods should be evaluated using a variety of datasets under a wide range of conditions. Several recently published benchmarking studies on Hi-C methods (e.g., PMID: 28604721, PMID: 30890172, PMID: 30526631) are good examples that the authors could follow as references.

1A. The calculation of accuracy (ACC) as described in Page 27 is fatally flawed. First, considering randomly selected genomic locations excluding promoter/enhancers as "false loops" does not make sense. It is unreasonable to assume that chromatin interaction does not exist in genomic regions other than promoter and enhancers. The actual "false loops" should be those that do not show any eQTL or CRISPR/Cas9 validated interaction and do not have any loop signal detected by Hi-C in the corresponding cell types, far more restricted than random genomic regions. Second and more importantly, the authors' definitions of false positive (FP), true negative (TN), and false negative (FN) are all wrong. FP should be the number of detected loops intersected with "false" loops; TN should be the number of "false" loops NOT intersected with detected loops; FN should be the number of gold standard loops NOT intersected with detected loops. With this error, therefore, all data shown in Fig 4 are problematic.

1B. It is unclear how the mouse ESC SMC gold standard dataset was obtained. The eQTL data and CRISPR/Cas9 validated enhancer-promoter interaction data are all from human cells.

1C. The procedure of generating simulation data lacks details. The description in the method section is very brief and inadequate for others to reproduce their work. The simulation source code should be provided to ensure reproducibility.

2. Lack of rigor in conclusions, many of which are still not supported by the data. Data presented in revised Fig. 2 are mostly reorganized from previous Figs. 2&3, but most data remain the same, with the confusion and potentially misleading problems still persist. If the motivation was to show the differences on the experimental data (ChIA-PET vs. HiChIP), a side-by-side comparison should not only have included much more data (as pointed above), but also should be performed on the same computational methods evaluated. In Fig. 2A, the results on ChIA-PET data from a group of methods and the results on HiChIP data from a different group of methods are incomparable, like comparing apples to oranges. Such a comparison does not support the conclusion that UV rate is higher for HiChIP than ChIA-PET. In Fig. 2B, the description of PCA procedure is not sufficient in the methods. Why an irrelevant tool (DiffBind) was used for PCA? Strong justification is warranted. If the authors want to provide a more systematic analysis to confirm previously known findings, more thorough and rigorous analysis should be done, including but not limited to points 3-5 below. A strong justification of motivation should also be included in the manuscript, to avoid confusion.

3, 4. Conclusions are not convincingly supported by the data about RE bias in HiChIP data. Response figure 11 is clearly a better way to present the data and to support the authors' conclusion about Mbol RE motif enrichment difference between different methods, but it is surprising that the authors did not use it in the revised main figures but still chose to use the unconvincing and potentially misleading Figure 2D in the revised manuscript.

5. The authors did not address or respond to this comment. By comparing the revised manuscript and original manuscript on this point, it looks like the authors changed the description but still failed to interpret current Figs 2F & 2G. In Fig. 2G, there are very few HiChIP peaks that are higher than ChIP-seq, indicating most HiChIP peaks should be covered by ChIP-seq, contradicting to what is described in the manuscript or shown in Fig. 2H. This is still very confusing about what these figures show and what points the authors are trying to make.

6. The authors addressed this comment, but Supplemental Fig. S4 is not mentioned or cited anywhere in the revised manuscript anymore. Does the data still need to be presented?

7. Because of the flaws in gold standard definition and ACC calculation (see above point #1), the authors' response using the flawed data shown in Response Fig. 12/Fig. 4 does not address the original comment. this concern still remains. Regardless of the ACC error, it is still unclear why cLoops performs better than CID. As CID produces 5 times as many loops as cLoops (from Fig. S8g), it is not surprising that the ACC of CID is smaller than the ACC of cLoops using current formula. Besides, the authors didn't rule out the possibility that CID is more sensitive in identifying true-weak loops, and what if the CID identified weak loops could still be true but not enriched with eQTL or CRISPR/Cas9 verified contacts. The authors should carefully draw conclusions without over-interpreting the data.

8. The "Activation Rate" and "E-P percentage" should highly depend on the total number of loops identified from each method. Without a side-by-side comparison on the total loops, the rate or percentage is not very meaningful. Fewer total loops can possibly result in higher AR or E-P percentage, but it does not necessarily mean that the "inactive" loops are false. Also, why did the author assume that AR is more important than E-P percentage and put the latter in supplemental figures? Again, this analysis should better be done using a comprehensive and correct accuracy estimation approach.

9. The practical guidance chart in Fig 6 is helpful. However, most recommendations in Fig 6 are directly based on the functions of different methods and few are based on the benchmarking results (regardless of the reliability of the results), making this final recommendation disconnected from most data presented in this manuscript. For example, HiCCUPS appears in Fig. 6 but is not mentioned nor explained throughout the manuscript. Also, as a minor point, if the color palette presents the level of performance, running time should be reversed. In the current way it is confusing that higher running time might indicate better performance.

10. In general, the authors should provide more details in the methods to ensure scientific rigor and reproducibility. Many p-values occurred without description of what statistical tests were used. If directly using other tools' output, it should be explicitly stated. The justifications described in the response to the comments should also be included in the manuscript, in which the authors did not make sufficient revisions.

Additional comments:

11. It is still confusing what "Bacon" is designed for. There is a complicated mix between the comparison of different tools (as what a benchmarking tool is supposed to include) and the comparison between ChIP-PET and HiChIP datasets. It is very confusing as the results of comparing ChIP-PET and HiChIP data (Fig. 2) are placed between the schema of Bacon (Fig. 1) and other results comparing of tools (Fig. 3-6). After all, it is unclear what the readers can use Bacon for.

12. Line 227, as another example of unsupported conclusion, that "the peak-based methods outperform the cluster-based methods at detecting loops with active enhancer signatures" is drawn from only one mES-SMC ChIA- PET/HiChIP dataset. This is unwarranted. Any conclusion generated from single or few datasets should be carefully revised or tuned down.

13. Fig. 4 is unclear on a number of aspects. What is the difference between the data columns within each dataset? They should be clearly labeled. What are the actual ACC values? Were they normalized or scaled? Also, such plots are used to present multi-dimensional data, and the size and color of the bubbles usually represent two different variables. The current way in which size and color represent the same thing is unnecessary and potentially misleading.

14. What does MICC stand for in Fig 5? What does it mean by CID+MICC? It is not described or explained anywhere in the manuscript. If the MICC tool (PMID: 26231426) was included in the manuscript, the authors should explain in details.

15. In the opening sentence in Background, the claim that "gene expression patterns are determined by genomic organization" is an overstatement. Regulation of gene expression is a complex biological process and high-dimensional genomic organization is only a component among many others. The authors should revise and tone down this statement, or precisely define what they mean by "genomic organization".

16. The authors should carefully proofread the manuscript before submitting to any journal for publication. Sloppiness in writing made the manuscript unpleasant to read. For example, in Line 36, 3C should be spelled out when appeared for the first time. In Line300, I assume "K652" should be "K562". Does "AR" mean "activation rate" (in main text) or "active rate" (in methods)? In response to Reviewer 1's Comment 6, "Principle Component" should be "Principal Component".

**Authors Response**

**Point-by-point responses to the reviewers’ comments:**

Reviewer #1:

The manuscript has been much improved, and I think the authors have addressed most of my concerns. However, I have a few concerns remaining:

1. Last time, I mentioned that the lack of "true loops" makes it difficult to compare and suggested a simulation approach. The authors have now obtained a set of "gold standard loops" from experiments where CRISPR validation was done. I appreciate the effort, but this is not a simulation approach, and as a result, there are issues. Specifically, they mention "Accuracy (ACC) was calculated for True Positive (TP), False Positive (FP), True Negative (TN) and False Negative (FN)metrics". However, I think sometimes, there may be loops that are true, but CRISPR perturbation may not lead to a change perhaps because there might be other loops around for stabilization or other reasons. Therefore, I am not sure about the "false positive" measurement. I would urge the authors to try to use some simulated data as was done with Forcato et al., on at least a portion of the genome.

*Response: Thanks for the comment. For the measurement of Accuracy (ACC), we agree that there might be some true loops neglected by CRISPR-based perturbations, thus we combined eQTL contacts and long-range interactions from ENCODE to cover as many loops as possible.*

*For the simulation approach taken by Forcato et al., a random 100 bp interval was chosen within the boundaries of a specific restriction fragment, which is not exactly applicable to ChIA-PET/HiChIP data, since these two types of data are targeted to specific protein-enriched loops. Thus, we simulated ChIA-PET data as described by Paulsen et al. [1], and simulated HiChIP data as previously described [2].*

*We have modified the Methods as follows:*

*“To test the performance of computational methods in the simulation datasets with different ChIP antibodies, we generated anchor pairs for ChIA-PET and HiChIP, according to the different data characteristics between these two types of data. For ChIA-PET anchor pairs, a Poisson model was used as described previously [1]. Firstly, the chromosomes were segmented into bins of 5000 bp, the loci of segmented points were recorded and used to construct a potential contact pairs matrix, then we simulated the interaction between genomic loci i and j as n_ij~Pois(λ_ij), the expectation λ_ij=a/(1+δ), with δ=|i-j|, a represented the number of anchors used for simulation, the interaction frequencies depend on the genomic distance strongly. The HiChIP anchor pairs were generated with Hi-C data as described by Bhattacharyya [2]. Firstly, we set the bin size (5000 bp) to extract Hi-C pairs as HiChIP anchor pairs. Secondly, the published ChIP-seq data publicly available for each antibody was used to simulate the ChIP enrichment, we then calculated ChIP-seq read coverage for the generated anchor pairs. Next, we set the coverage threshold as 50% to filter the final simulated valid pairs. Overall, we used three different ChIP antibodies to simulate the ChIA-PET and HiChIP data. Finally, we then tested the performances of different methods with these simulated datasets.”*

*Three ChIA-PET datasets and three HiChIP datasets were simulated with the ChIP antibodies of H3K27ac, YY1, and CTCF (Response table 1).*

*The ACC evaluation results of 22 experimental and 6 simulation datasets have been listed in Response Fig. 1. The AR evaluation results have been listed in Response Fig. 2 (A-B).*

*We have added Response Table 1 as Additional file 2: Table S6 and added Response Fig. 1 and Response Fig. 2 as Figure 4 and Figure 5 in the revised manuscript.*

2. I said last time that "The recently developed HiChIP [18]and PLAC-seq [19] protocols reveal better resolution and require lower chromatin input than ChIA-PET" is incorrect, because there is the new long-reads ChIA-PET method. The authors have cited the long-reads ChIA-PET, but I think this sentence is still present in the manuscript, and I think it is incorrect because the new long-reads ChIA-PET method has good resolution and requires less chromatin input.

*Response: Thanks for the pointer. We have modified the sentence as follows:*

*“The recently developed long-reads ChIA-PET [3] protocol provides better resolution and requires lower chromatin input than traditional ChIA-PET”.*

3. The authors say "Unfortunately, the only publicly available long-read ChIA-PET data is from Tang et al. (2015), which doesn’t match any available HiChIP conditions. Thus, we were only able to compare long-read ChIA-PET data with standard ChIA-PET data." - I think this is OK, but it should be pointed out clearly and discussed in the manuscript that this is why there is no comparison with long read ChIA-PET data.

*Response: Thanks for the suggestion, we have added the following to discussion:*

*“Recently, the developed long-read ChIA-PET protocols provided several advantages over traditional ChIA-PET methods ([3]). However, we were unable to fully assess analytical strategies to investigate data generated with this newer long-read approach as the only publicly available long-read ChIA-PET data is from Tang et al. (2015) [4], which doesn’t match any available HiChIP conditions (e.g. cell line and antibody). Thus, we were only able to comprehensively compare long-read ChIA-PET data with standard ChIA-PET data. Only CPT2 [5] and CPTv.3 [6] support the raw data analysis of long-read ChIA-PET data. We compared the performance of the latest approach ChIA-PIPE [7] with CPT2 and CPTv.3. The results showed that ChIA-PIPE performed slightly better than CPT2 and CPTv.3 in all the long-reads ChIA-PET datasets (Supplemental table S2). Future analytical pipelines should incorporate options for the inclusion of long-read ChIA-PET data.”*

Reviewer #2: I think the work is substantially improved and addressed most of my concerns. However, I still have few concerns:

Major:

I am still confused about how their UV rate works. From the manuscript, it looks like a higher UV rate means better performance. But according to their answer to my comment 6, HiC-Pro has less UV rate since it performs "the second step removed dumped pairs, dangling ends, and self-circles, making the total number of unique valid PETs". Does this mean lower UV rate refers to more solid results? Thus, my question is why the UV rate could measure the preprocessing performance?

*Response: In this context, a higher UV Rate means more valid PETs uniquely mapped to the reference genome, which is better for the next step in the analysis. Thus, a higher UV rate refers to more solid results, and a higher UV rate also suggests better preprocessing performance.*

*For our answer to your previous comment #6, we mentioned that “While HiC-Pro was developed to process Hi-C data, which proposed a two-step alignment, the first step removes the same parts as MAPS, and the second step removes dumped pairs, dangling ends, and self-circles, making the total number of unique valid PETs output from HiC-Pro fewer than MAPS”.*

*We apologize for the confusion, but we were attempting to explain how exactly HiC-Pro achieved a lower UV Rate compared to MAPS. Importantly, HiC-Pro was developed for Hi-C data, and was not perfectly suited for processing HiChIP data. Firstly, HiC-Pro removes all the short-range valid pairs, which MAPS includes. This is done to normalize the long-range interactions, and at least partly accounts for the elevated UV rate achieved by MAPS. Overall, UV Rate was used to evaluate how many valid PETs* (including short-range and long-range valid PETs) were detected in the preprocessing steps, and the larger the UV rate is, the more detected valid PETs there are for input into the subsequent analysis pipelines.

*We have modified the aforementioned sentence to the following in the manuscript:*

*“HiC-Pro was developed to analyze Hi-C data, and as part of its’ two-step alignment process removes dumped pairs, dangling ends, and self-circles. This effectively decreases the total number of unique valid PETs output from HiC-Pro, at least partially explaining its reduced UV rate when compared to MAPS which incorporates these categories of pairs.”*

The authors added the overlap length as a factor in calculating PC score, which is way better than their old version. However, I am wondering what is the advantage of using max⁡(L(o_i )/L(p_i ) ,L(o_i )/L(a_i ) ) instead of using (L(o_i))/(L(a_i)) as the measure for the overlap, since the author mainly tried to measure the loop accuracy instead of ChIP-seq peak? Also, it is interesting to know the length distribution of the anchors called by different programs and also the size distribution of the public ChIP-seq data. Because in the current version of the PC equation, the anchors and ChIP-seq peak size significantly affect the results. Or the author could use L(o_i ) distribution's features, e.g. FWHM, as the PC score. Because the L(o_i )=0 already means no overlap, which includes the information of NA. Use both NA and L(o_i ) is redundant from my perspective.

*Response: Thanks for the comment. As shown below, the length of anchors detected by different tools ranged from 800 bp to 6000 bp, and the peak size ranged from 200 bp to 1500 bp (Response Fig. 3). We considered that if the length of an anchor was much larger than the size of the covered peak, then the value of L(o_i)/ L(a_i) would be small. We intended to assign a high PC for large anchors. Thus, we used max{L(o_i )/L(p_i ) ,L(o_i )/L(a_i ) } instead of L(o_i)/ L(a_i) in the equation to eliminate the impact of differences between anchor length and peak size to some extent. We noticed that the results of PC didn’t show significant correlation with the length of anchors or peak size (Response Fig. 4). Therefore, the anchors and ChIP-seq peak size didn’t affect the PC results significantly.*

*We agree that FWHM is a good metric to describe the width of curve. However, the distribution of L(o_i) detected by the different tools varied considerably (Response Fig. 5), thus it is difficult to characterize the curves with a specific distribution and calculate FWHM with a fixed equation.*

*In general, the number of anchors was much larger than the number of peaks, making the overlapping set small. Hence, the results of (∑_(i=1)^N▒max⁡(L(o_i )/L(p_i ) ,L(o_i )/L(a_i ) ) )/N displayed only minimal differences between the various tools we examined. We also agree that NA and L(o_i) contain duplicated information, and the differences between PC could be expanded by duplicating this overlapping information. Through testing we found that multiplying by the factor NA in the equation can scale the PC to the range of 0-1, and could detect obvious differences between PC.*

*Response Figure 3. (A) Length of anchors detected by different tools in all testing datasets. (B) Peak size of publicly available ChIP-seq datasets.*

*Response Figure 4. (A) The Pearson’s correlation between PC and anchor length. (B) The Pearson’s correlation between PC and peak size. R, correlation coefficient. p-value calculated by t-test.*

*Response Figure 5. Distribution of the overlapping regions between anchors and ChIP-seq peaks for the different analytical tools.*

Minor:

Why didn't the authors highlight the changes in the revised manuscript?

*Response: We apologize for omitting all changes to the last revised manuscript, we have highlighted the changes in the latest revised manuscript.*

Reviewer #3:

The manuscript has been somewhat revised in the resubmitted version with some new data added and some presentations reorganized. However, the authors did not fully address most concerns that I raised in the comments to the first submitted version, including my comment #5 completely ignored in the point-by-point response. Overall, my major concerns on the design, approach, scientific rigor, and overall quality of this work still remain. I would suggest that the authors use a recent review paper published in Genome Biology (PMID: 31221194) about essential guidelines for benchmarking work to thoroughly evaluate and rewrite the manuscript. Below is a point-by-point follow-up to the authors' response to my previous comments, with more comments to this revised version included.

Point-by-point to previous comments:

1. Fundamental flaws on the study design. The authors made some efforts by adding a "gold standard" with defined "true loops" and simulated data (see below points 1A, 1B, and 1C about issues with these new data). However, the actual ChIA-PET and HiChIP datasets (4 each) are still far from adequate as a comprehensive benchmarking study. It is not convincing to draw conclusions and recommendations based on results from these few selected datasets. The ENCODE project alone contains 81 ChIA-PET datasets from human cell lines (https://www.encodeproject.org/matrix/?type=Experiment&control_type!=*&status=released&perturbed=false) and the GEO database contains 144 data series (589 samples) for HiChIP (as of June 2021). It is essential to assess the performances of each method on a wide range of actual experimental data to ensure that any conclusion or recommendation can be generalizable and should not reflect the reference data itself. The Weber et al. 2019 paper published in Genome Biology (PMID: 31221194) suggests that all methods should be evaluated using a variety of datasets under a wide range of conditions. Several recently published benchmarking studies on Hi-C methods (e.g., PMID: 28604721, PMID: 30890172, PMID: 30526631) are good examples that the authors could follow as references.

*Response: Thanks for the comment. We agree there are lots of publicly available ChIA-PET and HiChIP datasets, and a variety of datasets under a wide range of conditions should be considered. We have added more datasets to test the performance of different tools. To consider a wide range of conditions, we selected all the available antibodies for ChIA-PET/HiChIP, and chose one dataset for each antibody. When choosing the datasets, we gave priority to human cell lines, and tried to cover more platforms. Overall, we tested 10 ChIA-PET and 12 HiChIP datasets, for a total of 52 samples covering 22 types of antibodies (Response table 2 and 3).*

*We have added Response table 2 and Response table 3 to Additional file2: Table S6 in the revised version.*

1A. The calculation of accuracy (ACC) as described in Page 27 is fatally flawed. First, considering randomly selected genomic locations excluding promoter/enhancers as "false loops" does not make sense. It is unreasonable to assume that chromatin interaction does not exist in genomic regions other than promoter and enhancers. The actual "false loops" should be those that do not show any eQTL or CRISPR/Cas9 validated interaction and do not have any loop signal detected by Hi-C in the corresponding cell types, far more restricted than random genomic regions. Second and more importantly, the authors' definitions of false positive (FP), true negative (TN), and false negative (FN) are all wrong. FP should be the number of detected loops intersected with "false" loops; TN should be the number of "false" loops NOT intersected with detected loops; FN should be the number of gold standard loops NOT intersected with detected loops. With this error, therefore, all data shown in Fig 4 are problematic.

*Response: The reviewer is correct that long-range chromatin interactions do exist throughout the genome, not only at enhancers and promoters. And we agree that false loops should contain no eQTLS, CRISPR/Cas9 validated loops, or strong HiC loop signals. We have gone back and filtered out from our previous ‘false loop’ sets all that contain eQTLS, CRISPR/Cas9 validated loops, and Hi-C loops.*

*We modified the methods as follows:*

*“To evaluate the accuracy of different computational methods, we regarded gold standard loops as “true” loops, the “false” loops were constructed with the same number as “true” loops. We firstly constructed the potential false loops set by selecting the genomic location avoiding TSS regions of genes and enhancer-like regions from ENCODE, which excluded all potential enhancer-promoter loops. Then we excluded the eQTL containing loops, CRISPR/Cas9 validated loops, and strong Hi-C loop signals from the potential false loops set. Since the number of potential false loops was much greater than the “true” loops, we then randomly selected the same number of false loops from the potential loop set. To eliminate the effect of randomness, we repeated the selection three times, thus we had 3 “false loops” files for each true loops file.”*

*For the calculation of ACC, we agree that the definition given by the reviewer is correct, it is our negligence to confuse the definition of false positive (FP), true negative (TN), and false negative (FN) in the text. The ACC calculation we included in our source code (Response Fig. 6), calculated the number of true loops as sig_num, the number of false loops was equal to true loops, false_num= sig_num. The number of detected loops intersected with true loops was calculated as TP, then FN= sig_num-TP. The number of detected loops intersected with false loops was calculated as FP, then TN= false_num-FP. Thus, our definitions of TP, FP, TN and FN were the same as that mentioned by the reviewer and our results calculated by the script were correct. We corrected the definition of these metrics in the Methods as follows:*

*“Then we defined true positive (TP) as the number of detected loops which intersected with gold standard loops, false positive (FP) as the number of detected loops that intersected with "false" loops, true negative (TN) as the number of "false" loops NOT intersected with detected loops, and false negative (FN) as the number of gold standard loops NOT intersected with detected loops. Since there were three “false” loop files for each true loop file, we calculated the mean of three FP as the final FP value to calculate accuracy. The accuracy (ACC) was calculated by following,*

*ACC=(TP+TN)/(TP+TN+FP+FN)”*

Response Figure 6. The calculation of TP, FP, FN, TN in Bacon source code.

1B. It is unclear how the mouse ESC SMC gold standard dataset was obtained. The eQTL data and CRISPR/Cas9 validated enhancer-promoter interaction data are all from human cells.

*Response: Thanks for the pointer. For the datasets which lacked publicly available eQTL or CRISPR/Cas9 validated datasets, such as mESC-Smc1. Firstly, we downloaded the long-range interactions from the GEO database (under accession number of GSE57911) as candidate loops, we then used mESC Smc1 ChIP-seq peaks (GSE22562) to count the coverage for both anchors in a similar fashion to what was done with the human datasets. The final murine ‘gold standard’ loop set for mESC-Smc1 was constructed by selecting the candidate loops significantly overlapping mESC Smc1 ChIP-seq peaks (p-value<0.05 and FDR<0.05).*

*At the suggestion of the reviewer, we have added the following to the Methods section:*

*“For mESC-Smc1, mESC-Yy1, mESC-Fgfr1, erythroblast-Gata1, mESC-Klf4, BMDM-H3K4me3, mESC-H3K27me3, and AML12-H3K9me3, no publicly available eQTLs or CRISPR/Cas9 validated data existed for the calling of gold standard loops. To generate gold standard murine loops, we used the ChIP-seq peaks of corresponding cell line and antibody (GSE22562 [9], GSE31785 [10], GSE65698 [11], GSE112717 [12], GSE113429 [13], GSE23619 [14], GSE162739 [15], GSE143886[16]) to count the coverage for both anchors, then calculated the significance.”*

1C. The procedure of generating simulation data lacks details. The description in the method section is very brief and inadequate for others to reproduce their work. The simulation source code should be provided to ensure reproducibility.

*Response: Thanks for the suggestion. We have added a detailed description of the simulation procedure to the Methods. Also, please see comment 1 from reviewer #1 (above).*

*The source code for performing the simulation has also been uploaded to the GitHub page: https://github.com/CSUBioGroup/Bacon*

2. Lack of rigor in conclusions, many of which are still not supported by the data. Data presented in revised Fig. 2 are mostly reorganized from previous Figs. 2&3, but most data remain the same, with the confusion and potentially misleading problems still persist. If the motivation was to show the differences on the experimental data (ChIA-PET vs. HiChIP), a side-by-side comparison should not only have included much more data (as pointed above), but also should be performed on the same computational methods evaluated. In Fig. 2A, the results on ChIA-PET data from a group of methods and the results on HiChIP data from a different group of methods are incomparable, like comparing apples to oranges. Such a comparison does not support the conclusion that UV rate is higher for HiChIP than ChIA-PET. In Fig. 2B, the description of PCA procedure is not sufficient in the methods. Why an irrelevant tool (DiffBind) was used for PCA? Strong justification is warranted. If the authors want to provide a more systematic analysis to confirm previously known findings, more thorough and rigorous analysis should be done, including but not limited to points 3-5 below. A strong justification of motivation should also be included in the manuscript, to avoid confusion.

*Response: To increase the clarity of Figure 2, we have split up the UV rate comparisons of HiChIP and ChIA-PET, and we have removed the motif enrichment plot for MboI (Response Fig. 7). We have also toned down our comparison of the two methods in the results.*

*DiffBind is an R package for differential binding analysis, often used for analyzing ChIP-seq and even ATAC-seq datasets. Given that ChIP-seq, HiChIP, and ChIA-PET all rely on chromatin immunoprecipitation, they should display similar binding profiles when comparisons are being made for a single protein of interest. We implemented DiffBind to perform PCA analysis and to give a deeper insight into how these experimental groups were associated. For this we focused only on Smc1 datasets. Thus, we are using the DiffBind tool to investigate differentially bound, or captured, Smc1 peaks, which is the* type *of analysis this package was designed to perform. To make this clearer we have updated the figure labels as well as the results section (Response Fig. 7A).*

*We have added more details to Methods:*

*“Given that ChIP-seq, HiChIP, and ChIA-PET all rely on chromatin immunoprecipitation, they should display similar binding profiles when comparisons are being made for a single protein of interest. We implemented DiffBind to perform PCA analysis and to give a deeper insight into how these experimental groups were associated. For this we focused only on mESC Smc1 datasets. The alignment files derived from ChIP-seq, ChIA-PET, and HiChIP replicates were filtered using Samtools with MAPQ 30. Picard was then used to remove duplicates, and the filtered bam files were prepared as the input for DiffBind. The “plotPCA” function was used to implement the analysis and generate PCA plots.”*

*We have modified Fig. 1 as follows in the revised manuscript:*

*Response Figure 7. Comparing data characteristics of ChIA-PET and HiChIP. (A) PCA plot for replicates of mESC-Smc1 ChIP-seq, ChIA-PET, HiChIP reads (B) Genome browser tracks of Smc1 ChIP-seq, ChIA-PET and HiChIP in mESC. The green bars of upper plot indicate the HiChIP reads which overlap with Mbol restriction sites, when no ChIP-seq/ChIA-PET reads are enriched. Green bars on the lower track indicate HiChIP reads that are sparse in positions without Mbol restriction sites, when there are ChIP-seq/ChIA-PET reads enriched. (C) The counts distribution of Mbol restriction enzyme sites across different peak types. (D) The overlap between HiChIP peaks and Mbol restriction sites. (E) The overlap between ChIP-seq peaks and Mbol restriction sites. (F) The overlap between ChIA-PET peaks and Mbol restriction sites. (G, H) Differential volcano plots comparing ChIP-seq with ChIA-PET and HiChIP peaks quantitatively. (I) Peak co-occupancy between ChIP-seq/ChIA-PET, and ChIP-seq/HiChIP. Bar plot drawn with standard error bar. ****, p-value < 1e-4, p-value was calculated by t-test.*

3, 4. Conclusions are not convincingly supported by the data about RE bias in HiChIP data. Response figure 11 is clearly a better way to present the data and to support the authors' conclusion about Mbol RE motif enrichment difference between different methods, but it is surprising that the authors did not use it in the revised main figures but still chose to use the unconvincing and potentially misleading Figure 2D in the revised manuscript.

*Response: Thanks for the suggestion. We have replaced the figure. See Response Fig. 7C (above).*

5. The authors did not address or respond to this comment. By comparing the revised manuscript and original manuscript on this point, it looks like the authors changed the description but still failed to interpret current Figs 2F & 2G. In Fig. 2G, there are very few HiChIP peaks that are higher than ChIP-seq, indicating most HiChIP peaks should be covered by ChIP-seq, contradicting to what is described in the manuscript or shown in Fig. 2H. This is still very confusing about what these figures show and what points the authors are trying to make.

*Response: We apologize for the confusion associated with the figures. With this analysis we are trying to show how the peaks output from the two methods compare to ChIP-seq derived peak calls all performed with the same antibody, and in the same cell line. An important control for evaluating HiChIP and ChIA-PET experiments is a favorable overlap with ChIP-seq data. So, we performed differential analysis for ChIA-PET/ChIP-seq peaks and HiChIP/ChIP-seq peaks. The results showed that there were 4,677 differential ChIA-PET peaks in Fig. 2F (Response Fig. 7G), and there were 23,873 differential HiChIP peaks in Fig. 2G (Response Fig. 7H). Both HiChIP and ChIA-PET output more peaks than ChIP-seq alone. To further investigate the differences between ChIA-PET, HiChIP and ChIP-seq peaks, we overlapped the ChIA-PET/HiChIP peaks with ChIP-seq peaks, the results showed over 40% ChIA-PET peaks overlapped with ChIP-seq, while only 10% HiChIP peaks overlapped with ChIP-seq (Response Fig. 7I). These results highlight the distinctions between the two methods when compared to ChIP-seq, and show that both will produce more peaks than expected when looking at ChIP-seq alone. Further, these highlights the fact that HiChIP produces much more differential peaks than ChIA-PET, and is overall not in excellent agreement with ChIP-seq peaks.*

*We have modified the figure labels to aid in interpretation and clear up any confusion. See Response Fig. 7G-7H (above). Also, we have updated the text in the Results to read as follows:*

*“An important control for evaluating HiChIP and ChIA-PET experiments is a favorable overlap with ChIP-seq data. We next performed differential analysis between ChIA-PET, ChIP-seq, and HiChIP datasets. A total of 4,677 peaks that differed significantly between ChIA-PET and ChIP-seq were identified, and 3,290 of these were ChIP-seq enriched (Fig. 1G). Conversely, 23,873 peaks differed between ChIP-seq and HiChIP datasets, the 21,485 of which were HiChIP-specific (Fig. 1H). We then looked at the peaks each dataset had in common with ChIP-seq, and found that 41.3% of ChIA-PET peaks overlapped with Smc1 ChIP-seq peaks compared to 10% overlap of ChIP-seq with HiChIP peaks (Fig. 1I).”*

6. The authors addressed this comment, but Supplemental Fig. S4 is not mentioned or cited anywhere in the revised manuscript anymore. Does the data still need to be presented?

*Response: Thanks for the pointer. We have added the citation in the revised manuscript as follows:*

*“A HiChIP-specific software package known as Hichipper supports three types of inputs: reference peaks, only self- and dangling peaks, and all HiChIP peaks (Additional file 1: Fig. S4).”*

7. Because of the flaws in gold standard definition and ACC calculation (see above point #1), the authors' response using the flawed data shown in Response Fig. 12/Fig. 4 does not address the original comment. this concern still remains. Regardless of the ACC error, it is still unclear why cLoops performs better than CID. As CID produces 5 times as many loops as cLoops (from Fig. S8g), it is not surprising that the ACC of CID is smaller than the ACC of cLoops using current formula. Besides, the authors didn't rule out the possibility that CID is more sensitive in identifying true-weak loops, and what if the CID identified weak loops could still be true but not enriched with eQTL or CRISPR/Cas9 verified contacts. The authors should carefully draw conclusions without over-interpreting the data.

*Response: Thanks for the comment. We have explained the problem about ACC in comment 1A. We agree that CID could be more sensitive in identifying true-weak loops, which might not be covered by eQTL or CRISPR/Cas9 verified contacts. We have modified the results to state the following:*

*“Overall, we found that cLoops achieved a higher global ES than CID for the datasets we evaluated, indicating cLoops can detect more highly enriched (strong) loops, however, there still exists the possibility that CID is more sensitive in identifying true-weak loops.”*

8. The "Activation Rate" and "E-P percentage" should highly depend on the total number of loops identified from each method. Without a side-by-side comparison on the total loops, the rate or percentage is not very meaningful. Fewer total loops can possibly result in higher AR or E-P percentage, but it does not necessarily mean that the "inactive" loops are false. Also, why did the author assume that AR is more important than E-P percentage and put the latter in supplemental figures? Again, this analysis should better be done using a comprehensive and correct accuracy estimation approach.

*Response: Thanks for the thoughtful comment. To investigate whether AR/E-P percentage depended on the total number of loops, we performed Pearson’s correlation analysis, and the results showed that there was no significant correlation between AR and the number of loops (Response Fig. 8A). Also, we did not uncover a significant correlation between EP-percentage and the total number of loops (Response Figure 8B). We agree that the “inactive” loops may also be meaningful, true, and biologically relevant.*

*Response Figure 8. (A) The Pearson’s correlation between the number of loop and Activation Rate (AR). (B) The Pearson’s correlation between the number of loops and E-P percentage. R, correlation coefficient. p-value calculated by t-test.*

*As we described in the manuscript “we utilized candidate enhancer-like and promoter-like signatures from ENCODE [17] to annotate loops. Next, H3K27ac, H3K4me1, H3K4me3, and H3K27me3 ChIP-seq datasets were used to calculate the Activation Rate (AR) of enhancer-mediated loops”. The results of AR were calculated based on the annotation of Enhancer/Promoter, and we believe that AR is a more accurate metric as it incorporates several layers of epigenetic information for assessing active distal regulatory interactions with target genes, which is often a primary goal for performing ChIA-PET and HiChIP.*

9. The practical guidance chart in Fig 6 is helpful. However, most recommendations in Fig 6 are directly based on the functions of different methods and few are based on the benchmarking results (regardless of the reliability of the results), making this final recommendation disconnected from most data presented in this manuscript. For example, HiCCUPS appears in Fig. 6 but is not mentioned nor explained throughout the manuscript. Also, as a minor point, if the color palette presents the level of performance, running time should be reversed. In the current way it is confusing that higher running time might indicate better performance.

*Response: Thanks for the feedback. In Fig.6, except for the function recommendations of different tools, we added the annotations based on benchmarking results (such as UV Rate, PC/ES, ACC, AR, and running time). Through this guidance, users can choose suitable tools for different conditions as well as have an idea about the performance of different tools (Response Fig. 9).*

*We have replaced Fig. 6 with Response Fig. 9 in the revised manuscript.*

*HiCCUPS is a loop caller for Hi-C data, which also can be used to call HiChIP loops [20]. Thus, we only mentioned it this later stage in the manuscript where we assessed loop calling. At the suggestion of the reviewer, we added the following to introduction:*

*“Also, HiCCUPS is a loop caller for Hi-C data, which also can be used to call HiChIP loops [20].”*

*We have adjusted the color palette for running time as per the reviewer’s request (see above, Response Fig. 9).*

10. In general, the authors should provide more details in the methods to ensure scientific rigor and reproducibility. Many p-values occurred without description of what statistical tests were used. If directly using other tools' output, it should be explicitly stated. The justifications described in the response to the comments should also be included in the manuscript, in which the authors did not make sufficient revisions.

*Response: Thanks for the suggestions, we have added more details for calculation of Uniquely mapped Valid Rate (UV Rate), gathering gold standard loop sets, calculation of accuracy, simulation of ChIA-PET and HiChIP data, and PCA analysis for the replicates of ChIP-seq, ChIA-PET and HiChIP in the Methods.*

*And added the statistics test of p-value to the figure legend as follows,*

*In the revised manuscript,*

*Figure 1. ****, p-value < 1e-4, p-value was calculated by t-test.*

*Figure 3. *** p-value<1e-3, p-value was calculated by t-test.*

*In the Additional file1,*

*Figure S4. ***, p-value <1e-3, p-value was calculated by t-test.*

*Figure S10. *** p-value<1e-3, p-value was calculated by t-test.*

Additional comments:

11. It is still confusing what "Bacon" is designed for. There is a complicated mix between the comparison of different tools (as what a benchmarking tool is supposed to include) and the comparison between ChIP-PET and HiChIP datasets. It is very confusing as the results of comparing ChIP-PET and HiChIP data (Fig. 2) are placed between the schema of Bacon (Fig. 1) and other results comparing of tools (Fig. 3-6). After all, it is unclear what the readers can use Bacon for.

*Response: We apologize for the confusion. We thank the reviewer for the helpful suggestion for rearranging the figures to improve clarity regarding the layout and design of Bacon. We have moved the previous figure 2, to figure 1, and vice versa. Thus, we firstly explore the differences and biases that exist for HiChIP, and ChIA-PET upfront and then move into the schema of Bacon once these points are established. We believe this does help improve the flow of the manuscript, and separate the comparison of the two closely related protocols with the benchmarking functionality of Bacon.*

12. Line 227, as another example of unsupported conclusion, that "the peak-based methods outperform the cluster-based methods at detecting loops with active enhancer signatures" is drawn from only one mES-SMC ChIA- PET/HiChIP dataset. This is unwarranted. Any conclusion generated from single or few datasets should be carefully revised or tuned down.

*Response: We have tuned down the conclusion as follows:*

*“To further investigate the properties of the different loops called by these methods, we chose ChIAPoP as the representative ChIA-PET peak-based method, and compared the ChIAPoP-specific loops, cLoops-specific loops, and CID-specific loops with active and inactive histone mark ChIP-seq data. The results showed that ChIAPoP produces more active chromatin-enriched peaks with higher H3K27ac signal than cLoops and CID, while cLoops and CID output loops with more inactive H3K27me3-enriched profiles (Additional file 1: Fig. S9A) in the dataset we analyzed. For the analysis of HiChIP, we chose Hichipper(+chip) as representative, and the comparisons showed similar results to what was observed for ChIA-PET (Additional file 1: Fig. S9B).”*

13. Fig. 4 is unclear on a number of aspects. What is the difference between the data columns within each dataset? They should be clearly labeled. What are the actual ACC values? Were they normalized or scaled? Also, such plots are used to present multi-dimensional data, and the size and color of the bubbles usually represent two different variables. The current way in which size and color represent the same thing is unnecessary and potentially misleading.

*Response: In the revised manuscript, we have added more datasets, limited by the space, we calculated the mean value of three ACC for each dataset, and re-scaled the mean ACC value (from 0-1), which is now shown in Response Fig. 1. The actual ACC values are listed in Additional file 3: Table S7 and Additional file 4: Table S8. At the suggestion of reviewer, we have modified the label of ACC, and only used color to represent the value of scaled ACC in Fig. 4.*

14. What does MICC stand for in Fig 5? What does it mean by CID+MICC? It is not described or explained anywhere in the manuscript. If the MICC tool (PMID: 26231426) was included in the manuscript, the authors should explain in details.

*Response: The Model based Interaction Calling from ChIA-PET (MICC) tool (PMID: 26231426) is a significance detection method, since CID can only call loops, as suggested by Guo et al. [21], we utilized MICC to calculate the significance for CID loops. CID+MICC means using MICC to detect the significance of the loops output from CID.*

*At the suggestion of reviewer, we have added the following to the introduction:*

*“The Model based Interaction Calling from ChIA-PET (MICC) uses a Bayesian mixture model to systematically remove random ligation and random collision noise [22].”*

*And added the following to results:*

*“Since CID can only call loops, as suggested by Guo et al. [21], we utilized the MICC algorithm [22] to identify significance for CID loops.”*

*Further, we added the following to the legend of Fig.5:*

*“CID+MICC, indicates the combination of CID loop calling and significance detection with the MICC package”*

15. In the opening sentence in Background, the claim that "gene expression patterns are determined by genomic organization" is an overstatement. Regulation of gene expression is a complex biological process and high-dimensional genomic organization is only a component among many others. The authors should revise and tone down this statement, or precisely define what they mean by "genomic organization".

*Response: Sorry for the confusion, we completely agree. We have amended the first sentence to read as follows:*

*“There is sufficient evidence genomic organization, whereby protein complexes contribute to the formation of long-range physical contacts between distal regulatory elements, plays an important role in dictating gene expression patterns.”*

16. The authors should carefully proofread the manuscript before submitting to any journal for publication. Sloppiness in writing made the manuscript unpleasant to read. For example, in Line 36, 3C should be spelled out when appeared for the first time. In Line300, I assume "K652" should be "K562". Does "AR" mean "activation rate" (in main text) or "active rate" (in methods)? In response to Reviewer 1's Comment 6, "Principle Component" should be "Principal Component".

*Response: Thanks for the suggestion, we have checked the manuscript carefully and modified all the mistakes.*

*References*

*1. Paulsen J, Rødland EA, Holden L, Holden M, Hovig E. A statistical model of ChIA-PET data for accurate detection of chromatin 3D interactions. Nucleic Acids Res. 2014;42:e143–e143.*

*2. Bhattacharyya S, Chandra V, Vijayanand P, Ay F. Identification of significant chromatin contacts from HiChIP data by FitHiChIP. Nat Commun. 2019;10:4221.*

*3. Li X, Luo OJ, Wang P, Zheng M, Wang D, Piecuch E, et al. Long-read ChIA-PET for base-pair-resolution mapping of haplotype-specific chromatin interactions. Nat Protoc. 2017;12:899–915.*

*4. Tang Z, Luo OJ, Li X, Zheng M, Zhu JJ, Szalaj P, et al. CTCF-Mediated Human 3D Genome Architecture Reveals Chromatin Topology for Transcription. Cell. 2015;163:1611–27.*

*5. Li G, Chen Y, Snyder MP, Zhang MQ. ChIA-PET2: a versatile and flexible pipeline for ChIA-PET data analysis. Nucleic Acids Res. 2017;45:e4–e4.*

*6. Li G, Sun T, Chang H, Cai L, Hong P, Zhou Q. Chromatin Interaction Analysis with Updated ChIA-PET Tool (V3). Genes-basel. 2019;10:554.*

*7. Lee B, Wang J, Cai L, Kim M, Namburi S, Tjong H, et al. ChIA-PIPE: A fully automated pipeline for comprehensive ChIA-PET data analysis and visualization. Sci Adv. 2020;6:eaay2078.*

*8. Greenwald WW, Li H, Benaglio P, Jakubosky D, Matsui H, Schmitt A, et al. Subtle changes in chromatin loop contact propensity are associated with differential gene regulation and expression. Nat Commun. 2019;10:1054.*

*9. Kagey MH, Newman JJ, Bilodeau S, Zhan Y, Orlando DA, Berkum NL van, et al. Mediator and cohesin connect gene expression and chromatin architecture. Nature. 2010;467:430–5.*

*10. Vella P, Barozzi I, Cuomo A, Bonaldi T, Pasini D. Yin Yang 1 extends the Myc-related transcription factors network in embryonic stem cells. Nucleic Acids Res. 2012;40:3403–18.*

*11. Terranova C, Narla ST, Lee Y-W, Bard J, Parikh A, Stachowiak EK, et al. Global Developmental Gene Programing Involves a Nuclear Form of Fibroblast Growth Factor Receptor-1 (FGFR1). Plos One. 2015;10:e0123380.*

*12. Cai W, Huang J, Zhu Q, Li BE, Seruggia D, Zhou P, et al. Enhancer dependence of cell-type–specific gene expression increases with developmental age. Proc National Acad Sci. 2020;117:21450–8.*

*13. Giammartino DCD, Kloetgen A, Polyzos A, Liu Y, Kim D, Murphy D, et al. KLF4 binding is involved in the organization and regulation of 3D enhancer networks during acquisition and maintenance of pluripotency. Biorxiv. 2019;382473.*

*14. Escoubet-Lozach L, Benner C, Kaikkonen MU, Lozach J, Heinz S, Spann NJ, et al. Mechanisms Establishing TLR4-Responsive Activation States of Inflammatory Response Genes. Plos Genet. 2011;7:e1002401.*

*15. Conway E, Rossi F, Tamburri S, Ponzo E, Ferrari KJ, Zanotti M, et al. BAP1 activity regulates PcG occupancy and global chromatin condensation counteracting diffuse PCGF3/5-dependent H2AK119ub1 deposition. Biorxiv. 2020;2020.12.10.419309.*

*16. Ji L, Huo X, Zhang Y, Yan Z, Wang Q, Wen B. TOPORS, a tumor suppressor protein, contributes to the maintenance of higher-order chromatin architecture. Biochimica Et Biophysica Acta Bba - Gene Regul Mech. 2020;1863:194518.*

*17. Consortium TEP. An integrated encyclopedia of DNA elements in the human genome. Nature. 2012;489:57.*

*18. Gasperini M, Hill AJ, McFaline-Figueroa JL, Martin B, Kim S, Zhang MD, et al. A Genome-wide Framework for Mapping Gene Regulation via Cellular Genetic Screens. Cell. 2019;176:377-390.e19.*

*19. Fulco CP, Munschauer M, Anyoha R, Munson G, Grossman SR, Perez EM, et al. Systematic mapping of functional enhancer–promoter connections with CRISPR interference. Science. 2016;354:769–73.*

*20. Durand NC, Shamim MS, Machol I, Rao SSP, Huntley MH, Lander ES, et al. Juicer Provides a One-Click System for Analyzing Loop-Resolution Hi-C Experiments. Cell Syst. 2016;3:95–8.*

*21. Guo Y, Krismer K, Closser M, Wichterle H, Gifford DK. High resolution discovery of chromatin interactions. Nucleic Acids Res. 2019;47:gkz051-.*

*22. He C, Zhang MQ, Wang X. MICC: an R package for identifying chromatin interactions from ChIA-PET data. Bioinformatics. 2015;31:3832–4.*

**Third round of review**

**Reviewer 1**

My concerns have been addressed.

**Reviewer 2**

The authors have now addressed my concerns.

**Reviewer 3**

The revised manuscript has been improved and some of the comments to the previous version have been addressed. However, major concerns still remain, reflecting issues of scientific rigor.

1. It is an improvement to expand the benchmarking from 4 experimental datasets to 22 datasets. However, the data characteristics of ChIA-PET and HiChIP described in the first subsection of Results were still based on the initial 2 Smc1 datasets. As repeated stated in the previous comments, characteristics of two data types should be assessed by analyzing a significant number of existing datasets, but not from only 2 datasets. None of the observations made from comparing only 2 datasets, shown in Fig. 1, can be used to support such big conclusions as “data characteristics” of ChIA-PET and HiChIP. In order to make convincing data characterization, the authors should have included more datasets for both ChIA-PET and HiChIP, as they already collected more datasets. For example, K562 H3K27ac has both data types available. Various sequencing depths should not have been an excuse, but an opportunity to characterize as an important factor. It is a little surprising and disappointing that the authors missed the opportunity to take advantage of more data they already collected to generate a comprehensive and thorough analysis to support the results.

2. I understand that diffBind can be used for differential binding analysis. But Fig. 1A is a PCA from all reads from different data types, not a differential binding analysis. It is still unclear how this analysis was done and why PCA was performed using such a specific tool instead of many commonly used PCA packages in R. No detailed description in the methods or in the code repository make it difficult to interpret or evaluate.

3. The authors claimed that the selection of “false loops” have been corrected. But the method description is still not sufficient for reproducibility. How are “strong Hi-C loop signals” defined and quantified? What are the thresholds and justification? Also, the authors should provide source code and complete scripts that were used to generate the gold stand loops in this study, to ensure reproducibility. Currently there are only result files (gold standard loops) but no code or script is available to show how these data files were generated. As the accuracy analysis is the most important part of this work, the lack of rigor and details make it difficult to evaluate the results.

4. As a benchmarking study, the authors should provide all the code and scripts that they used to generate all data and results, including complete command lines with all parameter settings used for running the benchmarked tools. Currently they are completely missing on the website or in the GitHub repository. Without this, Bacon is useless, and no result from this work can be considered reproducible.

**Authors Response**

**Point-by-point responses to the reviewers’ comments:**

Reviewer #3:

The revised manuscript has been improved and some of the comments to the previous version have been addressed. However, major concerns still remain, reflecting issues of scientific rigor.

1. It is an improvement to expand the benchmarking from 4 experimental datasets to 22 datasets. However, the data characteristics of ChIA-PET and HiChIP described in the first subsection of Results were still based on the initial 2 Smc1 datasets. As repeated stated in the previous comments, characteristics of two data types should be assessed by analyzing a significant number of existing datasets, but not from only 2 datasets. None of the observations made from comparing only 2 datasets, shown in Fig. 1, can be used to support such big conclusions as “data characteristics” of ChIA-PET and HiChIP. In order to make convincing data characterization, the authors should have included more datasets for both ChIA-PET and HiChIP, as they already collected more datasets. For example, K562 H3K27ac has both data types available. Various sequencing depths should not have been an excuse, but an opportunity to characterize as an important factor. It is a little surprising and disappointing that the authors missed the opportunity to take advantage of more data they already collected to generate a comprehensive and thorough analysis to support the results.

***Response****: Thanks for the suggestions. We have added a total of 12 HiChIP, 10 ChIA-PET and 20 ChIP-seq datasets in the same manner as we did for mESC-Smc1. The results from this latest analysis are consistent with our SMC1 analysis (****Response Fig.1****).*

*We have added* ***Response Fig.1*** *to Supplementary Material as* ***Fig. S1.***

*We also modified the “Results” in the revised manuscript as follows:*

*“****Data characteristics of ChIA-PET and HiChIP***

*HiChIP and ChIA-PET both produce similar information about protein-specific topological interactions and are interpreted in much the same way. However, the two protocols differ greatly in how they capture this information. One important distinction between the two, is that HiChIP incorporates restriction endonucleases to fragment the genome, while ChIA-PET traditionally relies upon sonication. And while this difference has been taken into account by some computational pipelines [31], a full characterization of the differences and similarities that exist between HiChIP and ChIA-PET is lacking. We focused on the mESC-SMC1a (Smc1) and K562-H3K27ac datasets for the analysis, which were performed in the same cell type, and with the same antibody for the HiChIP and ChIA-PET experiments. To characterize the read properties of HiChIP and ChIA-PET libraries, we compared their alignments with publicly available ChIP-seq data (for the processing of ChIP-seq data, see methods). Principal Component Analysis (PCA) showed that the ChIP-seq, ChIA-PET, and HiChIP replicates clustered together into their respective experimental groups (****Fig. 1A****) (for PCA analysis, see methods). Notably, HiChIP data was heavily impacted by restriction enzyme treatment, as the read distribution of HiChIP presented restriction enzyme cut site bias. Correspondingly, HiChIP reads were sparse in positions without Mbol restriction sites (****Fig. 1B****). To compare the bias of these two methodologies in a quantitative manner, we called peaks for Smc1 ChIP-seq, ChIA-PET and HiChIP datasets separately, and then calculated the distribution of Mbol restriction enzyme motifs near peaks. We found the counts distribution of restriction enzyme sites also showed more enzyme sites located within HiChIP peaks (****Fig. 1C*** *and Additional file 1:* ***Fig. S1A****). Overall, 62.19% of HiChIP peaks overlapped with Mbol restriction enzyme sites (****Fig. 1D-1F****).*

*An important control for evaluating HiChIP and ChIA-PET experiments is a favorable overlap with ChIP-seq data. We next performed differential analysis between ChIA-PET, HiChIP, and ChIP-seq datasets. A total of 4,677 peaks that differed significantly between ChIA-PET and ChIP-seq were identified, and 3,290 of these were ChIP-seq enriched (****Fig. 1G****). Conversely, 23,873 peaks differed between ChIP-seq and HiChIP datasets, the 21,485 of which were HiChIP-specific (****Fig. 1H****). We then looked at the peaks in each dataset had in common with that of ChIP-seq, and found that 41.3% of ChIA-PET peaks overlapped with Smc1 ChIP-seq peaks compared to 10% overlap of ChIP-seq with HiChIP peaks (****Fig. 1I*** *and Additional file 1:* ***Fig. S1B****). Hence, HiChIP data generates more loops with elevated sensitivity compared to ChIA-PET, however, HiChIP produces data with a strong restriction enzyme site bias and low overall agreement with ChIP-seq data.”*

2. I understand that diffBind can be used for differential binding analysis. But Fig. 1A is a PCA from all reads from different data types, not a differential binding analysis. It is still unclear how this analysis was done and why PCA was performed using such a specific tool instead of many commonly used PCA packages in R. No detailed description in the methods or in the code repository make it difficult to interpret or evaluate.

***Response****: Thanks for the comment. We agree that there are many PCA packages in R. To try and compare them with our results we used the pcaExplorer package to analyze all the same samples. We found that the results obtained with pcaExplorer were consistent with the results obtained with DiffBind (****Response Fig. 2****).*

***Response Figure 2. PCA analysis with pcaExplorer.***

*In DiffBind, filtered alignment files and corresponding peak sets were used as input to function “dba”, then a binding matrix with scores was calculated based on read counts for every sample with function “dba.count”. The data were normalized based on sequencing depth with the default settings for the function “dba.normalize”, then the function “dba.plotPCA” was used to see how well the samples cluster with one another (Fig 1. A).*

*Before running the differential analysis, we used the “dba.contrast” function with default settings to model the data, as well as specify the comparisons we were interested (e.g. ChIP-seq vs ChIA-PET and ChIP-seq vs HiChIP). Next, the “dba.analyze” function was used to perform the differential analysis, and the p-value < 0.05 and log2(fold change) > 1 was used as the threshold of significance to detect differential peaks. The significant differential peaks were indicated by red in the volcano plot (Fig 1. G, H).*

*To make it easier to interpret, we have combined the PCA analysis and differential analysis in the Methods. We also modified the “Methods” in the revised manuscript as follows:*

*“****PCA and differential analysis for ChIP-seq, ChIA-PET and HiChIP***

*Given that ChIP-seq, HiChIP, and ChIA-PET all rely on chromatin immunoprecipitation, they should display similar binding profiles when comparisons are being made for a single protein of interest. We implemented DiffBind to perform PCA analysis and to give a deeper insight into how these experimental groups were associated. The alignment files derived from ChIP-seq, ChIA-PET, and HiChIP replicates were filtered using Samtools [68] with minimum MAPQ 30. Picard was then used to remove duplicates, and the filtered bam files were prepared as the input to function “dba”, then a binding matrix with scores was calculated based on read counts for every sample with function “dba.count”. The data were normalized based on sequencing depth with default setting of function “dba.normalize”, then the function “dba.plotPCA” was used to see how well the samples cluster with one another.*

*Before running the differential analysis, we used “dba.contrast” function with default mode to model the data, as well as specify the comparisons we interested in are ChIP-seq vs ChIA-PET and ChIP-seq vs HiChIP. Then “dba.analyze” function was used to perform the differential analysis, and the p-value <0.05 and log2(fold change)>1 was used as the threshold of significance to detect differential peaks.”*

3. The authors claimed that the selection of “false loops” have been corrected. But the method description is still not sufficient for reproducibility. How are “strong Hi-C loop signals” defined and quantified? What are the thresholds and justification? Also, the authors should provide source code and complete scripts that were used to generate the gold stand loops in this study, to ensure reproducibility. Currently there are only result files (gold standard loops) but no code or script is available to show how these data files were generated. As the accuracy analysis is the most important part of this work, the lack of rigor and details make it difficult to evaluate the results.

***Response****: Thanks for the comment. To make it clear, we added the generation of Hi-C loops with strong signals to the “Methods”:*

*“****Generation of Hi-C loops with strong signals***

*For the cell lines of K562, GM12878, mESC, MCF7, HeLa-S3, and AML12, the compressed binary (.hic) files were downloaded from* [*https://bcm.app.box.com/v/aidenlab/folder/11234760671*](https://bcm.app.box.com/v/aidenlab/folder/11234760671)*, and GSE134621. Then HiCCUPS was used to call loops for .hic files with the resolution of 1Mb, and KR (Knight-Ruiz) balancing mode was used to normalize the data, the loops with strong signals were defined as FDR <=0.01 and observed counts >=5. For BMDMs, erythroblast and PAEC, the Hi-C interactions were downloaded from GEO under accession number of GSE109965, GSE168176 and GSE152900, then we filtered the interactions with a threshold of 5.”*

*We’ve also uploaded the script for generating gold standard loops and false loops to the GitHub repository (*[*https://github.com/CSUBioGroup/Bacon*](https://github.com/CSUBioGroup/Bacon)*) and Zenodo (10.5281/zenodo.5607035).*

4. As a benchmarking study, the authors should provide all the code and scripts that they used to generate all data and results, including complete command lines with all parameter settings used for running the benchmarked tools. Currently they are completely missing on the website or in the GitHub repository. Without this, Bacon is useless, and no result from this work can be considered reproducible.

***Response****: Thanks for the comment. We have showed all the parameter settings and command lines used for running the benchmarked tools on the web page of* [*https://csuligroup.com/Bacon/ToolsChIAPET*](https://csuligroup.com/Bacon/ToolsChIAPET) *and* [*https://csuligroup.com/Bacon/ToolsHiChIP*](https://csuligroup.com/Bacon/ToolsHiChIP)*, the complete bash scripts were also uploaded to the GitHub repository (*[*https://github.com/CSUBioGroup/Bacon*](https://github.com/CSUBioGroup/Bacon)*) and Zenodo (10.5281/zenodo.5607035).*

**Fourth round of review**

**Reviewer 1**

I am re-reviewing this manuscript in order to assess whether the other reviewers' comments have been responded to (i.e. Reviewer 3).

I am a bit confused about the authors' response to Reviewer 3, as detailed below:

1. In their response, the authors said that "We have added a total of 12 HiChIP, 10 ChIA-PET and 20 ChIP-seq datasets in the same manner as we did for mESC-Smc1". But, in the results section, the authors say "We focused on the mESC-SMC1a (Smc1) and K562-H3K27ac datasets for the analysis, which were performed in the same cell type, and with the same antibody for the HiChIP and ChIA-PET experiments." I think the authors should show clearly that they actually analyzed 12 HiChIP, 10 ChIA-PET and 20 ChIP-seq datasets and also show clearly this data.

2. The amount of data presented in Figure 1 is more than the data shown in Response Figure 1. For clarity, I think it would be good to present the data from the 12 HiChIP, 10 ChIA-PET and 20 ChIP-seq datasets in the also in the same manner as you did for mESC-Smc1.

3. It would also be better to discuss these 12 HiChIP, 10 ChIA-PET and 20 ChIP-seq datasets in methods section, to give their references and their characteristics. I can see the smc1 and H3K27ac data discussed in the methods section, but not the rest of these additional datasets.

4. Within Figure 1, although the authors said they performed the analysis with SMC1 and H3K27ac datasets, I am not clear as to which figure panels were performed with which dataset, or if they were performed with both datasets? I think it would be clearer if the SMC1 and H3K27ac data were presented separately, and clearly marked with their identities.

5. I agree with Reviewer 3 that all the scripts should be made available on github & zenodo for reproducibility. I looked at the github page (https://github.com/CSUBioGroup/Bacon) and I am quite confused about the different scripts. There are 5 different folders and most of them just say "updating gold loops and source code" - I do not think every single folder can indeed be about the same thing. The readme file is also very hard to follow. For example, I cannot see how to run the simulation at all. The authors should make an effort to prepare a comprehensive readme and well organized github page so that people who want to reproduce this method do not need to ask the authors for help.

**Authors Response**

**Point-by-point responses to the reviewers’ comments:**

Reviewer #1:

I am re-reviewing this manuscript in order to assess whether the other reviewers' comments have been responded to (i.e. Reviewer 3).

I am a bit confused about the authors' response to Reviewer 3, as detailed below:

1. In their response, the authors said that "We have added a total of 12 HiChIP, 10 ChIA-PET and 20 ChIP-seq datasets in the same manner as we did for mESC-Smc1". But, in the results section, the authors say "We focused on the mESC-SMC1a (Smc1) and K562-H3K27ac datasets for the analysis, which were performed in the same cell type, and with the same antibody for the HiChIP and ChIA-PET experiments." I think the authors should show clearly that they actually analyzed 12 HiChIP, 10 ChIA-PET and 20 ChIP-seq datasets and also show clearly this data.

***Response****: Thanks for the suggestions. And we agree it makes sense to focus on the larger analysis, especially given that the results from this analysis are consistent with both the SMC1 and H3K27ac-specific dataset investigations. We have added a total of 12 HiChIP, 10 ChIA-PET and 20 ChIP-seq datasets and analyzed in the same manner as we previously did for mESC-Smc1 (****Response Fig.1****). And we have moved the MboI restriction site distribution analysis output from these more diverse datasets to Figure 1C. Also, the evaluation of the degree of overlap between ChIP-seq, HiChIP, and ChIA-PET from all datasets has been added to Figure 1D-1F. Further, we performed differential peak analysis on the larger group of datasets, replacing the focused SMC1 analysis. We have replaced* ***Figure1*** *with* ***Response Fig.1 (below)*** *in the revised manuscript. Thus, in the updated manuscript we have focused main Figure 1 on the analysis of the 12 HiChIP, 10 ChIA-PET, and 20 ChIP-seq datasets.*

 ***Response Figure 1.*** *Data characteristics of ChIA-PET and HiChIP. (A) PCA plot for replicates of mESC-Smc1 ChIP-seq, ChIA-PET, HiChIP reads (B) PCA plot for replicates of K562-H3K27ac ChIP-seq, ChIA-PET, HiChIP reads. (C) The counts distribution of Mbol restriction enzyme sites across different peak types on 12 HiChIP, 10 ChIA-PET and 20 ChIP-seq datasets. (D) The overlap between HiChIP peaks and Mbol restriction sites across 12 HiChIP datasets. (E) The overlap between ChIP-seq peaks and Mbol restriction sites across 20 ChIP-seq datasets. (F) The overlap between ChIA-PET peaks and Mbol restriction sites across 10 ChIA-PET datasets. (G, H) Differential volcano plots comparing ChIP-seq with ChIA-PET and HiChIP peaks quantitatively on 12 HiChIP, 10 ChIA-PET and 20 ChIP-seq datasets. (I) Peak co-occupancy between ChIP-seq/ChIA-PET, and ChIP-seq/HiChIP on 12 HiChIP, 10 ChIA-PET and 20 ChIP-seq datasets. Bar plot drawn with standard error bar. ***, p-value < 1e-3, p-value was calculated by t-test.*

*We have also modified the Results as follows:*

*“****Data characteristics of ChIA-PET and HiChIP***

*HiChIP and ChIA-PET both produce similar information about protein-specific topological interactions and are interpreted in much the same way. However, the two protocols differ greatly in how they capture this information. One important distinction between the two, is that HiChIP incorporates restriction endonucleases to fragment the genome, while ChIA-PET traditionally relies upon sonication. And while this difference has been taken into account by some computational pipelines [28], a full characterization of the differences and similarities that exist between HiChIP and ChIA-PET is lacking. To characterize the read properties of HiChIP and ChIA-PET libraries, we compared their alignments with publicly available ChIP-seq data (for the processing of ChIP-seq data, see methods). Principal Component Analysis (PCA) showed that the ChIP-seq, ChIA-PET, and HiChIP replicates clustered together into their respective experimental groups (Fig. 1A-B) (for PCA analysis, see methods). Notably, mESC-Smc1 HiChIP data was heavily impacted by restriction enzyme treatment, as the read distribution of mESC-Smc1 HiChIP presented restriction enzyme cut site bias. Correspondingly, mESC-Smc1 HiChIP reads were sparse in positions without Mbol restriction sites (Additional file 1: Fig. S1A). To compare the bias of these two methodologies in a quantitative manner, we called peaks for 20 ChIP-seq, 10 ChIA-PET and 12 HiChIP datasets separately, and then calculated the distribution of Mbol restriction enzyme motifs near peaks. We found the counts distribution of restriction enzyme sites also showed more enzyme sites located within HiChIP peaks (Fig. 1C). Overall, 58.9% of HiChIP peaks overlapped with Mbol restriction enzyme sites (Fig. 1D-1F), which was much greater than the 22.9% observed for ChIA-PET.*

*An important control for evaluating HiChIP and ChIA-PET experiments is a favorable overlap with ChIP-seq data. We next performed differential analysis between 10 ChIA-PET, 12 HiChIP, and 20 ChIP-seq datasets (See methods). A total of 5,046 peaks that differed significantly between ChIA-PET and ChIP-seq were identified, and 3,549 of these were ChIA-PET enriched (Fig. 1G). Conversely, 29,136 peaks differed between ChIP-seq and HiChIP datasets, among these 26,222 were HiChIP-specific (Fig. 1H). We then looked at the peaks that each dataset had in common with ChIP-seq, and found that 41.6% of ChIA-PET peaks overlapped with ChIP-seq peaks compared to 10% overlap of ChIP-seq with HiChIP peaks (Fig. 1I). Hence, HiChIP data generates more loops with elevated sensitivity compared to ChIA-PET, however, HiChIP produces data with a strong restriction enzyme site bias and a lower overall agreement with ChIP-seq data than ChIA-PET.”*

2. The amount of data presented in Figure 1 is more than the data shown in Response Figure 1. For clarity, I think it would be good to present the data from the 12 HiChIP, 10 ChIA-PET and 20 ChIP-seq datasets in the also in the same manner as you did for mESC-Smc1.

***Response****: please see response #1.*

3. It would also be better to discuss these 12 HiChIP, 10 ChIA-PET and 20 ChIP-seq datasets in methods section, to give their references and their characteristics. I can see the smc1 and H3K27ac data discussed in the methods section, but not the rest of these additional datasets.

***Response****: Thanks for the suggestions. We have added following to Methods section in revised manuscript.*

*“****Processing of ChIA-PET, HiChIP and ChIP-seq peaks***

*We gathered 10 ChIA-PET and 12 HiChIP raw datasets (for accession numbers, see https://csuligroup.com/Bacon), and the public ChIP-seq data were obtained from NCBI (GSE22562 [59], GSE31785 [60], GSE65698 [61], GSE112717 [54], GSE113429 [55], GSE23619 [62], GSE162739 [63], GSE143886[64]). The raw reads were aligned to mm10 or hg19 reference genomes with bowtie2 [67], using default parameters. For track visualization, the uniquely mapped reads were fed into the “bamCoverage” function of deeptools with “–binSize 10 –normalizeUsing RPGC –effectiveGenomeSize 2150570000” to convert the bam alignment into bigwig. For detecting peak regions, MACS2 [32] was utilized to call peaks with “-q 0.01 -B –SPMR –keep-dup all”. Then the peaks from different datasets were merged with Bedtools if they contained at least 80% length overlap, and we counted reads from all the datasets over these peaks individually.”*

*We also modified the Methods of PCA and differential analysis in the revised manuscript as follows,*

*“****PCA and differential analysis for ChIP-seq, ChIA-PET and HiChIP***

*Given that ChIP-seq, HiChIP, and ChIA-PET all rely on chromatin immunoprecipitation, they should display similar binding profiles when comparisons are being made for a single protein of interest. We implemented DiffBind to perform PCA analysis and to give a deeper insight into how these experimental groups were associated. The alignment files derived from ChIP-seq, ChIA-PET, and HiChIP replicates were filtered using Samtools with minimum MAPQ 30. Picard was then used to remove duplicates, and the filtered bam files were prepared as the input to function “dba”, then a binding matrix with scores was calculated based on read counts for every sample with function “dba.count”. The data were normalized based on sequencing depth with default setting of function “dba.normalize”, then the function “dba.plotPCA” was used to see how well the samples cluster with one another.*

*Before running the differential analysis, we used “DownsampleSam” function of Picard with “P=0.2” to downsample the alignment files. “dba.contrast” function with default mode to model the data, as well as specify the comparisons we interested in are ChIP-seq vs ChIA-PET and ChIP-seq vs HiChIP. Then “dba.analyze” function was used to perform the differential analysis, and the p-value <0.05 and log2(fold change)>1 was used as the threshold of significance to detect differential peaks for one of the antibody. We repeated the differential analysis for ChIP-seq vs ChIA-PET and ChIP-seq vs HiChIP on every antibody, then all the differential peaks were aggregated.”*

4. Within Figure 1, although the authors said they performed the analysis with SMC1 and H3K27ac datasets, I am not clear as to which figure panels were performed with which dataset, or if they were performed with both datasets? I think it would be clearer if the SMC1 and H3K27ac data were presented separately, and clearly marked with their identities.

***Response****: Please see response #1. And in Figure 1, we have separated the PCA plots for H3K27ac and SMC1 (****Response Figure 1A, and 1B****) and added additional labels to distinguish these datasets more clearly.*

5. I agree with Reviewer 3 that all the scripts should be made available on github & zenodo for reproducibility. I looked at the github page (https://github.com/CSUBioGroup/Bacon) and I am quite confused about the different scripts. There are 5 different folders and most of them just say "updating gold loops and source code" - I do not think every single folder can indeed be about the same thing. The readme file is also very hard to follow. For example, I cannot see how to run the simulation at all. The authors should make an effort to prepare a comprehensive readme and well organized github page so that people who want to reproduce this method do not need to ask the authors for help.

***Response****: Thanks for the suggestions. We have added more details to readme file and re-organized the GitHub page to make it easier to follow.*
